# Vanadium Cation Exchange‐Driven Reconstruction of MOF‐Derived Cobalt Hydroxide Electrodes for Electrocatalysis and Energy Storage

**DOI:** 10.1002/advs.76610

**Published:** 2026-07-14

**Authors:** Yongbeen Kim, Seungwoo Han, Wonyoung Lee

**Affiliations:** ^1^ School of Mechanical Engineering Sungkyunkwan University (SKKU) Suwon Republic of Korea; ^2^ SKKU Institute of Energy Science and Technology (SIEST) Sungkyunkwan University Suwon Gyeonggi‐do Republic of Korea

**Keywords:** cation exchange, lattice oxygen oxidation mechanism, oxygen evolution reaction, oxygen vacancy

## Abstract

The development of earth‐abundant oxygen evolution reaction (OER) electrocatalysts remains challenging because structural reconstruction and electronic/defect modulation are often addressed separately. Herein, a room‐temperature vanadium cation exchange strategy that enables structural reconstruction and electronic/defect modulation is reported. Vanadium cation exchange removes organic ligands and drives the phase transformation to mesoporous cobalt hydroxide using ZIF‐67 grown on nickel foam, while high‐valence vanadium incorporation increases the cobalt oxidation state and promotes oxygen vacancy formation. The optimized V_20–_Co(OH)_2_ electrode exhibits current densities of 50 mA cm^−2^ and 100 mA cm^−2^ at overpotentials of 268 and 293 mV, respectively, in 1.0 M KOH, and maintains stable operation for 150 h. In situ Raman and post‐stability analyses reveal the formation of a CoOOH‐like active surface during OER operation, while oxygen vacancy‐related features and a high cobalt oxidation state are largely retained after operation. Spectroscopic, electrochemical, and kinetic analyses reveal an increased contribution from lattice oxygen participation during OER. The defect‐engineered electrode exhibits enhanced performance in supercapacitors and zinc–air batteries, demonstrating a generalizable electrode engineering strategy for energy storage and conversion systems.

## Graphical abstract

A room‐temperature vanadium cation exchange strategy is developed to achieve simultaneous structural reconstruction and electronic/defect modulation of ZIF‐67 on nickel foam. The resulting mesoporous V_20_–Co(OH)_2_ electrode exhibits superior OER activity with low overpotentials and enhanced lattice oxygen participation. This generalizable defect‐engineering approach demonstrates high efficiency for advanced energy storage and conversion systems.

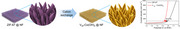

## 1 Introduction

Electrochemical water splitting is crucial for achieving sustainable hydrogen production [1, 2, 3, 4, 5]. However, the oxygen evolution reaction (OER) remains kinetically demanding and requires a substantial overpotential owing to its multi‐electron nature [6, 7, 8]. Although noble metal oxides, such as ruthenium and iridium oxides, show high intrinsic OER activity, their cost and limited availability necessitate the development of earth‐abundant alternatives [8, 9]. Transition metal‐based (oxy)hydroxides have emerged as promising OER catalysts in alkaline electrolytes, where the reaction kinetics are governed by the energetics of the oxygenated intermediates (*OH, *O, and *OOH) and the local electronic structure of the active sites [10, 11, 12]. Beyond the conventional adsorbate evolution mechanism (AEM), which proceeds through surface‐bound intermediates and is constrained by the *O → *OOH step, the lattice oxygen mechanism (LOM) can partially bypass this barrier and contribute to accelerated OER kinetics under appropriate metal–oxygen bonding and defect environments [7, 13, 14, 15, 16]. However, lattice oxygen‐related processes are difficult to implement in a controlled and stable manner because electronic/defect modulation and structural engineering are typically addressed in separate and sequential strategies.

In current catalyst designs, balancing the accessible surface area with precise electronic and defect modulation is difficult to achieve. Structural modification can effectively increase the number of exposed sites; however, it does not necessarily provide an electronic configuration favorable for lattice oxygen participation [17, 18, 19]. Moreover, defect formation and electronic modulation are often achieved through high‐temperature treatments or harsh chemical processes, which can induce undesired phase evolution, pore collapse, and non‐uniform defect distribution [20, 21, 22, 23, 24]. These issues are critical in electrode architectures, where the interfacial charge transfer and electrochemically accessible surface area determine the overall performance [25, 26, 27]. Therefore, a synthesis framework that integrates structural reconstruction with controlled electronic and defect modulation under moderate conditions is essential.

Metal‐organic frameworks (MOFs) are attractive because of their ordered porosity, uniform metal site distribution, and compositional tunability [28, 29, 30, 31, 32]. However, the direct application of MOFs to the OER is hindered by insulating organic ligands and limited conductivity, and conventional thermal conversion can compromise the intrinsic porous architecture [25, 33, 34]. Introducing high‐valence dopants is an alternative route for regulating electronic structures; however, the resulting charge compensation pathways depend strongly on the dopant identity. High‐valence 4d/5d dopants typically alleviate charge imbalance primarily through electron redistribution; however, vanadium, a 3d transition metal with high electronic flexibility, can promote coupled metal oxidation and oxygen vacancy formation through a local bonding mismatch [35, 36, 37]. Nevertheless, quantitative correlations between vanadium incorporation, oxygen vacancy formation, electrochemically accessible surface area, and the electrochemical and kinetic characteristics associated with lattice oxygen‐related processes remain insufficiently established in MOF‐derived systems.

In this study, we report a room‐temperature vanadium cation exchange strategy that converts zeolitic imidazolate framework‐67 (ZIF‐67) grown on nickel foam into a mesoporous cobalt hydroxide electrode, while concurrently enabling electronic and defect modulation. Vanadium cation exchange removes organic ligands and drives the phase transformation to Co(OH)_2_, while high‐valence vanadium incorporation increases the cobalt oxidation state and promotes oxygen vacancy formation. The optimized V_20_–Co(OH)_2_ electrode exhibits current densities of 50 mA cm^−2^ and 100 mA cm^−2^ at overpotentials of 268 and 293 mV, respectively, in 1.0 M KOH, and maintains stable operation for 150 h. In situ Raman and post‐stability analyses reveal the formation of a CoOOH‐like active surface during OER operation, while oxygen vacancy‐related features and a high cobalt oxidation state are largely retained after operation. Quantitative correlations between oxygen vacancy concentration, electrochemically accessible surface area, interfacial charge‐transfer characteristics, and mechanistic probes support an increased contribution from lattice oxygen participation during OER. In addition to oxygen electrocatalysts, defect‐engineered electrodes exhibit enhanced performance in supercapacitors and zinc–air batteries. Overall, the proposed synthesis strategy and mechanistic insights provide a versatile and scalable platform applicable to various energy storage and conversion systems, offering a new pathway for developing low‐cost high‐efficiency electrocatalysts.

## 2 Results and Discussion

**FIGURE 1:**
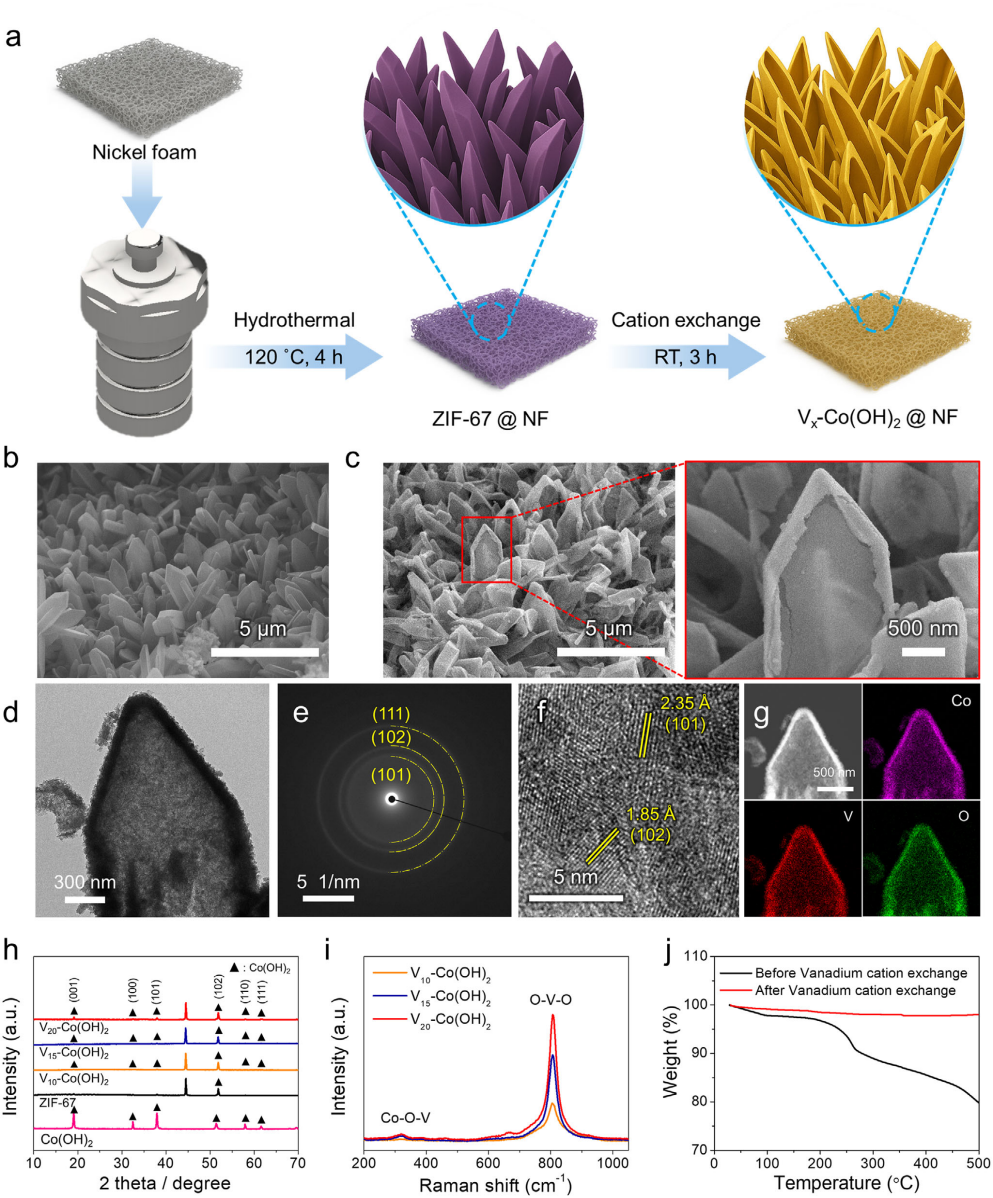
Structural reconstruction of ZIF‐67 induced by vanadium cation exchange. (a) Schematic of the synthesis of vanadium cation‐exchange‐derived vanadium‐exchanged Co(OH)_2_ grown on nickel foam. (b) FE‐SEM image of pristine ZIF‐67. (c) FE‐SEM image of reconstructed vanadium‐exchanged Co(OH)_2_ after vanadium cation exchange. (d) TEM image, (e) SAED pattern, and (f) HR‐TEM image of vanadium‐exchanged Co(OH)_2_. (g) STEM image and corresponding elemental mapping images of Co, V, and O. (h) XRD patterns, (i) Raman spectroscopy, and (j) TGA profiles of vanadium‐exchanged Co(OH)_2_.

Figure 1 shows that vanadium cation exchange induces pronounced structural reconstruction of ZIF‐67, yielding a porous architecture with an increased accessible surface area. ZIF‐67 was selected as the sacrificial template because its well‐defined porous framework enables controlled reconstruction while preserving the macroscopic integrity. As illustrated in Figure 1a, ZIF‐67 is uniformly grown on the nickel foam via a hydrothermal process to form a binder‐free and electrically conductive electrode. Subsequent room‐temperature cation exchange with vanadium precursors enabled simultaneous ligand removal and phase transformation, converting the parent ZIF‐67 into a vanadium‐doped cobalt hydroxide framework. By varying the vanadium precursor concentration (10, 15, and 20 at.%), three electrodes were prepared, denoted as V_10_–Co(OH)_2_, V_15_–Co(OH)_2_, and V_20_–Co(OH)_2_, respectively. Their stoichiometric compositions were quantified using inductively coupled plasma mass spectrometry (ICP–MS) (Table S1).

Field emission scanning electron microscopy (FE‐SEM) revealed the morphological evolution associated with the cation‐exchange process. Pristine ZIF‐67 exhibited a plate‐like morphology with an average lateral size of ∼1 µm (Figure 1b and Figure S1). After vanadium cation exchange, the overall plate‐like morphology was preserved, while partially peeled and concave surfaces appeared (Figure 1c and Figure S2a–d). This reconstructed morphology can be attributed to ligand removal accompanied by lattice rearrangement during vanadium incorporation. Similar morphologies were retained across different vanadium concentrations, whereas excessive vanadium exchange resulted in partial framework collapse (Figure S2e,f). Transmission electron microscopy (TEM) verified the preservation of the plate‐like morphology before and after reconstruction (Figure 1d and Figure S3). The selected area electron diffraction (SAED) pattern (Figure 1e) exhibited polycrystalline rings indexed to the (101) and (102) planes of Co(OH)_2_, consistent with the lattice fringes of 0.235 and 0.185 nm observed in the high‐resolution TEM (HRTEM) image (Figure 1f), respectively. Elemental mapping (Figure 1g and Figure S4) demonstrated a homogeneous distribution of Co, V, and O throughout the reconstructed structure, indicating uniform vanadium incorporation without the formation of vanadium‐enriched domains [38]. X‐ray diffraction patterns (XRD; Figure 1h and Figure S5) clearly exhibit phase transformation. After the cation exchange, new diffraction peaks appeared at 37.9°, 52.7°, and 62.3°, corresponding to the (101), (102), and (111) planes of Co(OH)_2_, respectively. No additional peaks associated with crystalline vanadium oxides were detected across the investigated vanadium concentrations, confirming the single‐phase nature of vanadium‐exchanged Co(OH)_2_. The local chemical bonding states were further examined using Raman spectroscopy. A distinct vibration band at 816 cm^−^^1^ appeared for all vanadium‐exchanged electrodes, which can be assigned to the V─O stretching mode, confirming that vanadium was incorporated into the Co(OH)_2_ lattice rather than physical adsorption on the surface (Figure 1i) [39, 40]. With increasing vanadium concentration, the intensities corresponding to the O─V─O and Co─O─V stretching modes increased, which is consistent with the progressive incorporation of vanadium into the cobalt‐based framework. However, at excessively high vanadium concentration, emergence of a CoO secondary phase was observed, indicating partial structural degradation (Figure S6) [41, 42].

The removal of organic ligands during cation exchange was quantitatively verified using thermogravimetric analysis (TGA) (Figure 1j). Pristine ZIF‐67 exhibited pronounced mass loss between 200 and 300°C, corresponding to the decomposition of organic ligands. In contrast, all vanadium‐exchanged electrodes showed negligible mass loss (< 0.1%) within the same temperature range (Figure S7), indicating that organic ligands were effectively eliminated during room‐temperature cation exchange. This behavior is consistent with previous reports that MOF ligands desolvate below ∼350°C, followed by framework degradation at higher temperatures, which is absent in the reconstructed electrodes [43]. The textural properties of the reconstructed structures were examined using a Brunauer–Emmett–Teller (BET) analysis (Figure S8). Compared with pristine ZIF‐67, the vanadium‐exchanged electrodes exhibited an increased specific surface area and clear mesoporous characteristics. In particular, the V_20_–Co(OH)_2_ electrode exhibited a specific surface area of 6.87 m^2^ g^−1^, an increase of 350% compared to pristine ZIF‐67 (1.52 m^2^ g^−1^). The Barrett–Joyner–Halenda pore size distribution (Figure S9) revealed enlarged mesopores after cation exchange, indicating that ligand removal created accessible porosity. These results indicate that vanadium cation exchange functions beyond a simple compositional modification, providing a practical room‐temperature strategy for ligand removal and phase transformation and yielding a mesoporous cobalt hydroxide architecture with structurally favorable characteristics for subsequent electronic and defect modulation.

To elucidate the electronic structure and local coordination environment of the reconstructed Co(OH)_2_ framework induced by vanadium cation exchange, systematic x‐ray photoelectron spectroscopy (XPS) and x‐ray absorption spectroscopy (XAS) analyses were conducted. This investigation focused on the changes in cobalt valence states and local oxygen coordination induced by the incorporation of vanadium.

**FIGURE 2:**
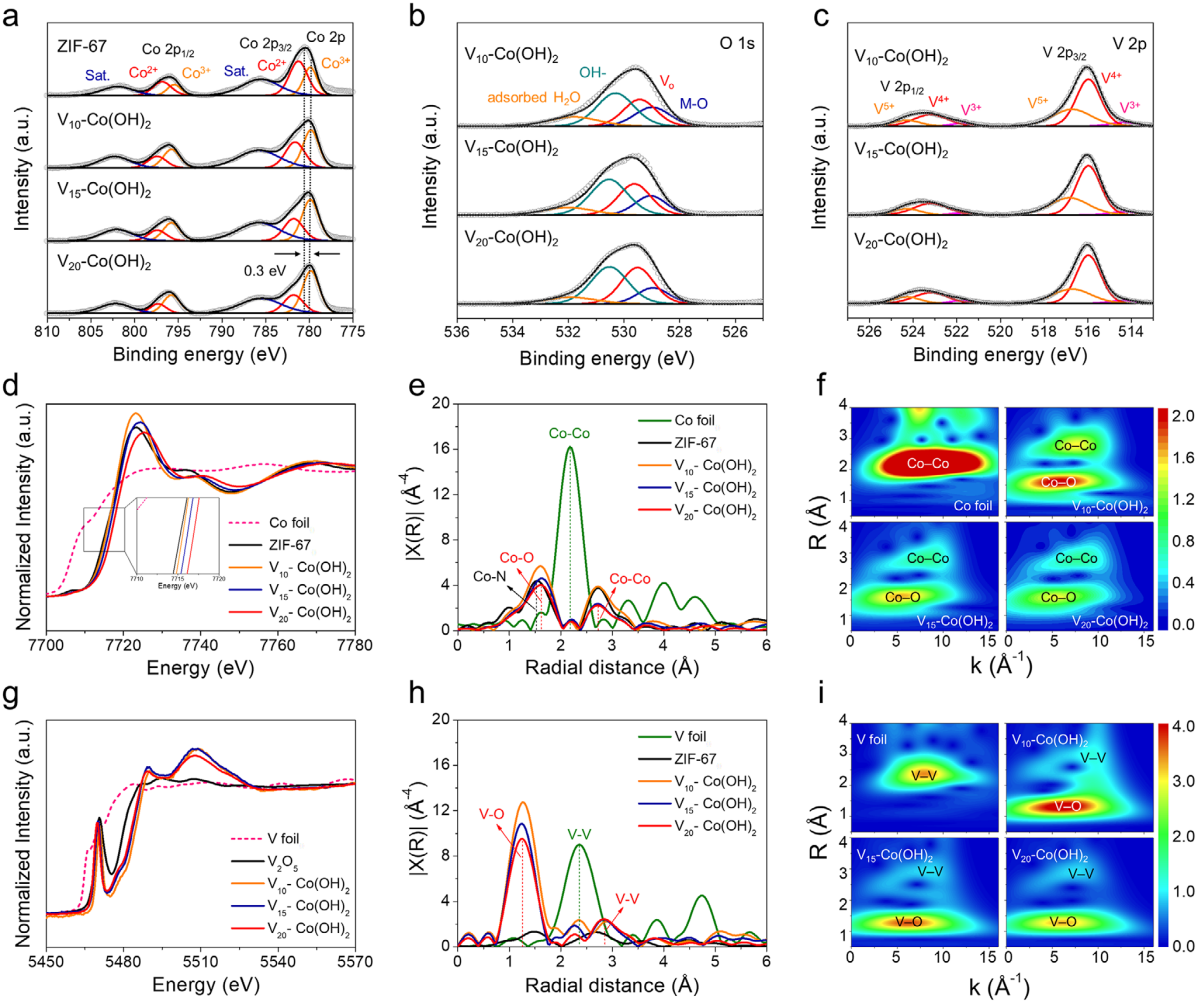
Electronic structure and local coordination evolution induced by vanadium cation exchange. (a) Co 2p XPS spectra of pristine ZIF‐67 and vanadium‐exchanged Co(OH)_2_ electrodes with different vanadium concentrations. (b) O 1s XPS spectra of vanadium‐exchanged Co(OH)_2_ electrodes. (c) V 2p XPS spectra of vanadium‐exchanged Co(OH)_2_ electrodes. (d) Co K‐edge XANES spectra of pristine ZIF‐67 and vanadium‐exchanged Co(OH)_2_ electrodes. (e) Co K‐edge FT‐EXAFS spectra of pristine ZIF‐67 and vanadium‐exchanged Co(OH)_2_ electrodes. (f) Co K‐edge WT‐EXAFS contour plots of Co foil and vanadium‐exchanged Co(OH)_2_ electrodes. (g) V K‐edge XANES spectra of vanadium‐exchanged Co(OH)_2_ electrodes. (h) V K‐edge FT‐EXAFS spectra of vanadium‐exchanged Co(OH)_2_ electrodes. (i) V K‐edge WT‐EXAFS contour plots of V foil and vanadium‐exchanged Co(OH)_2_ electrodes.

Survey XPS spectra confirmed the coexistence of Co, V, and O in all the vanadium‐exchanged electrodes (Figure S10), verifying successful vanadium incorporation. The Co 2p XPS spectra (Figure 2a) can be deconvoluted into two spin–orbit doublets centered at 781.7/796.6 and 779.6/795.5 eV, corresponding to Co^2+^ and Co^3+^, respectively, along with characteristic shake‐up satellite peaks [39, 44]. Pristine ZIF‐67 was dominated by Co^2+^, while the minor presence of Co^3+^ originated from surface oxidation upon air exposure [45]. After the vanadium cation exchange, the Co^3+^/Co^2+^ ratio of all the vanadium‐exchanged electrodes increased substantially (Figure S11), indicating the oxidation of the cobalt species. This change is attributed to the incorporation of high‐valence vanadium, which acts as an electron acceptor and promotes charge redistribution within the Co(OH)_2_ lattice, resulting in an increase in the average oxidation state of cobalt with increasing vanadium concentration. The evolution of the oxygen coordination environment was examined using O 1s XPS (Figure 2b). The spectra were deconvoluted into four components centered at 529.6, 529.6, 530.9, and 532.5 eV, which were assigned to lattice oxygen, defect‐associated oxygen, hydroxyl‐associated species, and adsorbed hydroxyl/H_2_O species, respectively [46, 47]. V_20_–Co(OH)_2_ exhibited the highest relative contribution of defect‐associated oxygen (Figure S12), indicating the formation of the most pronounced oxygen vacancy. The coexistence of the Co─O and V─O bonding environments, coupled with their distinct bond lengths, can induce local lattice distortion, promoting oxygen vacancy formation [48, 49]. The oxidation state and coordination of vanadium were analyzed using the V 2p XPS spectra (Figure 2c). The V 2p_3/2_ peak was deconvoluted into three components centered at 514.12, 515.88, and 516.44 eV, corresponding to V^3+^, V^4+^, and V^5+^, respectively. Vanadium predominantly exists in higher oxidation states (+4 and +5), with a minor fraction of V^3+^ [50]. Notably, the distribution of the vanadium charge states remained nearly constant across different compositions, indicating that vanadium primarily served as an electronic modulator rather than an active redox center. This behavior supports its role in stabilizing cobalt oxidation and forming oxygen vacancies.

To probe the bulk electronic structure and local coordination beyond the surface‐sensitive XPS analysis, Co K‐edge x‐ray absorption near‐edge structure (XANES) and extended x‐ray absorption fine structure (EXAFS) analyses were performed. The Co K‐edge adsorption edge shifted toward higher energies after vanadium exchange (Figure 2d), confirming an increase in the average Co oxidation state. The highest oxidation state was observed for V_20_–Co(OH)_2_, which is in agreement with the XPS results. Fourier‐transformed Co K‐edge EXAFS spectra (Figure 2e) reveal distinct coordination shells corresponding to Co─O and Co─Co interactions. In contrast to pristine ZIF‐67, which is characterized primarily by Co─N coordination, the vanadium cation exchange induced a clear transition to a dominant Co─O coordination environment, which is consistent with the phase transformation identified in Figure 1 [51, 52]. For V_20_–Co(OH)_2_, the peaks at 1.62 Å and 2.71 Å were assigned to Co─O and Co─Co shells, respectively. With increasing vanadium concentration, the Co─O coordination intensity gradually decreased, indicating a local structural distortion associated with the formation of oxygen vacancies. This was supported by the EXAFS curve‐fitting results, which showed a systematic decrease in the Co─O and Co─Co coordination numbers with increasing vanadium concentration (Figures S13 and S14 and Table S2). Wavelet transform (WT)‐EXAFS analysis was employed to resolve coordination environments in k and R spaces. Using a Morlet wavelet basis, the Co K‐edge WT contour plot of V_20_–Co(OH)_2_ exhibited intensity maxima at 5.3 and 7.9 Å^−1^ (Figure 2f), corresponding to Co─O and Co─Co bonds, respectively [53]. The relative attenuation of the Co─O intensity with increasing vanadium concentration supports the formation of oxygen vacancies, consistent with the XPS and EXAFS analyses. The local coordination environment of vanadium was examined using V K‐edge XANES and EXAFS. The V K‐edge XANES spectra exhibited a distinct pre‐edge peak at ∼5470 eV (Figure 2g), attributed to the 1s → 3d transition of V^4+^, indicating enhanced V─O covalency. The predominant oxidation state of vanadium was consistent with the XPS results [54]. The Fourier‐transformed V K‐edge EXAFS spectra (Figure 2h) revealed distinct V─V and V─O coordinations [39]. Notably, V_20_─Co(OH)_2_ exhibited a shorter V─O bond length than the electrodes with lower vanadium concentrations (Figures S15 and S16 and Table S3), suggesting that high‐valence vanadium substitutes cobalt sites and attracts additional oxygen, thereby facilitating the formation of oxygen vacancies in the surrounding lattice. The corresponding WT‐EXAFS contour plots (Figure 2i) showed intensity maxima at 5.95 Å^−1^ and 7.4 Å^−1^, assigned to V─O and V─V coordination, respectively.

**FIGURE 3:**
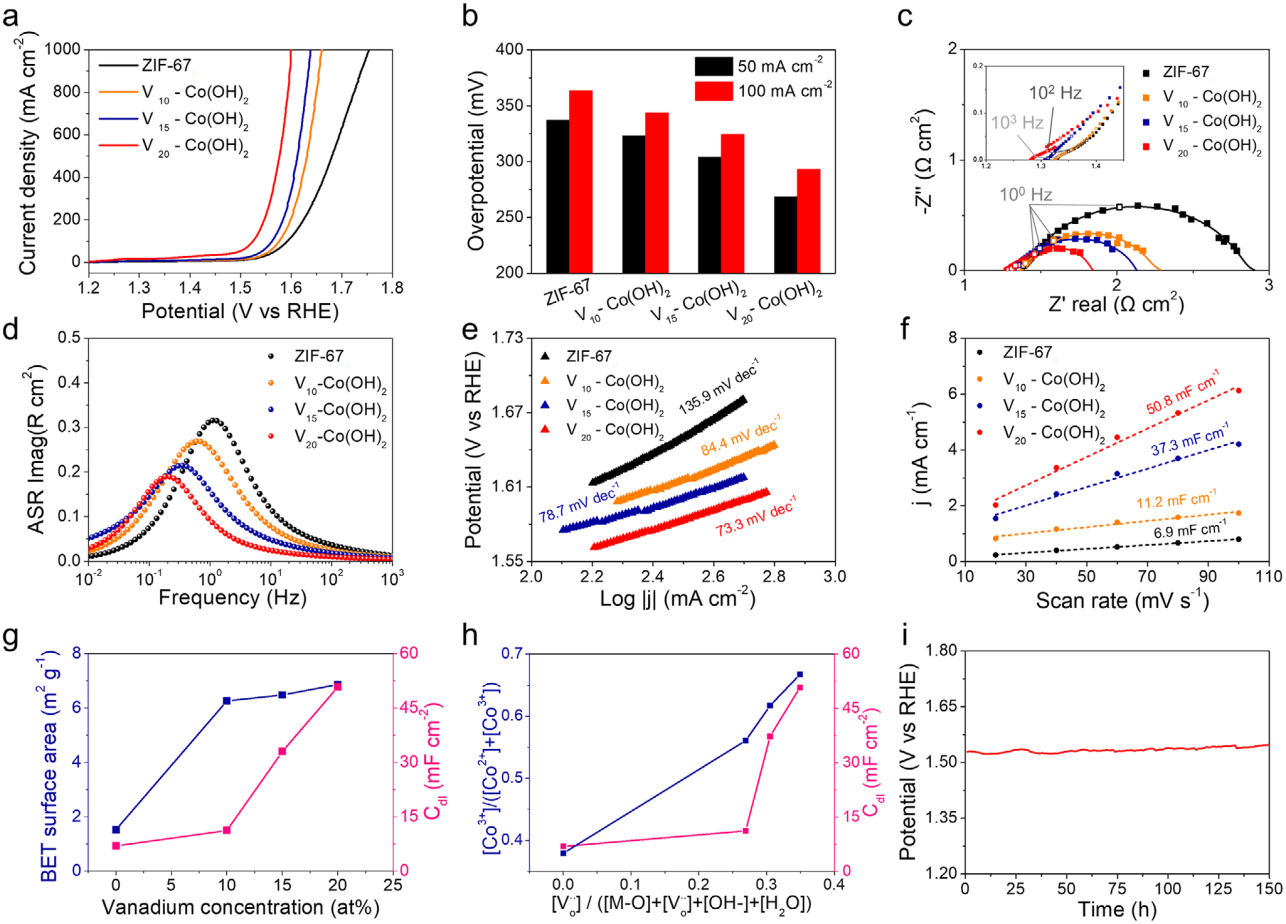
Correlation between electronic/defect structure and electrochemical performance in 1.0 M KOH. (a) iR‐corrected LSV curves. (b) Corresponding overpotentials at 50 and 100 mA cm^−2^. (c) EIS spectra at 1.58 V vs. RHE. (d) Bode plots at 1.63 V vs. RHE. (e) Tafel plots. (f) Capacitive current density at 0.05 V as a function of scan rate. (g) BET surface area (blue) and C_dl_ (pink) as a function of vanadium concentration. (h) Relationship between oxygen vacancy concentration derived from O 1s XPS (blue), C_dl_ (pink), and vanadium concentration. (i) Chronopotentiometric stability test of V_20_–Co(OH)_2_ at a constant current density of 100 mA cm^−2^.

Based on the structural reconstruction and electronic modulation, electrochemical measurements were conducted to evaluate the influence of these changes on the OER performance. The electrocatalytic activities of pristine ZIF‐67 and vanadium‐exchanged electrodes were assessed in 1.0 M KOH using a conventional three‐electrode configuration. The iR‐corrected linear sweep voltammetry (LSV) curves (Figure 3a) showed that pristine ZIF‐67 exhibited sluggish OER activity, requiring overpotentials of 337 and 364 mV to achieve current densities of 50 and 100 mA cm^−2^, respectively. In contrast, all vanadium‐exchanged electrodes displayed substantially enhanced activity. Among them, V_20_–Co(OH)_2_ achieved the same current densities at significantly lower overpotentials of 268 and 293 mV, respectively (Figure 3b). When compared with representative transition metal‐based OER electrodes reported in the literature, V_20_–Co(OH)_2_ exhibited a competitive overpotential at current densities of 50 mA cm^−2^ and 100 mA cm^−2^ in 1.0 M KOH (Tables S4 and S5). Electrochemical impedance spectroscopy (EIS) revealed the enhanced reaction kinetics of the vanadium‐exchanged electrodes. In particular, V_20_–Co(OH)_2_ exhibited the smallest semicircle diameter in the Nyquist plot (Figure 3c), corresponding to the lowest charge‐transfer resistance (R_ct_). The Nyquist plots were fitted using an equivalent circuit model, as shown in Figure S17. At an overpotential of 350 mV, the R_ct_ value was 0.48 Ω cm^−2^, significantly lower than that of pristine ZIF‐67 and other vanadium‐exchanged electrodes. EIS measurements conducted by increasing the applied bias from 1.48 to 1.73 V vs. reversible hydrogen electrode (RHE; Figures S18 and S19 and Table S6) showed a continuous decrease in R_ct_ for V_20_–Co(OH)_2_, indicating the promotion of electron transfer and reactant adsorption. In addition, the characteristic frequency peak in the Bode plots shifted toward lower frequencies for the vanadium‐exchanged electrodes compared to the pristine ZIF‐67 (Figure 3d), suggesting changes in the interfacial charge‐transfer reaction [55]. The improved reaction kinetics of the vanadium‐exchanged electrodes were further supported by the Tafel analysis (Figure 3e). The Tafel slope gradually decreased with increasing vanadium concentration, exhibiting 73.3 mV dec^−1^ for V_20_–Co(OH)_2_, compared with 135.9 mV dec^−1^ for pristine ZIF‐67, 84.4 mV dec^−1^ for V_10_–Co(OH)_2_, and 78.7 mV dec^−1^ for V_15_–Co(OH)_2_. The lower Tafel slope of V_20_–Co(OH)_2_ indicates more favorable intrinsic OER kinetics, which is consistent with the enhanced charge‐transfer characteristics observed using EIS.

Double‐layer capacitance (C_dl_) measurements were conducted to distinguish the contributions of the physically and electrochemically active surface areas. Cyclic voltammetry (CV) in the non‐faradaic potential region (0.92–1.02 V, Figure S20) revealed that pristine ZIF‐67 exhibited the smallest capacitive current response, whereas vanadium‐exchanged electrodes display larger responses with increasing vanadium concentration. In particular, V_20_–Co(OH)_2_ exhibited the largest integrated CV area, indicating that it has the highest density of electrochemically accessible active sites. The C_dl_ values extracted from the linear fitting of the capacitive current as a function of the scan rate (Figure 3f) were consistent with this trend, further confirming that V_20_–Co(OH)_2_ provided the largest electrochemically accessible surface area. Notably, when C_dl_ and the BET surface area were plotted as functions of the vanadium concentration (Figure 3g), a clear divergence was observed. The BET surface area reflects the physical surface area and porosity accessible to gas adsorption, whereas C_dl_ represents electrochemically accessible and redox‐active interfacial sites under electrolyte conditions. Although the BET surface areas of the vanadium‐exchanged electrodes remained comparable, C_dl_ increased monotonically with vanadium concentration, demonstrating that the enhancement in the electrochemically active surface area was not governed by the physical surface area but was strongly influenced by defect‐ and electronic structure‐mediated electrochemical accessibility. To correlate the defect chemistry with the electrochemical behavior, the oxygen vacancy concentrations derived from the O 1s XPS analysis were compared with the kinetic parameters (Figure 3h). Electrodes with higher oxygen vacancy concentrations exhibited higher C_dl_ values and Co^3+^ fractions, indicating a close association between defect formation, electronic structure modulation, and charge‐transfer kinetics. Consistently, FT‐EXAFS analysis (Figure S21) revealed a decrease in the M─O coordination number with increasing vanadium concentration, further supporting the presence of vanadium‐induced local structural distortion. These results indicate that vanadium cation exchange enhances the OER performance by simultaneously improving the intrinsic reaction kinetics and electrochemically active surface area through electronic modulation and defect‐driven surface activation, rather than through an increase in the physical surface area. Therefore, the superior activity of V_20_–Co(OH)_2_ was attributed to the synergetic effects of elevated cobalt oxidation states, enhanced M─O covalency, and defect‐driven surface activation. In addition, the durability of the optimized electrode was evaluated using chronopotentiometric test. V_20_–Co(OH)_2_ maintained a nearly constant potential at a current density of 100 mA cm^−2^ for 150 h (Figure 3h). In addition, negligible performance degradation was observed after 1000 cycles of LSV measurements (Figure S22), confirming robust structural and electrochemical stability under alkaline OER conditions.

**FIGURE 4:**
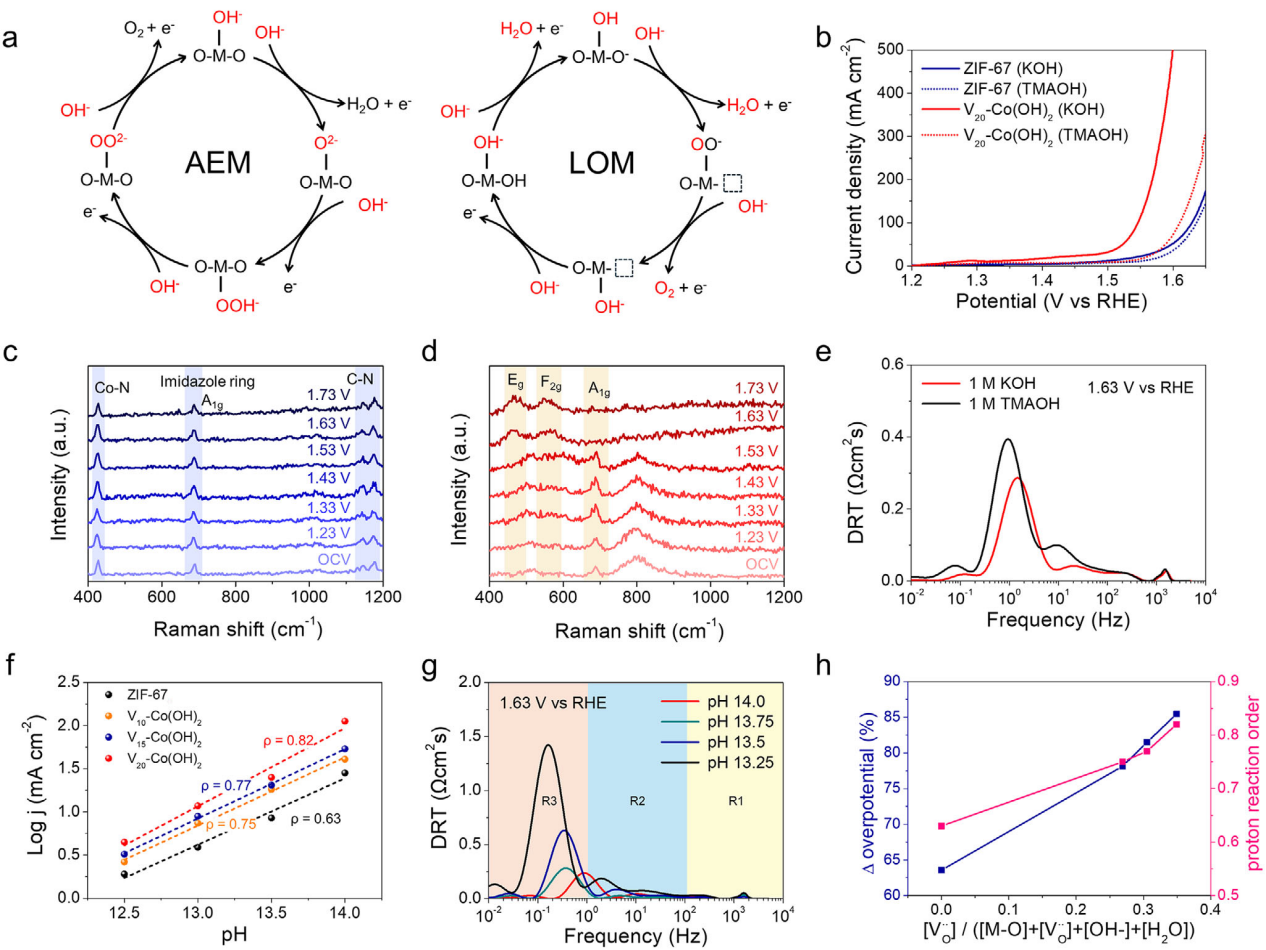
Evidence for lattice oxygen participation and identification of active sites. (a) Schematic of AEM and LOM for the OER mechanism. (b) iR‐corrected LSV curves of pristine ZIF‐67 and V_20_–Co(OH)_2_. In situ Raman spectra of (c) pristine ZIF‐67 and (d) V_20_‐Co(OH)_2_ in 1.0 M KOH electrolyte under increasing anodic bias from OCV to 1.73 V vs. RHE. (e) DRT analysis of pristine ZIF‐67 at 1.63 V vs. RHE in 1.0 M KOH and 1.0 M TMAOH electrolytes. (f) Current density at 1.55 V vs. RHE as a function of pH for pristine ZIF‐67 and V_20_–Co(OH)_2_ in KOH electrolytes (pH 12.5–14.0). (g) DRT analysis of pristine ZIF‐67 at 1.63 V vs. RHE in KOH electrolytes with different pH values (pH 13.25–14.0). (h) Correlation between overpotential degradation under different electrolytes (blue), proton reaction order (pink), and oxygen vacancy concentration derived from O 1s XPS.

Lattice oxygen participation is considered an effective pathway for accelerating OER kinetics, particularly when oxygen vacancies and strong metal–oxygen hybridization are present. The formation of oxygen vacancies can modify the local electronic structure and interfacial charge‐transfer characteristics, thereby facilitating lattice oxygen participation. Given the superior OER activity of V_20_–Co(OH)_2_, the contribution of lattice oxygen to the OER pathway was systematically investigated to clarify the underlying reaction mechanism. Figure 4a shows a schematic comparison of the AEM and LOM pathways. In the AEM pathway, the OER proceeds through surface‐adsorbed oxygenated intermediates, whereas in the LOM pathway, lattice oxygen participates directly in oxygen evolution, which is enabled by the enhanced metal–oxygen covalency and the presence of oxygen vacancies. Based on the defect‐rich electronic structures (Figures 2 and 3), the vanadium‐exchanged electrodes are expected to exhibit an increased LOM contribution.

To verify the lattice oxygen participation, tetramethylammonium (TMA^+^) cations were employed as chemical probes because of their strong electrostatic interactions with negatively charged peroxo‐ and superoxo‐like intermediates, which are key species in the LOM pathway [34, 53, 56]. As shown in Figure 4b, the current density of V_20_–Co(OH)_2_ at 1.55 V vs. RHE decreases by 85.46% from 111.84 mA cm^−2^ in 1.0 M KOH to 16.27 mA cm^−2^ in 1.0 M TMAOH. This pronounced suppression indicates that OER kinetics are strongly affected by pathway‐sensitive interfacial processes, consistent with increased contribution from lattice oxygen participation during OER operation. In contrast, pristine ZIF‐67 exhibited a relatively moderate decrease in the current density by 49.43% from 22.13 mA cm^−2^ in 1.0 M KOH to 11.19 mA cm^−2^ in 1.0 M TMAOH, suggesting weaker sensitivity to TMA^+^‐induced interfacial suppression (Figures S23 and S24 and Table S7). In addition, the characteristic Raman peaks of TMA^+^ at 752 cm^−1^ and 955 cm^−1^ became continuously intensified with increasing vanadium incorporation (Figure S25), indicating stronger interaction between TMA^+^ and the vanadium‐exchanged electrodes [34].

To further examine the surface evolution under OER conditions, in situ Raman spectroscopy was performed for pristine ZIF‐67 and the as‐prepared vanadium‐exchanged Co(OH)_2_ electrode. For pristine ZIF‐67, characteristic vibrational features associated with Co─N, imidazole ring/A_1g_, and C─N bonds were observed at 427, 690, and 1179 cm^−1^, respectively (Figure 4c) [57]. Pristine ZIF‐67 shows no clearly resolved CoOOH‐ or Co^3+^─O‐related vibrational features with increasing anodic bias, while Co^2+^─O‐related feature in 650–700 cm^−1^ is largely maintained. In contrast, the vanadium‐exchanged Co(OH)_2_ electrode exhibits distinct features at 475, 555, and 693 cm^−1^, which were assigned to E_g_, F_2g_, and A_1g_ vibrational modes associated with Co^2+^─O, Co^3+^─O, and Co^2+^─O bonding, respectively (Figure 4d) [58]. With increasing anodic bias, CoOOH‐related vibrational features gradually increase, accompanied by an increase in Co^3+^─O‐related features and a decrease in Co^2+^─O‐related features. These observations indicate oxidation of cobalt sites during OER operation, supporting the formation of a CoOOH‐like oxyhydroxide active surface from the vanadium‐exchanged Co(OH)_2_ electrode during OER operation. In addition, the O─V─O vibrational mode near ∼800 cm^−1^ continuously weakens under OER conditions, suggesting reconstruction of the vanadium‐associated local coordination environment, rather than simple physical adsorption of vanadium species. The evolution of E_g_ and F_2g_ vibration modes further supports dynamic lattice reconstruction during the OER process.

Post‐stability TEM and XPS analyses were further conducted to verify the structural and electronic characteristics after long‐term operation. HR‐TEM analysis revealed a CoOOH‐like oxyhydroxide phase on the surface of the vanadium‐exchanged Co(OH)_2_ electrode, indicating OER‐induced surface reconstruction (Figure S26). In the inner region, both CoOOH‐like oxyhydroxide and Co(OH)_2_ phases coexist, suggesting that the vanadium‐exchanged Co(OH)_2_ framework is partly retained after OER operation rather than being completely converted into a single CoOOH phase. ZIF‐67 also shows partial CoOOH‐like surface phases after OER operation, but the reconstructed region is much less developed than that observed in the vanadium‐exchanged Co(OH)_2_ electrode. This observation is consistent with post‐stability SAED patterns and in situ Raman spectra, which show more pronounced CoOOH‐related features for the vanadium‐exchanged Co(OH)_2_ electrode. Poststability XPS analysis reveals a negative shift in Co 2p and an increased Co^3+^ fraction, indicating changes in the local Co electronic environment and partial surface oxidation toward a CoOOH‐like oxyhydroxide phase (Figure S27). In addition, O 1s spectra show that the defect‐related oxygen features are largely retained after OER operation, suggesting that the oxygen vacancy‐rich environment introduced by vanadium cation exchange is not eliminated during operation (Table S8). These results support the formation of a CoOOH‐like reconstructed surface while preserving defect‐related electronic features relevant to enhanced OER kinetics [53, 59].

Nyquist plots measured in 1.0 M TMAOH electrolytes (Figure S28 and Table S9) provided additional insight into the reaction kinetics. With increasing vanadium incorporation, the charge‐transfer resistance exhibited a systematically larger increase upon changing from KOH to TMAOH, indicating that the OER kinetics became increasingly sensitive to the underlying reaction pathway. A distributed relaxation time (DRT) analysis was conducted to further examine the kinetic processes associated with lattice oxygen participation. The DRT spectra were divided into three characteristic frequency regions (Figure 4e and Figure S29). The high‐frequency region (R1, 10^2^–10^4 ^Hz) corresponds to the intrinsic electronic transport within the electrode, the mid‐frequency region (R2, 10^0^–10^2 ^Hz) represents the surface adsorption and desorption processes associated with evolution of oxygenated intermediates, and the low‐frequency region (R3, 10^−2^–10^0 ^Hz) is associated with slower pathway‐sensitive charge‐transfer processes related to lattice oxygen participation. A pronounced suppression of the R3 contribution was observed in 1.0 M TMAOH compared with 1.0 M KOH, indicating that the low‐frequency charge‐transfer process is strongly affected by the suppression of peroxo‐ and superoxo‐like intermediates associated with lattice oxygen‐related reaction steps. The extent of TMA^+^‐induced inhibition became more pronounced with increasing vanadium concentration, consistent with the increased contribution from lattice oxygen in the vanadium‐exchanged electrodes. In contrast, ZIF‐67 exhibited relatively smaller changes in the OER activity and reaction kinetics, supporting its relatively limited lattice oxygen participation.

The pH dependence of the OER activity was examined to differentiate between the AEM and LOM pathways. The current density of V_20_–Co(OH)_2_ at 1.55 V vs. RHE increased sharply with increasing pH (12.5–14.0), whereas ZIF‐67 exhibited a relatively weaker pH response (Figure S30). This pronounced pH dependence indicates strong pH‐dependent OER kinetics involving non‐concerted proton‐electron transfer behavior [56, 60, 61]. The extracted proton reaction order exhibited the trend of V_20_–Co(OH)_2_ (0.82) > V_15_–Co(OH)_2_ (0.77) > V_10_–Co(OH)_2_ (0.75) > ZIF‐67 (0.63) (Figure 4f). The higher ρ value of V_20_–Co(OH)_2_ suggests that vanadium cation exchange strengthens pathway‐sensitive OER kinetics, consistent with the mechanistic trend observed from the TMA^+^ and DRT analyses. The shift of the characteristic frequency toward the low‐frequency region with increasing vanadium concentration (Figure 3d and Figure S31) suggests an increased contribution from lattice oxygen‐related charge‐transfer processes. The sensitivity of individual kinetic components to pH was further examined using DRT analysis. Decreasing the pH led to an overall increase in resistance, with the most pronounced change occurring in R3 (Figure 4g and Figure S32). This observation indicates that the LOM‐related charge‐transfer processes are particularly sensitive to proton availability. Figure 4h shows the correlation of the oxygen vacancy concentration derived from the O 1s XPS analysis with the relative performance degradation in the TMAOH electrolyte and the proton reaction order. Vanadium cation exchange facilitates the formation of oxygen vacancies, accompanied by an increased LOM contribution. Combined with the kinetic correlations (Figure 3g), these results demonstrate that enhanced lattice oxygen participation is closely associated with improved OER activity. Therefore, the combined chemical probe experiments, DRT analysis, and pH‐dependent kinetics indicate that vanadium cation exchange effectively promotes lattice oxygen participation by increasing the oxygen vacancy concentration and modifying the local electronic structure, thereby accounting for the enhanced OER kinetics observed for V_20_–Co(OH)_2_.

To examine whether the design principles developed for oxygen electrocatalysis were applicable beyond the OER, the V_20_–Co(OH)_2_ electrode was evaluated in representative energy storage and conversion systems, including supercapacitors and zinc–air batteries.

**FIGURE 5:**
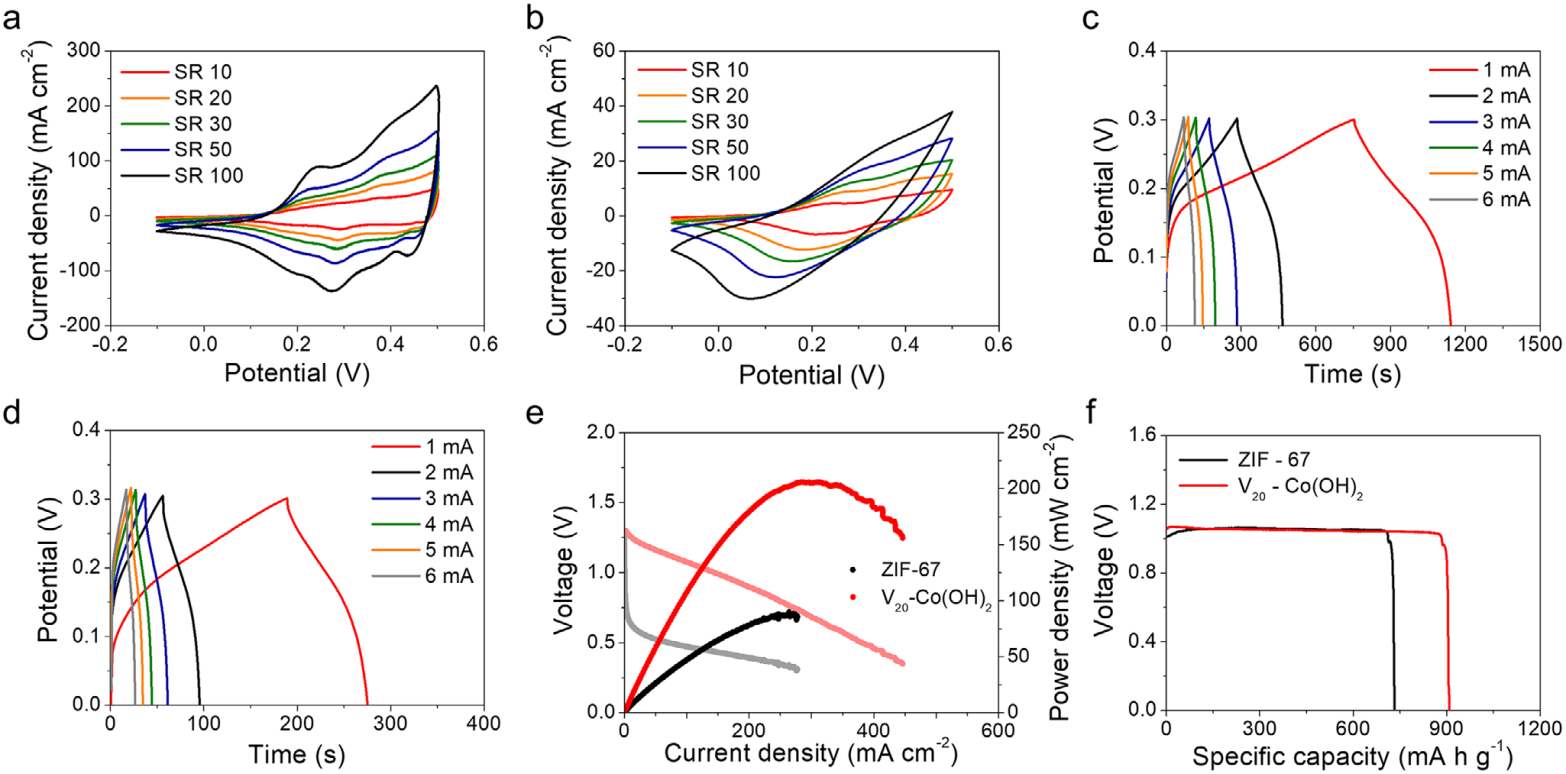
Extension of vanadium‐exchanged electrodes to energy storage and conversion systems. (a) CV curves of V_20_–Co(OH)_2_ at different scan rates. (b) CV curves of pristine ZIF‐67 at different scan rates. (c) GCD curves of V_20_–Co(OH)_2_ at current densities ranging from 1 to 6 mA cm^−2^. (d) GCD curves of pristine ZIF‐67 at current densities ranging from 1 to 6 mA cm^−2^. (e) Discharge polarization and power density curves of zinc–air batteries using pristine ZIF‐67 or V_20_–Co(OH)_2_ electrodes. (f) Specific capacitance of pristine ZIF‐67 and V_20_–Co(OH)_2_ at 5 mA cm^−2^.

The V_20_–Co(OH)_2_ electrode displayed a substantially larger integrated area in the CV curves than the pristine ZIF‐67 measured within the same potential window (Figure 5a,b), indicating a higher electrochemically accessible surface area. The broadened CV envelope of V_20_–Co(OH)_2_ can be attributed to the defect‐rich surface and mesoporous architecture induced by vanadium cation exchange, which facilitates ion transport within the electrode. The presence of distinct redox features in the CV profiles suggests that a pseudocapacitive charge storage process coexists with electric double‐layer capacitance, enabling additional charge storage through the reversible valence transitions of the cobalt species [62]. This behavior is consistent with electronically modulated and defect‐activated surfaces identified in OER electrocatalysis. Galvanostatic charge–discharge (GCD) measurements further demonstrated the favorable charge storage characteristics of the defect‐activated electrode. The discharge time of V_20_–Co(OH)_2_ exhibited no pronounced decay with increasing current density (Figure 5c), indicating a low internal resistance and homogeneous charge transport within the electrode. The pristine ZIF‐67 electrode exhibited a specific capacitance, energy density, and power density of 275.62 mF cm^−2^, 0.0036 mWh cm^−2^, and 0.305 mW cm^−2^, respectively. In contrast, the V_20_–Co(OH)_2_ electrode showed a markedly higher specific capacity of 1227.31 mF cm^−2^, an energy density of 0.015 mWh cm^−2^, and a power density of 0.301 mW cm^−2^, indicating the effectiveness of vanadium‐induced defect formation in enhancing charge storage kinetics. This behavior is consistent with the enhanced electronic conductivity observed during OER operation, suggesting that the conductive network formed by vanadium incorporation and oxygen vacancy formation remains effective under capacitive operating conditions. EIS provides additional insights into the charge transport process. The reduced semicircle in the Nyquist plot (Figure S33) corresponds to a lower charge‐transfer resistance (R_ct_), while the suppressed Warburg tail indicates a reduced ionic diffusion resistance. These results suggest that the defect‐rich architecture and charge redistribution induced by vanadium cation exchange effectively extend the charge transport pathways, enabling rapid ion migration and improved capacitive behavior.

To assess applicability in energy conversion devices, the V_20_–Co(OH)_2_ electrode was employed as the air electrode in a two‐electrode zinc–air battery using a mixed electrolyte of 6.0 M KOH and 0.2 M Zn(Ac)_2_. The V_20_–Co(OH)_2_‐based cell achieved a maximum power density of 205.7 mW cm^−2^, which is significantly higher than that of the ZIF‐67‐based cell (89.7 mW cm^−2^) (Figure 5e). This enhanced output performance originates from the accelerated charge‐transfer kinetics and improved oxygen adsorption/desorption capability identified in the OER electrocatalysis [63]. The charge–discharge polarization profiles (Figure 5e and Figure S34) revealed that the V_20_–Co(OH)_2_‐based zinc–air battery exhibited a smaller voltage gap than the ZIF‐67‐based battery, indicating improved round‐trip efficiency. In addition, the specific capacity of the V_20_–Co(OH)_2_ electrode reached 908 mAh g^−1^, surpassing that of the pristine ZIF‐67 electrode (733 mAh g^−1^) (Figure 5f). Long‐term cycling stability was evaluated at a constant current density of 5 mA cm^−2^. The V_20_–Co(OH)_2_‐based zinc–air battery maintained a nearly constant voltage profile without noticeable degradation over 50 h of operation (Figure S35), confirming its robust reversibility and structural integrity.

The V_20_–Co(OH)_2_ electrode demonstrated enhanced electrochemical performance in OER, supercapacitor, and zinc–air battery systems. Compared with previously reported supercapacitor and zinc–air battery electrodes, V_20_–Co(OH)_2_ exhibited competitive device‐level performance (Tables S10 and S11). These improvements are associated with the combined effects of the vanadium cation exchange‐induced modulation of metal‐site charge states, defect‐mediated enhancement of electrochemically accessible surface areas, and enhanced charge‐transport kinetics at the electrode–electrolyte interface. These results demonstrate that the strategy proposed in this study to engineer defects and electronic structures provides a generalizable and multifunctional electrode platform for high‐performance energy storage and conversion devices, rather than offering a reaction‐specific optimization. In addition, comparison with reported low‐/room‐temperature synthetic strategies and cation exchange‐based OER catalysts highlights that the present room‐temperature cation exchange approach provides an effective route for modulating the structural and electronic environments of electrocatalysts under mild synthetic conditions (Table S5).

## 3 Conclusions

In this study, the physical and electronic structures of MOF‐derived catalysts were systematically modulated using high‐valence metal cation exchange to enhance OER kinetics. Vanadium cation exchange enabled the room‐temperature removal of organic ligands from ZIF‐67 and the phase transformation into a mesoporous cobalt hydroxide framework, thereby avoiding the high‐temperature treatments typically required for MOF‐derived catalyst synthesis. The incorporation of high‐valence vanadium effectively tuned the electronic structure by increasing the cobalt oxidation state and inducing oxygen vacancy formation, thereby facilitating lattice oxygen participation during the OER. Consequently, the V_20_–Co(OH)_2_ electrode achieved current densities of 50 and 100 mA cm^−2^ at overpotentials of 268 and 293 mV, respectively, in a 1.0 M KOH electrolyte, while maintaining stable operation for over 150 h. Mechanistic analyses clarified the correlation between lattice oxygen participation, interfacial charge‐transfer characteristics, and electrochemical performance. In situ Raman and post‐stability analyses further revealed OER‐indueced formation of a CoOOH‐like reconstructed surface, while defect‐related oxygen features and a high Co oxidation state were largely retained after operation. Furthermore, the defect‐activated electrode exhibited enhanced performance in supercapacitors and zinc–air batteries, demonstrating the applicability of this design strategy. These findings provide a rational framework for designing high‐performance electrocatalysts via coupled structural modulation and defect engineering for various energy conversion and storage systems.

## 4 Materials and Methods

### 4.1 Materials and Chemicals

Nickel foam (thickness: 0.5 mm) was purchased from MTI Korea. Potassium hydroxide (KOH) was purchased from Daejung Chemicals. Cobalt chloride, sodium metavanadate, and nitric acid were purchased from Sigma‐Aldrich. 2‐Methylimidazole was purchased from Wako Chemicals. All chemicals were of analytical grade and were used as received without further purification.

### 4.2 Synthesis of ZIF‐67

The nickel foam was first immersed in diluted nitric acid to completely remove the native oxide layer. Cobalt chloride was dissolved in deionized (DI) water and stirred continuously until complete dissolution. Simultaneously, 2‐methylimidazole was dissolved in DI water under stirring. The two solutions were thoroughly mixed to form a homogeneous precursor solution. The oxide‐free nickel foam was immersed in the mixed solution, which was subsequently transferred to a Teflon‐lined autoclave and maintained at 120°C for 4 h. After hydrothermal synthesis, the obtained purple electrode was washed several times with DI water and ethanol, followed by overnight drying in a drying oven.

### 4.3 Synthesis of V_x_‐Co(OH)_2_

ZIF‐67 was prepared following a previously described procedure. Sodium metavanadate powder was gradually dissolved in DI water to prepare a vanadium precursor solution. The as‐synthesized purple ZIF‐67 electrode was immersed in the solution at a concentration within its solubility limit and maintained at room temperature while stirring with a magnetic bar for a predetermined time. The vanadium doping concentration and reaction time varied depending on the desired doping level. Specifically, V_10_–Co(OH)_2_ was prepared by immersing the electrode in a solution with a Co:V molar ratio of 60:1 for 1 h. V_15_–Co(OH)_2_ was obtained using a Co:V molar ratio of 30:1 for 1 h. V_20_–Co(OH)_2_ was prepared with a Co:V molar ratio of 15:1 for 3 h, while V_25_‐Co(OH)_2_ was synthesized using a Co:V molar ratio of 10:1 for 3 h.

### 4.4 Characterization

SEM (JEOL Ltd., JSM‐IT800) and HRTEM (JEOL Ltd., JEM‐F200) were employed to examine the electrode morphology and structural features. XRD (Bruker D8 Advance) using Cu Kα radiation (λ = 1.5406 Å) was conducted at room temperature to analyze the phase composition and crystallinity. The elemental compositions and atomic ratios were determined using ICP‐MS (Nexlon 2000). TGA (STA7200RV) was performed to evaluate the removal of organic ligands. Raman spectroscopy (XperRam35V, Nanobase) was used to analyze the chemical characteristics of the samples. The specific surface areas of the electrodes were measured using the BET (Micromeritics TRISTAR 3020) instrument. XPS (ESCA Lab 250, VG Scientific Instruments) was used to investigate the chemical states and electronic properties of the electrodes. The Co K‐edge and V K‐edge XAS spectra were collected using R‐XAFS. All the XAS spectra were acquired by averaging at least three consecutive scans performed under identical experimental conditions. Data normalization was performed using the Athena software, which involved pre‐edge background subtraction and post‐edge normalization. EXAFS fitting and determination of the coordination numbers for the Co and V metal centers were carried out using the Artemis software. In situ Raman spectroscopy (LabRAM Aramis, Horiba Jobin Yvon) was used to analyze the reaction behavior.

### 4.5 Electrochemical Measurements for OER

All electrochemical measurements were conducted at room temperature in a conventional three‐electrode configuration using a 1.0 M KOH electrolyte and a Gamry Interface 1010 potentiostat. Catalyst‐coated nickel foam (1 × 1 cm^2^) was used as the working electrode and was electrically connected via a Pt holder. A Hg/HgO electrode and a Pt mesh served as the reference and counter electrodes, respectively. All potentials were converted to the reversible hydrogen electrode (RHE) scale as follows:

$$E_{RHE}=E_{Hg/HgO}+0.241+0.059\times pH$$

LSV was performed in the potential range of 1.0–1.8 V vs. RHE in 1.0 M KOH. All the LSV curves were corrected using iR compensation to eliminate solution‐resistance effects. The Tafel slopes were derived from the polarization curves by fitting the linear region near the onset potential in the plots of the overpotential vs. the logarithm of the current density (log|j|). The C_dl_ values of the prepared electrodes were calculated using CV in the non‐faradaic region at various scan rates (from 20 to 100 mV s^−1^) in a 1.0 M KOH solution. EIS was used to evaluate the charge‐transfer kinetics. It was performed at frequencies ranging from 0.1 to 100 000 Hz under an AC voltage of 5 mV. Long‐term durability and stability were assessed by chronopotentiometry measurements at a current density of 100 mA cm^−^^2^ for 150 h to investigate their effect on long‐term OER performance.

### 4.6 Supercapacitor Measurements

The electrochemical measurements of the supercapacitors were performed using the same device and OER conditions. All capacitive performance parameters were normalized to the geometric area of the electrode. The areal specific capacitance (C_a_, mF cm^−2^), areal energy density (E_A_, mWh cm^−2^), and power density (P_A_, mW cm^−2^) of the supercapacitor electrode were calculated as follows:

$$C_{A}=\frac{I\Delta t}{A\Delta V}$$

$$E_{A}=\frac{C_{A}\Delta V^{2}}{2\times 3.6}$$

$$P_{A}=\frac{3600\times E_{A}}{\Delta t}$$

where *I* is the discharge current (A), Δ*V* is the potential window (V), Δ*t* is the discharge time, and *A* is the electrode area.

### 4.7 Zinc–Air Battery Measurements

Electrochemical measurements of the zinc–air battery were performed using the same device used for the OER. A Zn‐V_20_–Co(OH)_2_ battery was assembled using V_20_–Co(OH)_2_ and Zn foil as the positive and negative electrodes, respectively, with a polished Zn plate (0.5 mm thickness). An alkaline solution of 6 M KOH + 0.2 M Zn(OAc)_2_ was used as the electrolyte. A bare Ni foam was used as the current collector at the anode. The air cathode has an area of 1 cm^2^, allowing oxygen from nearby air to reach the gas diffusion layer. The power density and specific capacity were calculated as follows:

Powerdensity=I×V

$$\mathrm{Specific}\;\mathrm{capacity}=\frac{It}{weight\;of\;consumed\;Zn}$$

where *I* is the current density (mA cm^−2^), V is the voltage, and *t* is the operating time.

## Author Contributions

**Wonyoung Lee**: supervision, Writing – review and editing, conceptualization. **Seungwoo Han**: conceptualization, methodology, software, data curation, formal analysis, writing – original draft, validation. **Yongbeen Kim**: conceptualization, methodology, software, data curation, formal analysis, writing – original draft, validation.

## Conflicts of Interest

The authors declare no conflicts of interest.

## Supporting information




**Supporting File**: advs76610‐sup‐0001‐SuppMat.pdf.

## Data Availability

The data that support the findings of this study are available from the corresponding author upon reasonable request.
