# Supplementary material for: Vanadium Cation Exchange‐Driven Reconstruction of MOF‐Derived Cobalt Hydroxide Electrodes for Electrocatalysis and Energy Storage
Source: Adv Sci (Weinh). 2026 Jul 14;13(56):e76610. doi: 10.1002/advs.76610 (PMC13367113; doi:10.1002/advs.76610)
Supplement: Supplementary file 1 [file ADVS-13-e76610-s001.pdf]

Supplementary Information

Vanadium Cation Exchange-Driven Reconstruction of MOF-Derived Cobalt Hydroxide Electrodes for Electrocatalysis and Energy Storage

*Yongbeen Kim †, Seungwoo Han †, and Wonyoung Lee**

Y. Kim, S. Han, W. Lee

School of Mechanical Engineering, Sungkyunkwan University (SKKU), Suwon 16419, South Korea

*W. Lee

SKKU Institute of Energy Science and Technology (SIEST), Sungkyunkwan University, Suwon, Gyeonggi-do 16419, Republic of Korea

E-mail: leewy@skku.edu

†: These authors equally contributed to this work

Table S1. ICP-MS of vanadium-exchanged electrocatalysts.

Catalyst	Element	Final concentration (mg/kg)	Final concentration (at%)
V_{10}–$Co(OH)_2$	Co	124304.60	0.89
	V	13493.69	0.11
V_{15}–$Co(OH)_2$	Co	91659.21	0.83
	V	16060.67	0.17
V_{20}–$Co(OH)_2$	Co	57919.39	0.78
	V	14090.30	0.22
V_{25}–$Co(OH)_2$	Co	75299.34	0.76
	V	21110.73	0.24

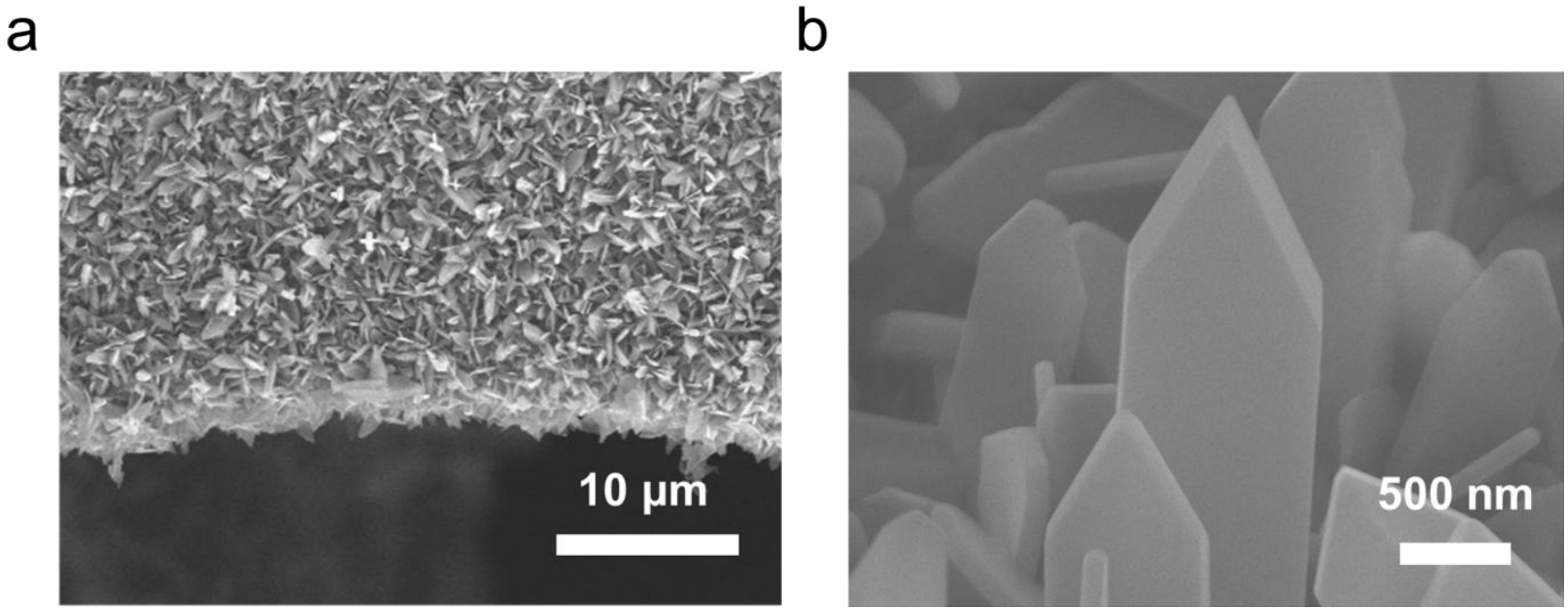

Figure S1. SEM images of pristine ZIF-67 at different scales.

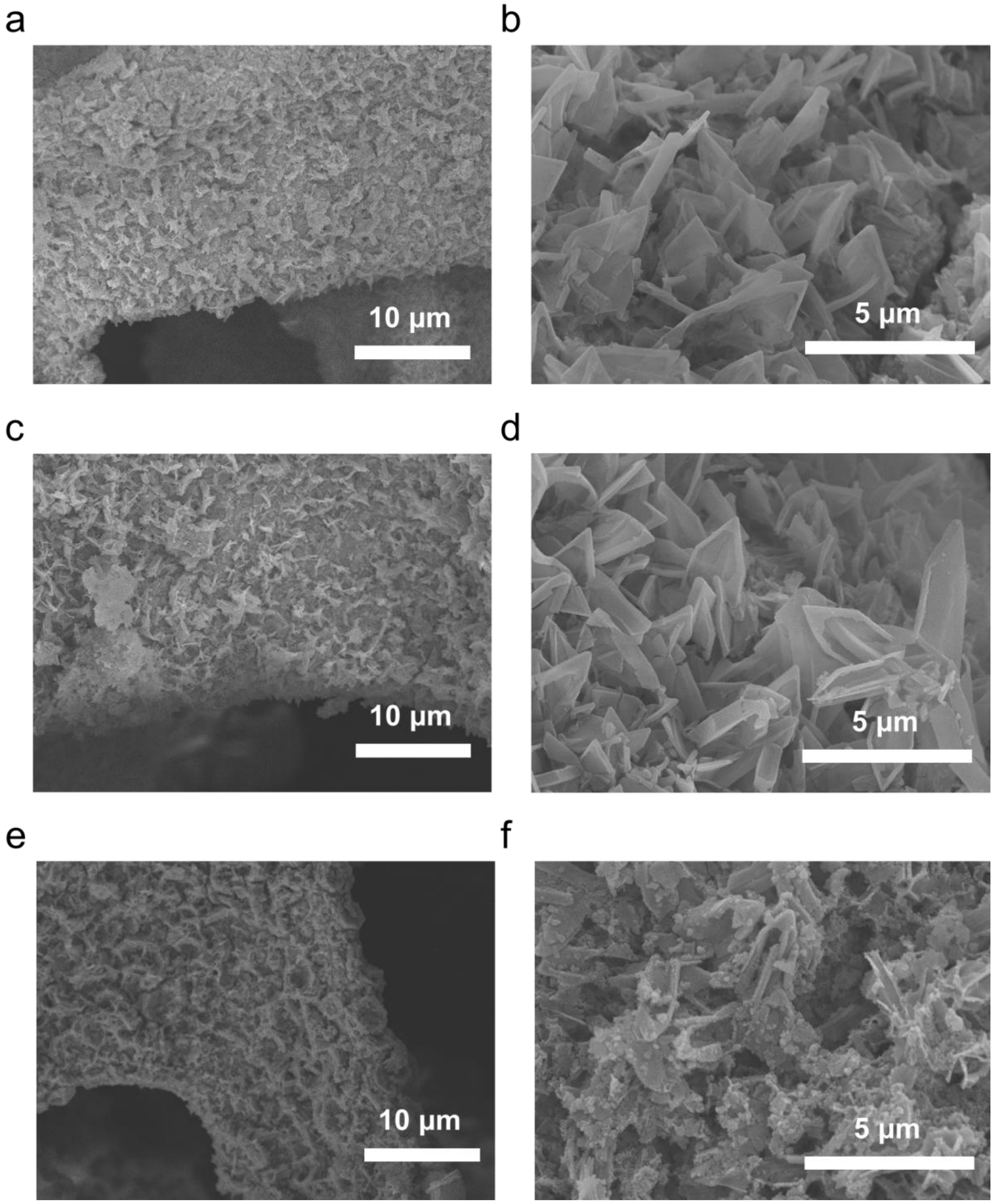

Figure S2. SEM images of (a–b) V_{10}–$Co(OH)_2$, (c–d) V_{15}–$Co(OH)_2$, and (e–f) V_{25}–$Co(OH)_2$ after vanadium cation exchange in pristine ZIF-67.

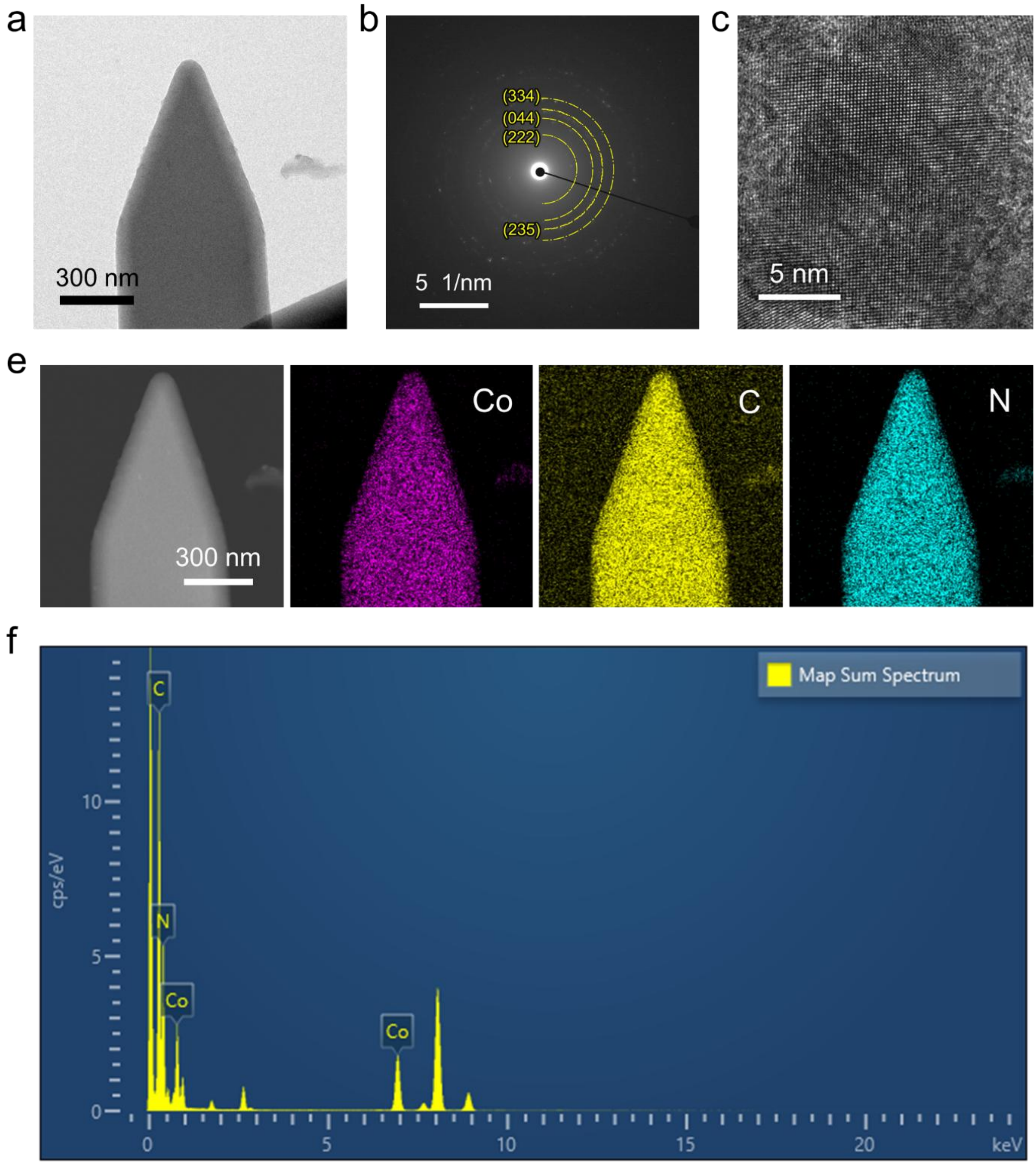

Figure S3. Structural characterization of pristine ZIF-67. **a** TEM image. **b** SAED pattern. **c** HRTEM image. **d** STEM EDS mapping images and **f** elemental contents.

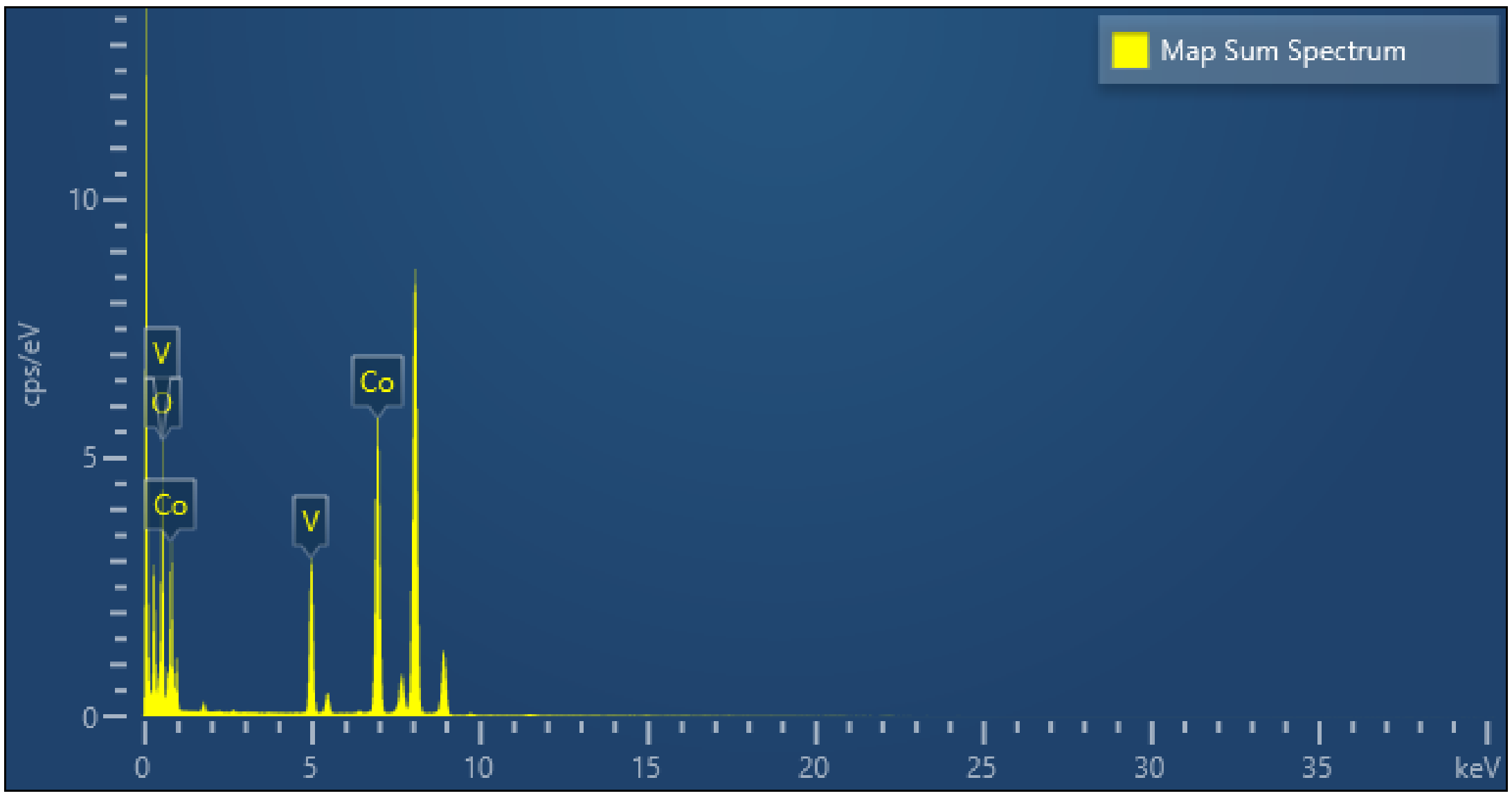

Figure S4. STEM EDS mapping element contents in V_{20}–$Co(OH)_2$.

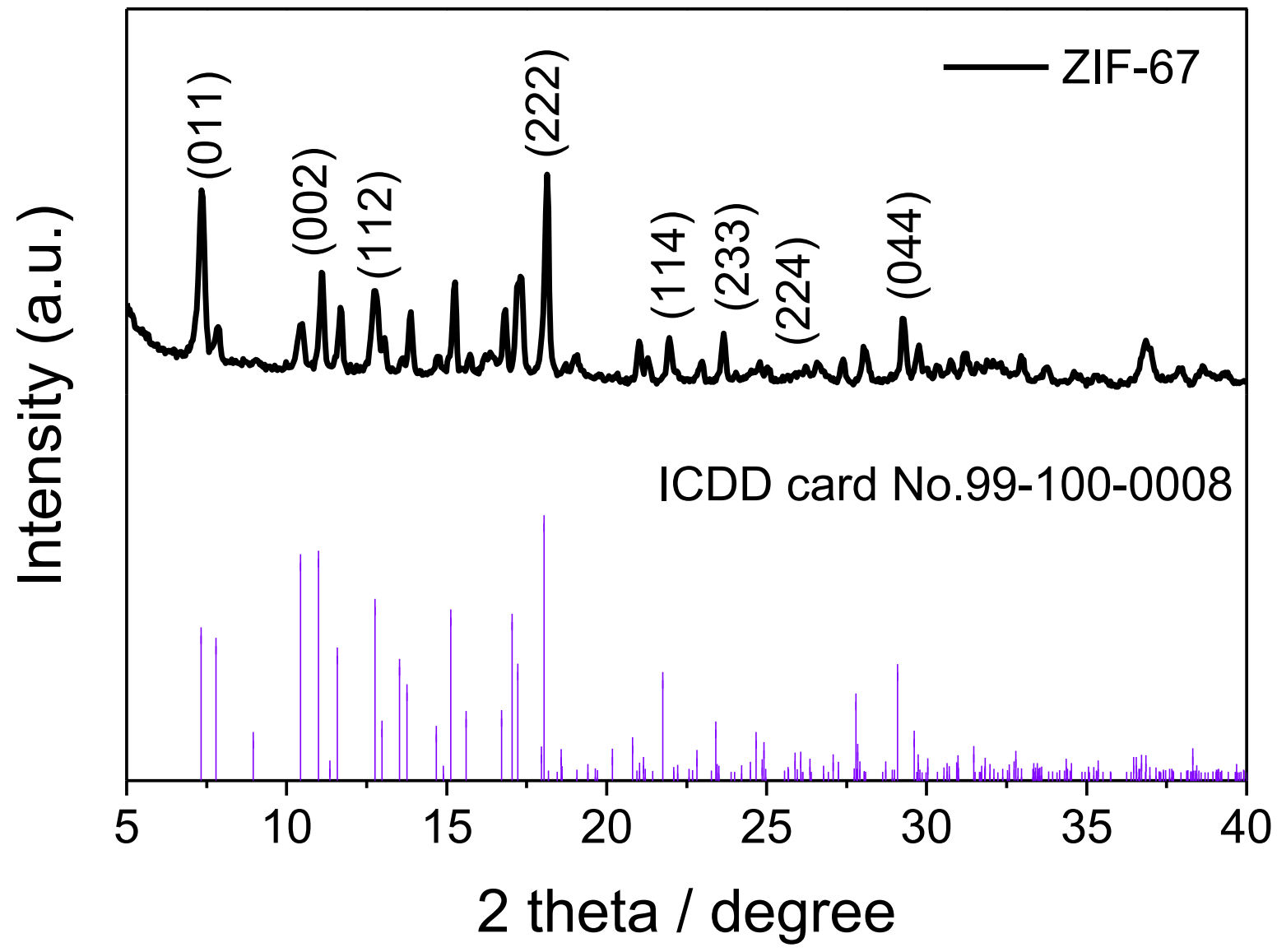

Figure S5. XRD pattern of pristine ZIF-67.

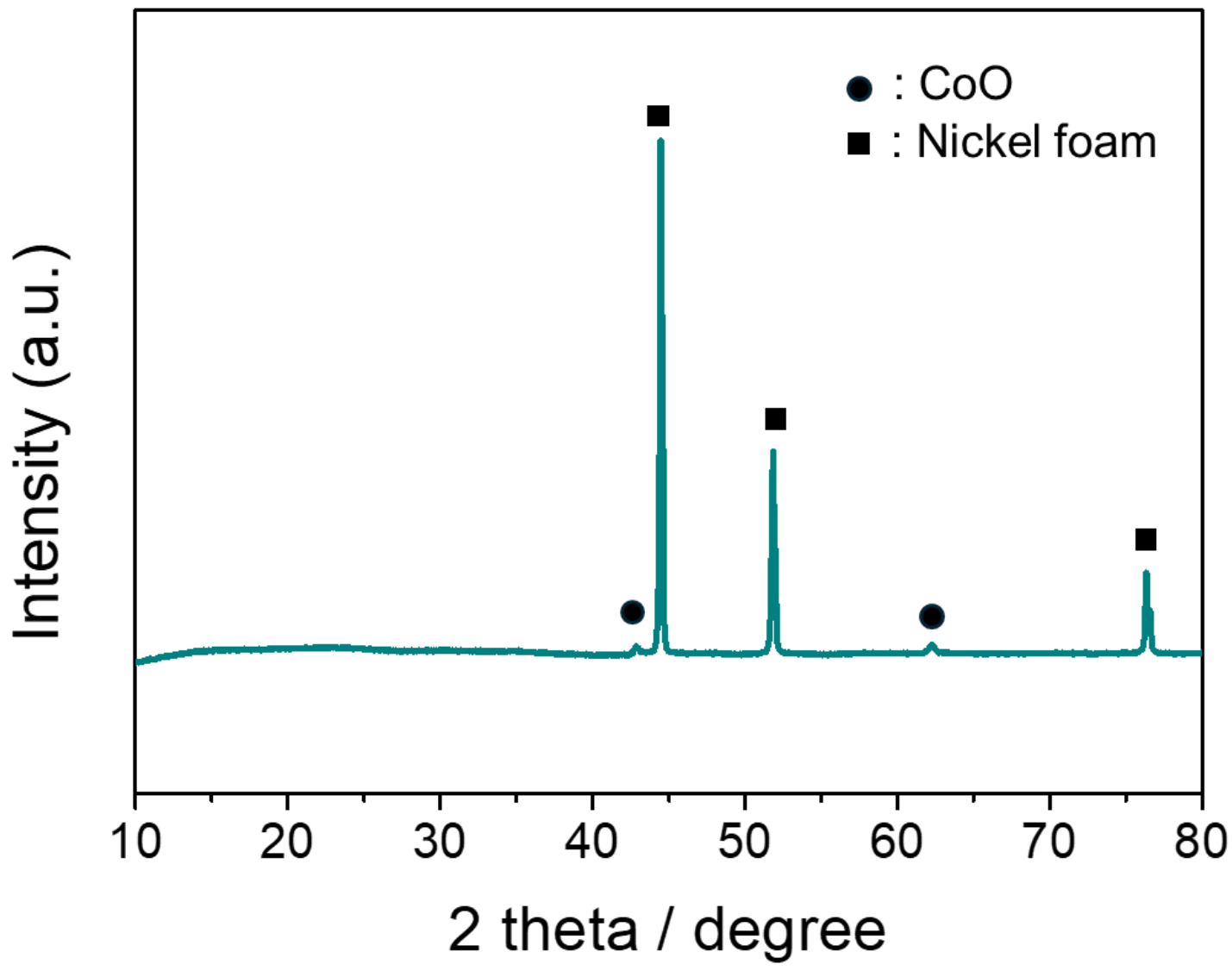

Figure S6. XRD pattern of V_{25}–$Co(OH)_2$ containing an excessive amount of vanadium. The diffraction peaks indicate the formation of Co oxide phase.

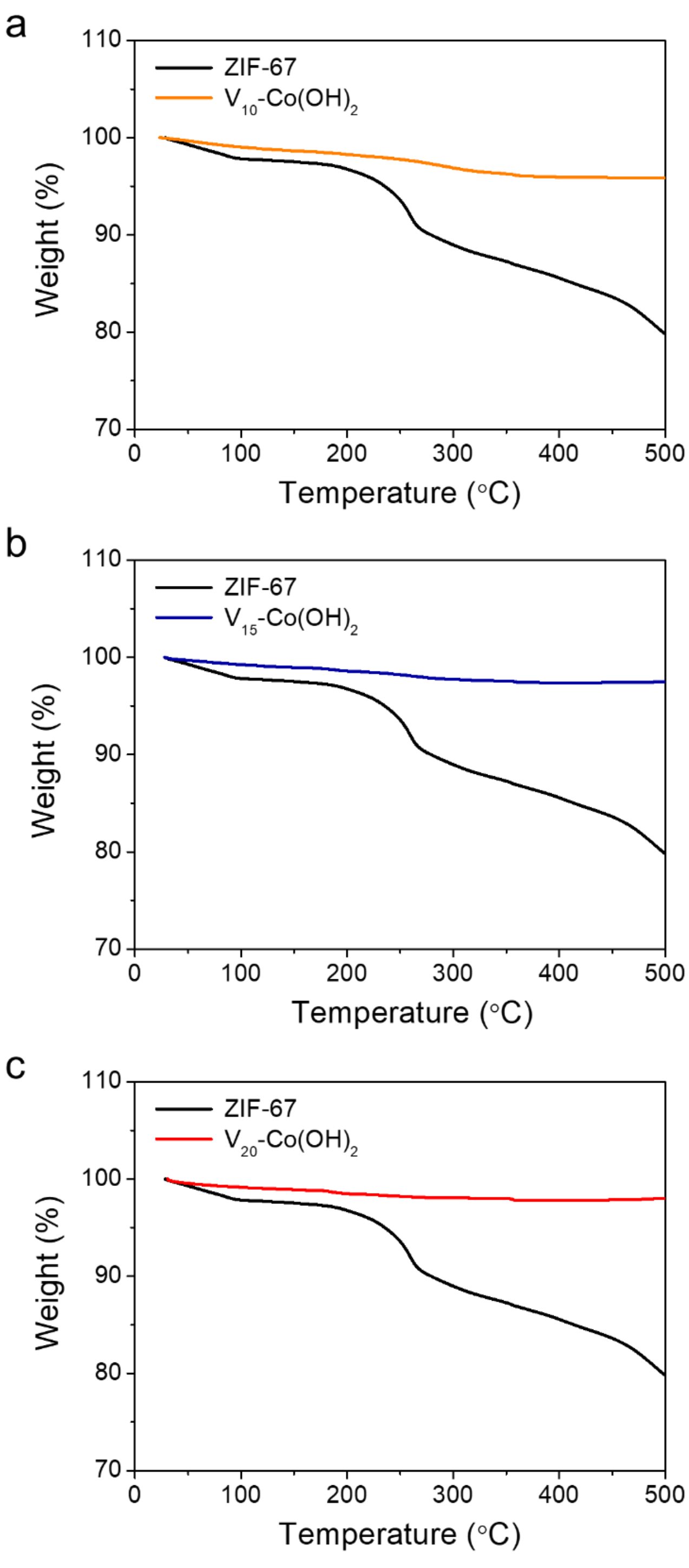

Figure S7. TGA profiles of **a** V_{10}–$Co(OH)_2$, **b** V_{15}–$Co(OH)_2$, and **c** V_{20}–$Co(OH)_2$.The diminished mass loss associated with ligand removal confirms the reduction of organic ligand after vanadium incorporation.

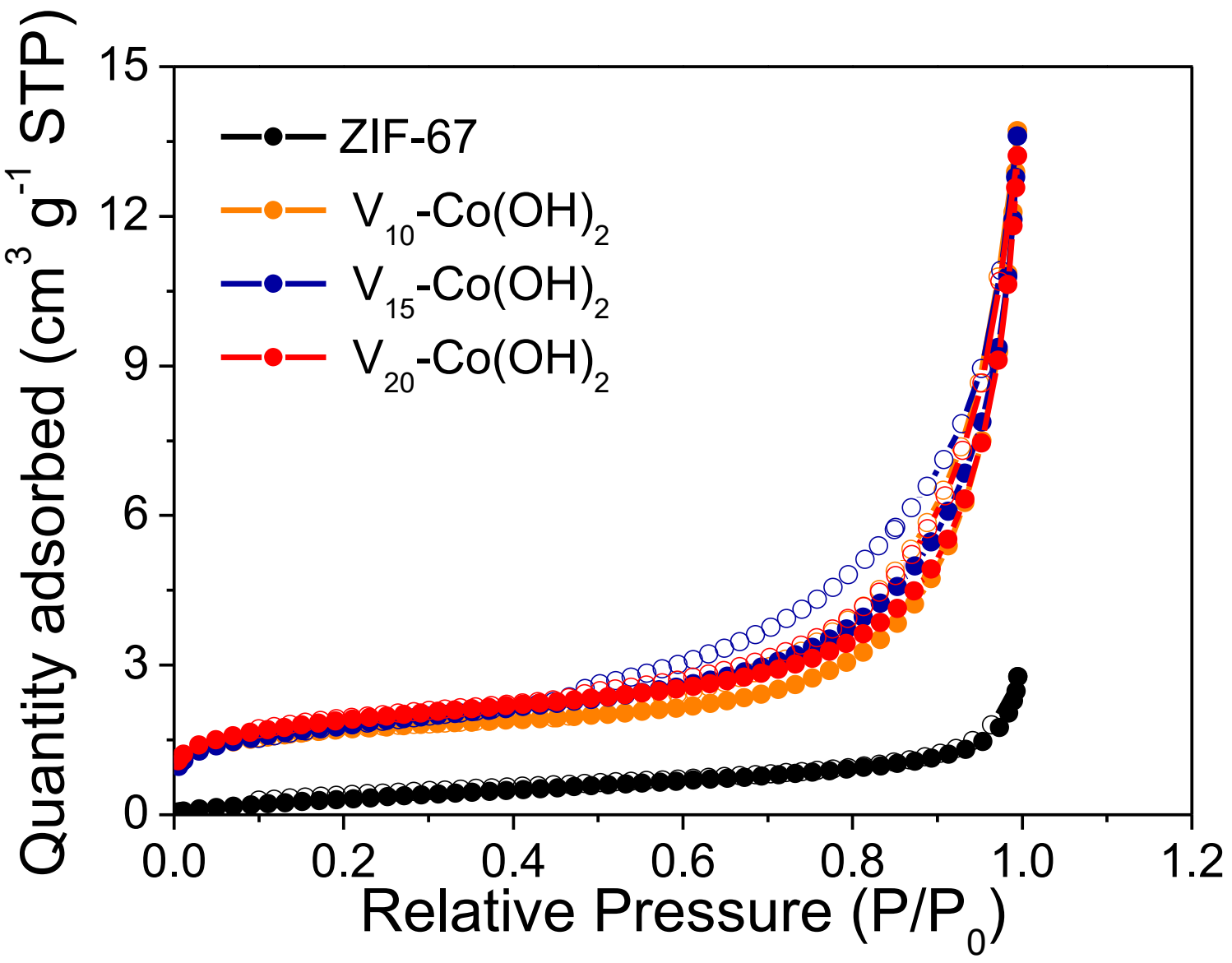

Figure S8. BET analysis of pristine ZIF-67, V_{10}–$Co(OH)_2$, V_{15}–$Co(OH)_2$, and V_{20}–$Co(OH)_2$.

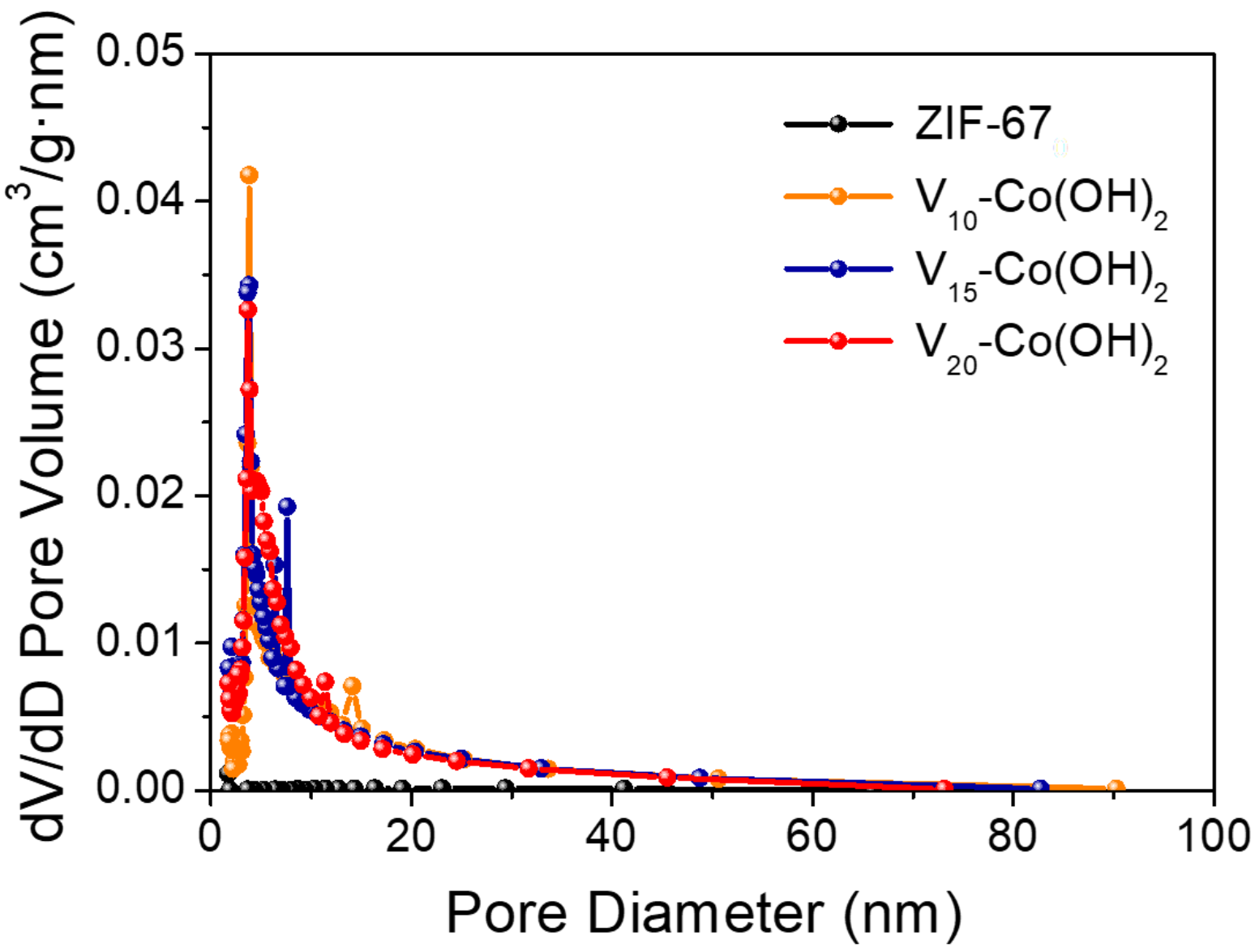

Figure S9. Barett–Joyner–Halenda plot of pristine ZIF-67, V_{10}–$Co(OH)_2$, V_{15}–$Co(OH)_2$, and V_{20}–$Co(OH)_2$.

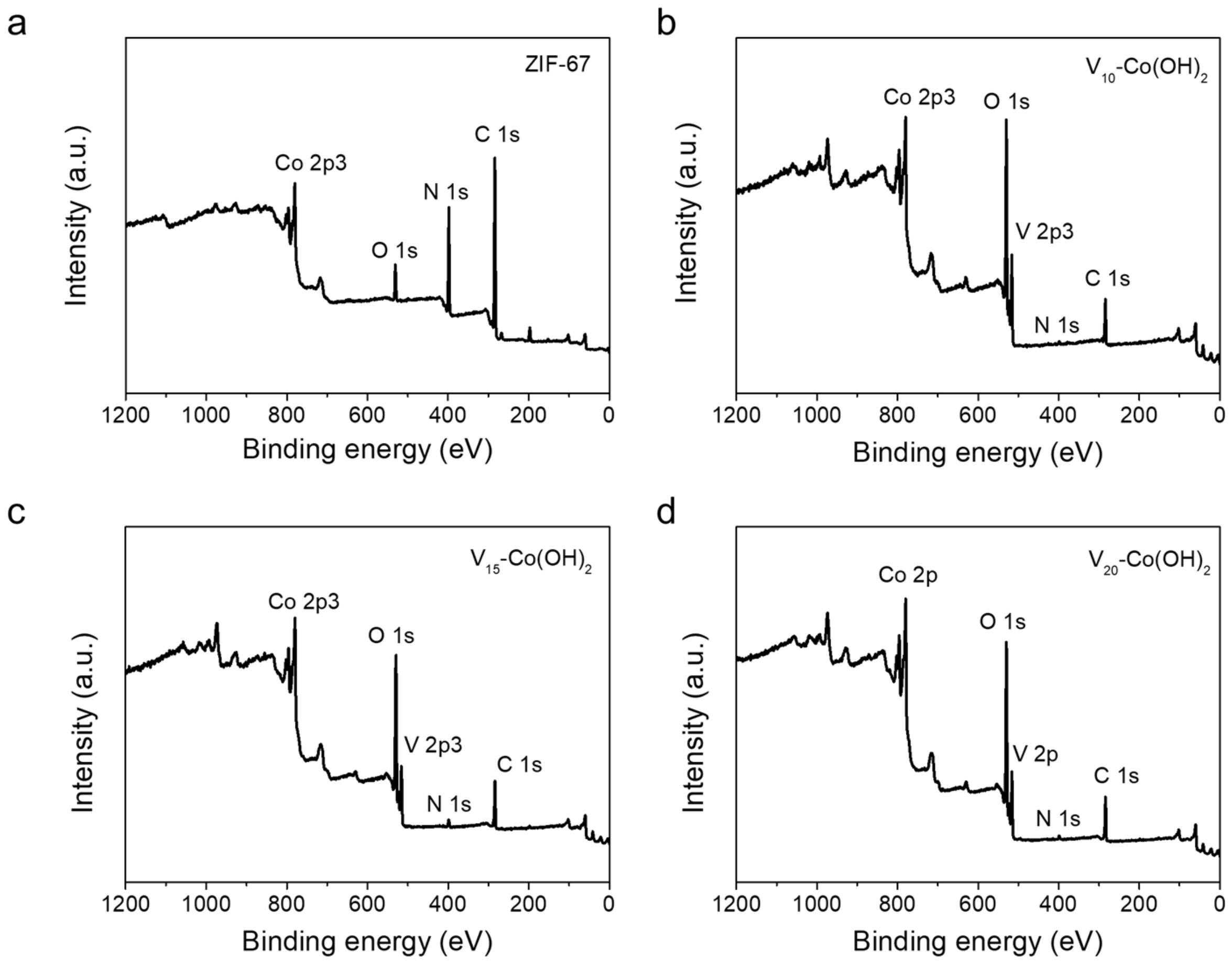

Figure S10. XPS survey spectra of **a** pristine ZIF-67, **b** V_{10}–$Co(OH)_2$, **c** V_{15}–$Co(OH)_2$, and **d** V_{20}–$Co(OH)_2$.

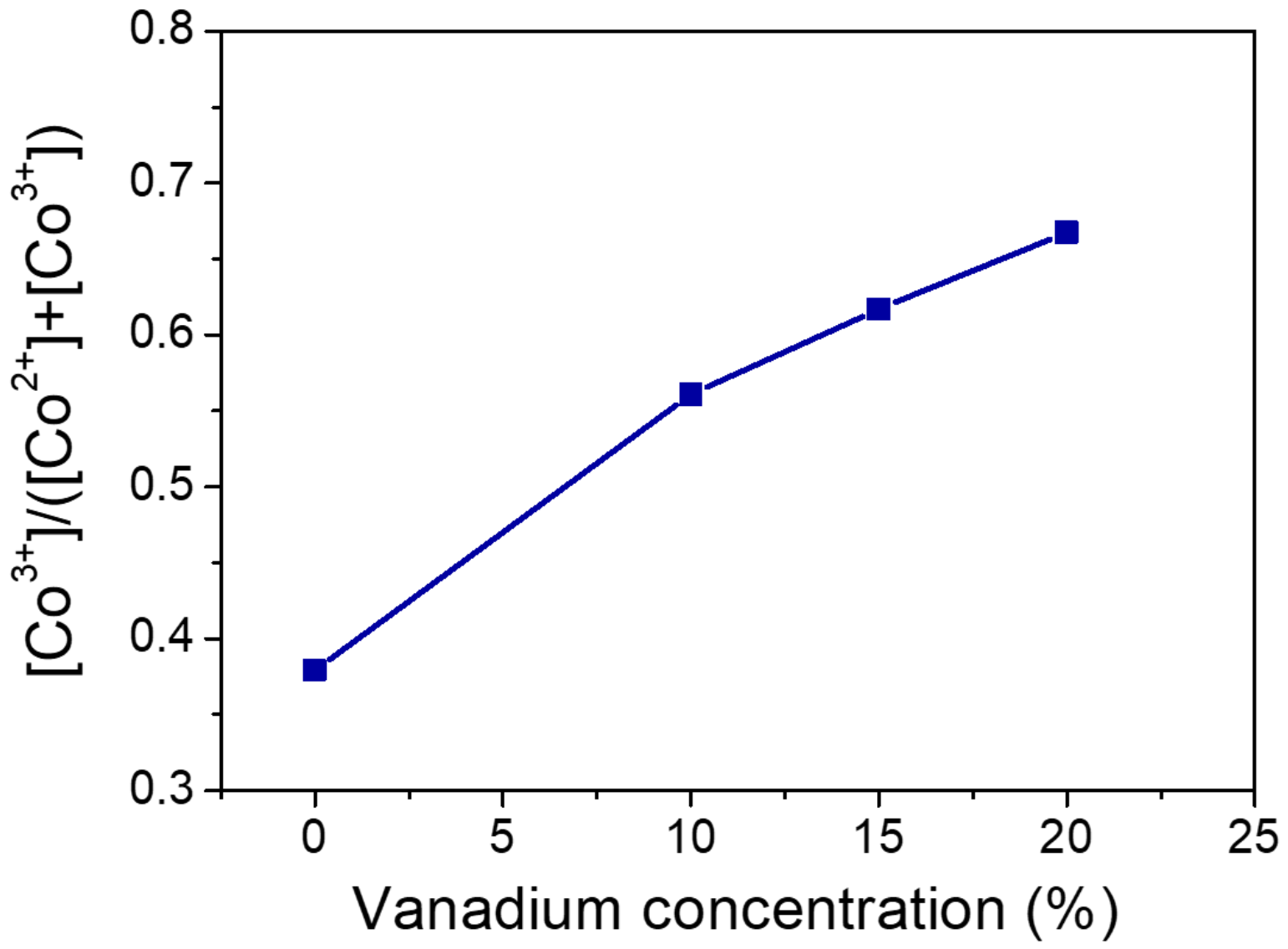

Figure S11. Co^{3+} concentration as a function of the relative fraction of vanadium concentration.

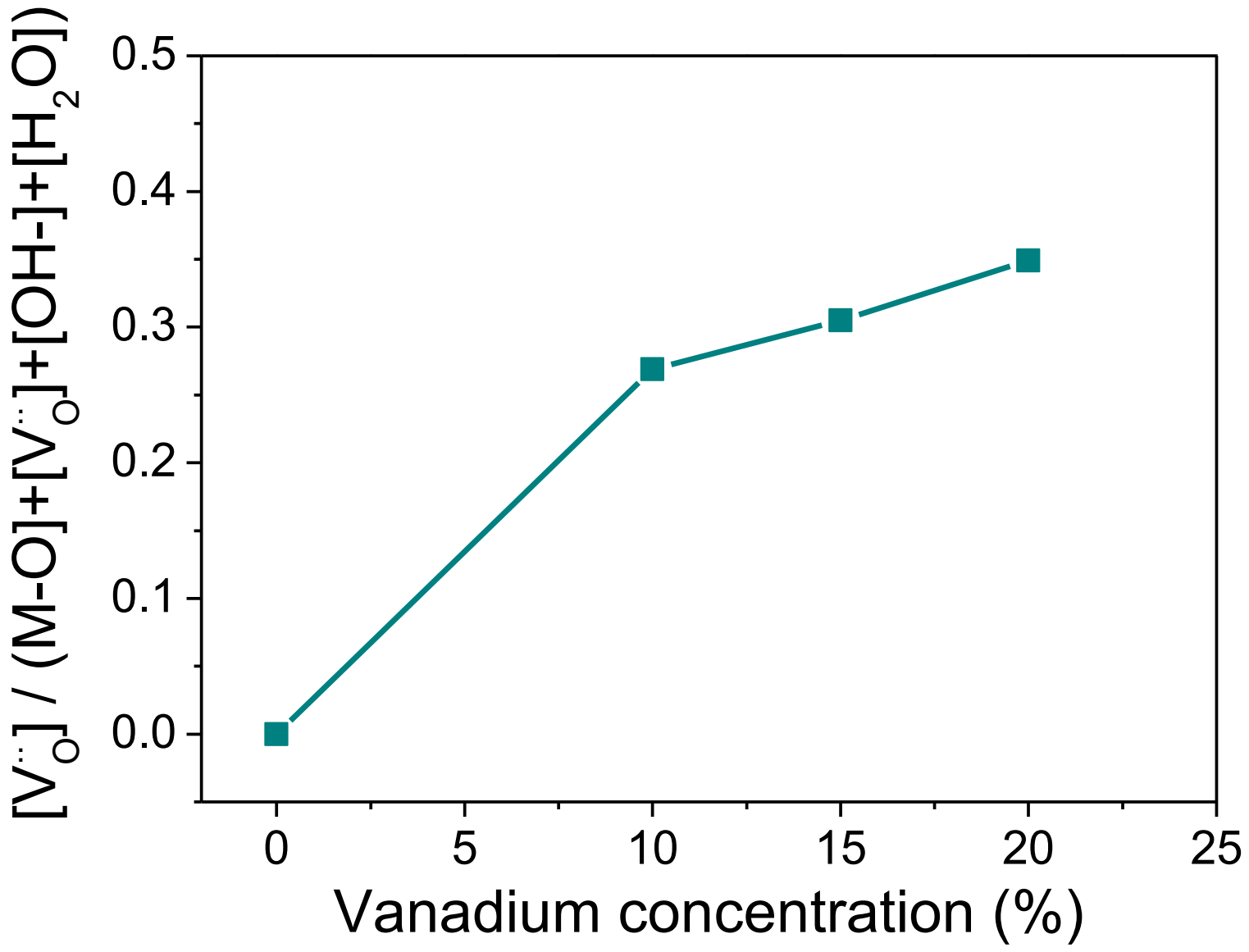

Figure S12. Oxygen vacancy concentration as a function of the relative fraction of vanadium concentration.

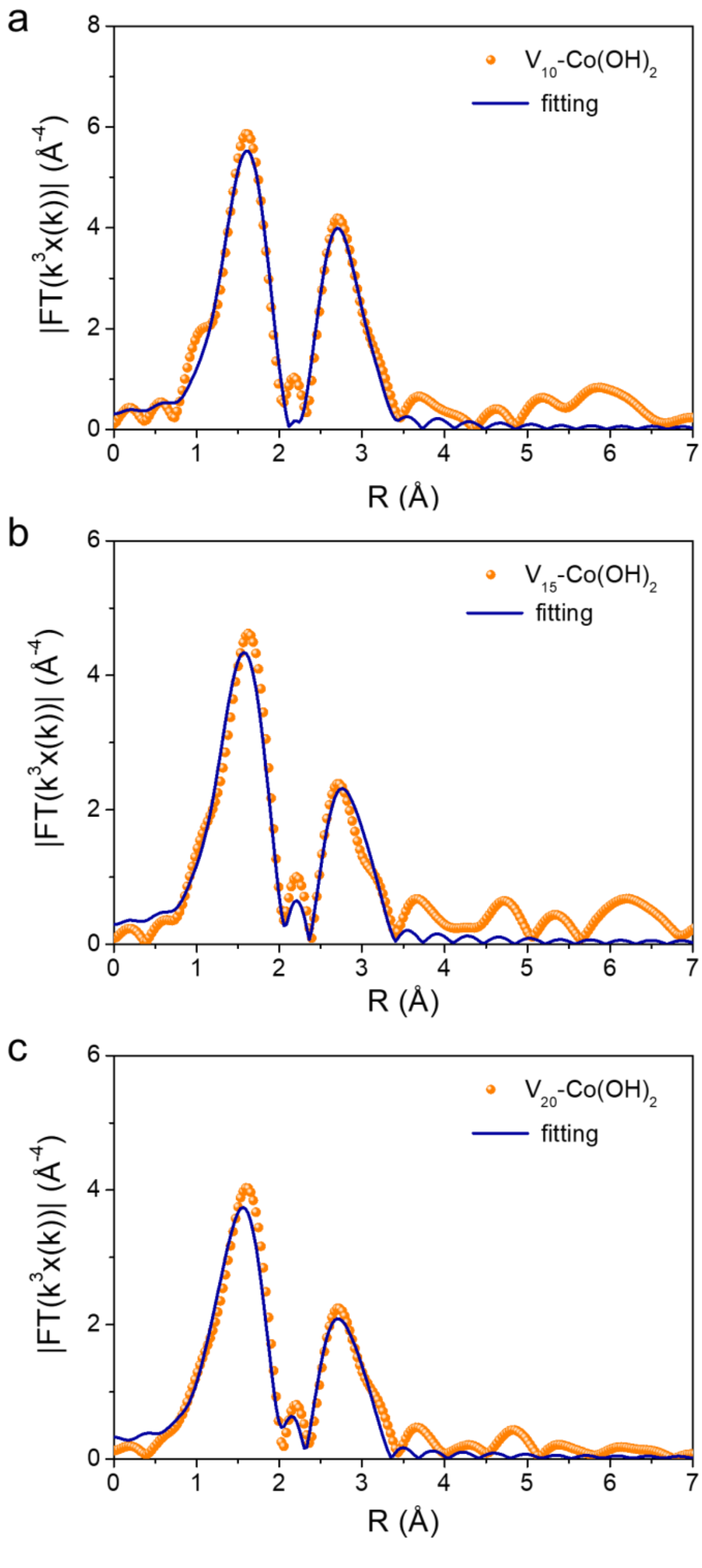

Figure S13. Co K-edge EXAFS fitting analyses of **a** V_{10}–Co(OH)$_2$, **b** V_{15}–Co(OH)$_2$, and **c** V_{20}–Co(OH)$_2$ in R space.

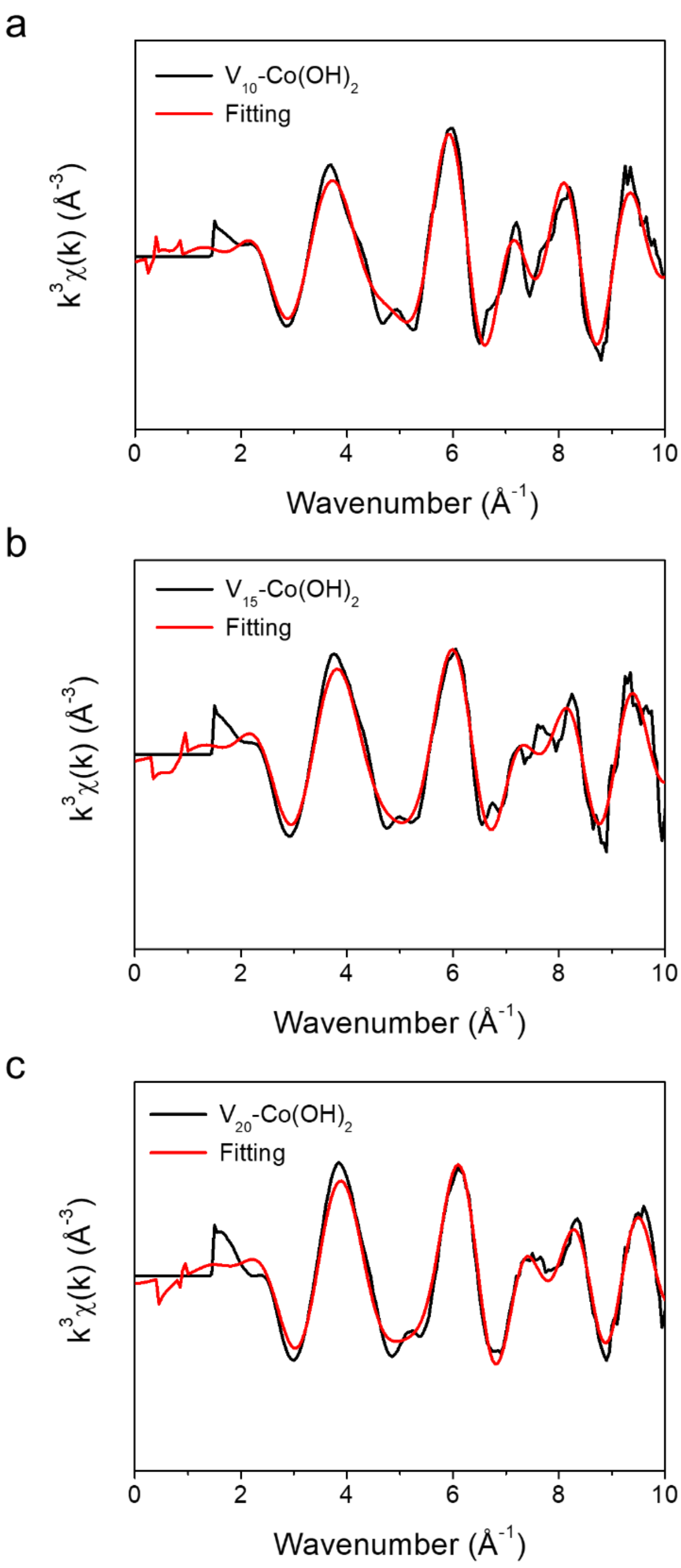

Figure S14. Co K-edge FT-EXAFS fitting analyses of **a** V_{10}–$Co(OH)_2$, **b** V_{15}–$Co(OH)_2$, and **c** V_{20}–$Co(OH)_2$ in *k* space.

Table S2. The curve fitting for the V_{10}–$Co(OH)_2$, V_{15}–$Co(OH)_2$, and V_{20}–$Co(OH)_2$ electrocatalysts was performed on the k^3-weighted EXAFS function χ(k) data in the R-range of 1.0–3.6 Å. All the R-factors for the fitted results are within 0.02, indicating the goodness of the fit.

Name	Path	N	σ^2 (10^{-3})	R (Å)	R-factor (10^{-1})
V_{10}–$Co(OH)_2$	Co–O	5.492	10.95	2.096	0.023
	Co–Co	4.083	9.42	3.160	
	Co–V	0.924	2.00	3.429	
V_{15}–$Co(OH)_2$	Co–O	5.182	13.84	2.077	0.029
	Co–Co	3.698	11.00	3.134	
	Co–V	1.097	10.48	2.952	
V_{20}–$Co(OH)_2$	Co–O	4.912	15.61	2.057	0.040
	Co–Co	3.411	11.6	3.088	
	Co–V	1.381	14.16	2.911	

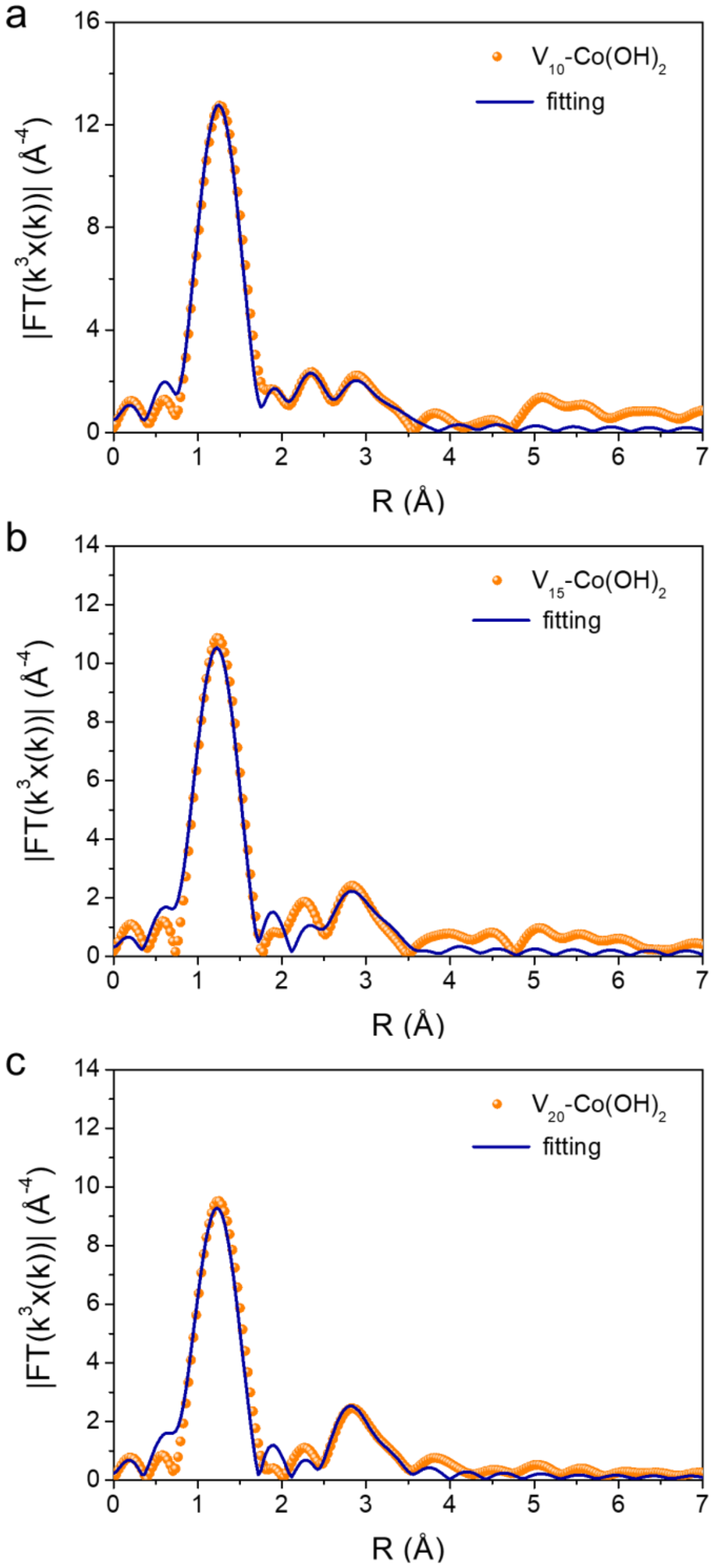

Figure S15. V K-edge EXAFS fitting analyses of **a** V_{10}–$Co(OH)_2$, **b** V_{15}–$Co(OH)_2$, and **c** V_{20}–$Co(OH)_2$ in R space.

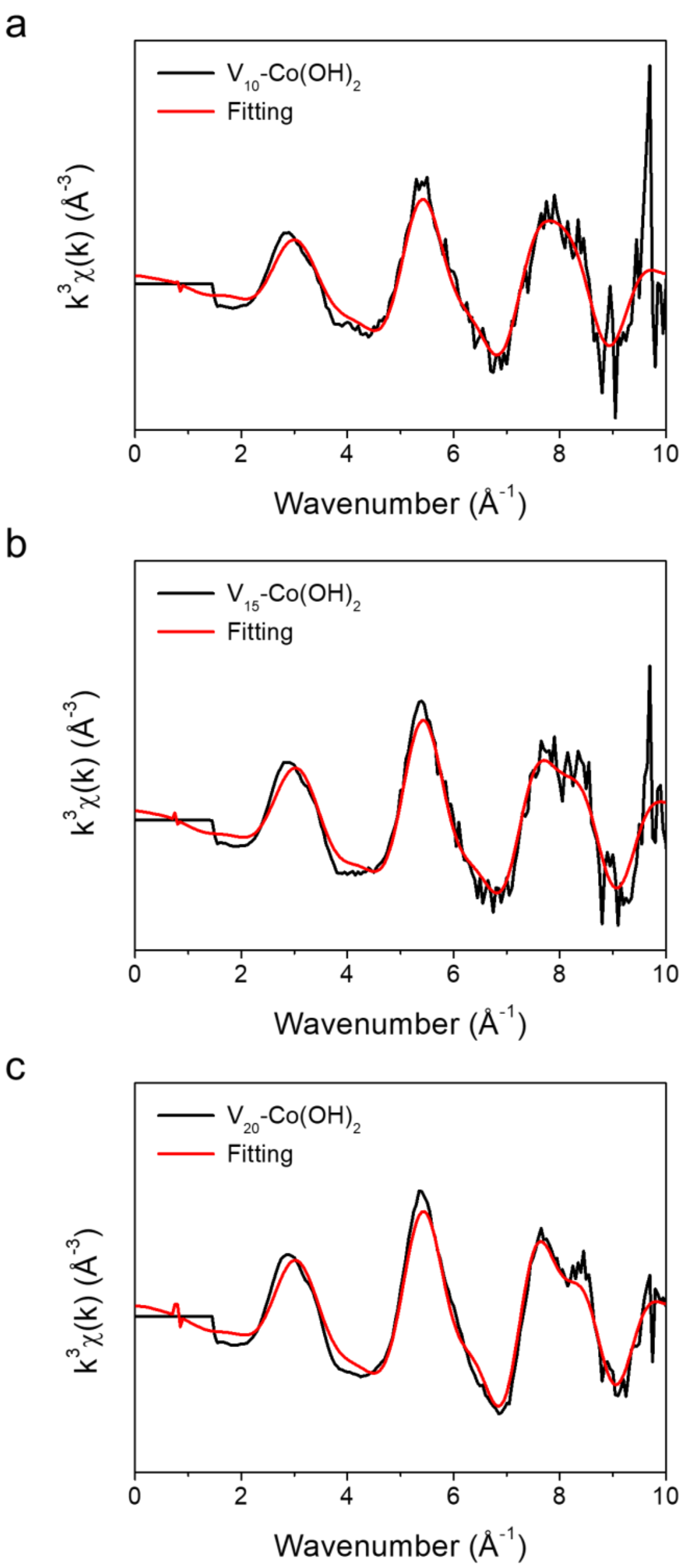

Figure S16. V K-edge FT-EXAFS fitting analyses of **a** V_{10}–$Co(OH)_2$, **b** V_{15}–$Co(OH)_2$, and **c** V_{20}–$Co(OH)_2$ in *k* space.

Table S3. The curve fitting for the V_{10}–$Co(OH)_2$, V_{15}–$Co(OH)_2$, and V_{20}–$Co(OH)_2$ electrocatalysts was performed on the k^3-weighted EXAFS function χ(k) data in the R-range of 1.0–3.6 Å. All the R-factors for the fitted results are within 0.02, indicating the goodness of the fit.

Name	Path	N	σ^2 (10^{-3})	R (Å)	R-factor (10^{-1})
V_{10}–$Co(OH)_2$	V–O	4.274	2.32	1.708	0.009
	V–Co	3.948	12.89	3.499	
	V–V	2.273	13.72	2.909	
V_{15}–$Co(OH)_2$	V–O	4.209	4.27	1.697	0.024
	V–Co	3.807	10.72	3.353	
	V–V	2.483	5.50	3.528	
V_{20}–$Co(OH)_2$	V–O	4.151	5.77	1.702	0.028
	V–Co	3.651	14.43	3.639	
	V–V	2.519	5.84	3.449	

Table S4. Comparison of the OER performance of V_{20}–$Co(OH)_2$ and reported transition metal-based electrocatalysts. [1-16]

Catalysts	Substrate	Electrode preparation method	η_{50} (mV)	η_{100} (mV)	References
V_{20}–$Co(OH)_2$	Ni foam	Direct growth on substrate	268	293	This study
BS-1	Ni foam	Direct growth on substrate	-	293	*Adv. Sci., 2023, 10.4: 2206180.*
Co/CoMoN	Ni foam	Direct growth on substrate	-	303	*Adv. Sci.*, 2022, 9.11: 2105313.
VO_x/Ni_3S_2@NF	Ni foam	Drop casting on substrate (mass loading : 1.5 mg cm^{-2})	-	358	*J. Mater. Chem. A*, 2019, 7.17: 10534-10542.
V−CoP_2/CC	Carbon cloth	Direct growth on substrate	320	498	*Angew. Chem.-Int. Edit.*, 2022, 134.12: e202116233.
$FeNi(VO_4)x$@NF	Ni foam	Direct growth on substrate	-	274	*Small*, 2020, 16.32: 2002412.
P-CoVO@NF	Ni foam	Direct growth on substrate	296	317	*Adv. Mater.*, 2024, 36.41: 2408634.
Ce/NiV LDH	Ni foam	Direct growth on substrate	-	290	*Adv. Funct. Mater.,* 2024, 34.6: 2308533.
$CoV\text{-}Fe_{0.28}$	Glassy Carbon	Drop casting on substrate (mass loading : 0.28 mg cm^{-2})	278	-	*Adv. Energy Mater.,* 2020, 10.43: 2002215.
$Co(OH)_2/V_2O_5$	Glassy Carbon	Drop casting on substrate	370	-	*Adv. Sustain. Syst.,* 2023, 7.5: 2200473.
$FeCoNiMoVO_{x\text{-}1.5}$	Ni foam	Direct growth on substrate	-	300	*Chem. Eng. J.*, 2024, 495: 153408.
EBP/CoFeB	Ni foam	Drop casting on substrate (mass loading : 1.0 mg cm^{-2})	-	313	ACS nano, 2021, 15.7: 12418-12428.
$Fe_{0.052}Ni$-POMo	Ni foam	Direct growth on substrate	-	295	*Adv. Funct. Mater.*, 2021, 31.31: 2101792.
$Ni_xFe_yCo_{6-x-y}Mo_6C$/NF	Ni foam	Direct growth on substrate	-	275	*Appl. Catal. B-Environ.*, 2021, 290: 120049.

MoNi@Mo-NiO@NGF	Ni foam	Direct growth on substrate	308	-	*Chem. Eng. J.*, 2022, 440: 135847.
$CoSe_2$–D_{Fe}–V_{Co}	Glassy Carbon	Drop casting on substrate (mass loading : 2.0 mg cm^{-2})	-	520	*Nat. Commun.*, 2020, 11.1: 1664.
S-FeOOH/IF	Fe foam	Drop casting on substrate	287	308	*Adv. Funct. Mater.*, 2022, 32.26: 2112674.

Note. Electrode preparation methods and catalyst loadings are provided where available, as electrochemical performance can be affected by electrode configuration and fabrication conditions.

Table S5. Comparison of the OER performance of V_{20}–$Co(OH)_2$ with reported metal-based electrocatalysts prepared using low-/room-temperature synthesis strategies or cation exchange approaches. [17-30]

Catalysts	Substrate	Synthesis method / Temperature	Electrode preparation method	η (mV)	References
V_{20}–$Co(OH)_2$	Ni foam	Cation exchange / Room temperature	Direct growth on substrate	268 (@ 50 mA cm^{-2}) 293 (@ 100 mA cm^{-2})	This study
S-(Ni,Fe)OOH	Ni foam	One-pot solution-phase method / 500 °C	Direct growth on substrate	281 (@ 100 mA cm^{-2})	*Energy Environ. Sci.*, 2020, 13.10: 3439-3446.
$(NiFe)_3S_2$/NFF	NiFe foam	Stirring / 150 °C	Direct growth on substrate	241 (@ 100 mA cm^{-2})	*Adv. Funct. Mater.*, 2024, 34.34: 2400979.
CoOOH sphere	Carbon cloth	Diffusion / Room temperature	Drop casting (mass loading : 1 mg cm^{-2})	380 (@ 100 mA cm^{-2})	J. Energy Chem., 2022, 71: 89-97.
60Fe/NF	Ni foam	Displacement / Room temperature	Direct growth on substrate	270 (@ 50 mA cm^{-2}) 304 (@ 100 mA cm^{-2})	*Adv. Energy Mater.*, 2023, 13.39: 2301921.
NF-Na-Fe-Pt	Carbon paper	Step corrosive strategy / Room temperature	Direct growth on substrate	≈ 300 (@ 50 mA cm^{-2})	*Appl. Catal. B-Environ.*, 2021, 297: 120395.
NiFeP/NF	Ni foam	Immersion / Room temperature	Direct growth on substrate	248 (@ 10 mA cm^{-2})	*Inorg. Chem.*, 2024, 63.43: 20859-20869.
NF/$NiMoO_4$/NiS/$Ni(OH)_2$	Ni foam	Stirring / Room temperature	Direct growth on substrate	340 (@ 50 mA cm^{-2}) 390 (@ 100 mA cm^{-2})	*ACS Appl. Energ. Mater.*, 2024, 7.24: 11703-11713.
Pd-e-NiCo-PBA-C	Glassy carbon	Etching & Ion exchange / Room temperature	Drop casting (mass loading : 0.28 mg cm^{-2})	313 (@ 20 mA cm^{-2})	*Adv. Funct. Mater.*, 2021, 31.10: 2008989.

CoFe-LDH/CeO_2	Glassy carbon	Ion exchange / 70 °C	Drop casting (mass loading : 2.0 mg ml^{-2})	315 (@ 10 mA cm^{-2})	*J. Colloid Interface Sci.*, 2025, 683: 411-420.
Co-S_v-MoS_2	Carbon cloth	Cation exchange / 90 °C	Drop casting	261 (@ 20 mA cm^{-2})	*Small*, 2022, 18.39: 2203173.
CoNiFeO_x-NC	Carbon papaer	Ion exchange / 300 °C	Drop casting (mass loading : 100 µl cm^{-2})	265 (@ 50 mA cm^{-2})	*Appl. Catal. B-Environ.*, 2021, 287: 119953.
FeS-Ni_3S_2/NF	Ni foam	Chemical etching & Anion exchange / 100 °C	Direct growth on substrate	238 (@ 10 mA cm^{-2}) 290 (@ 100 mA cm^{-2})	*Adv. Mater. Interfaces*, 2019, 6.18: 1900788.
S/N-CMF@Fe_xCo_y Ni_{1-y}-MOF	Carbon paper	Cation exchange / 70 °C	Drop casting	296 (@ 10 mA cm^{-2})	*Adv. Mater.*, 2023, 35.19: 2207888.
S-CoO_x/NF	Ni foam	Ion exchange / Room temperature	Direct growth on substrate	370 (@ 100 mA cm^{-2})	Nano Energy, 2020, 71: 104652.

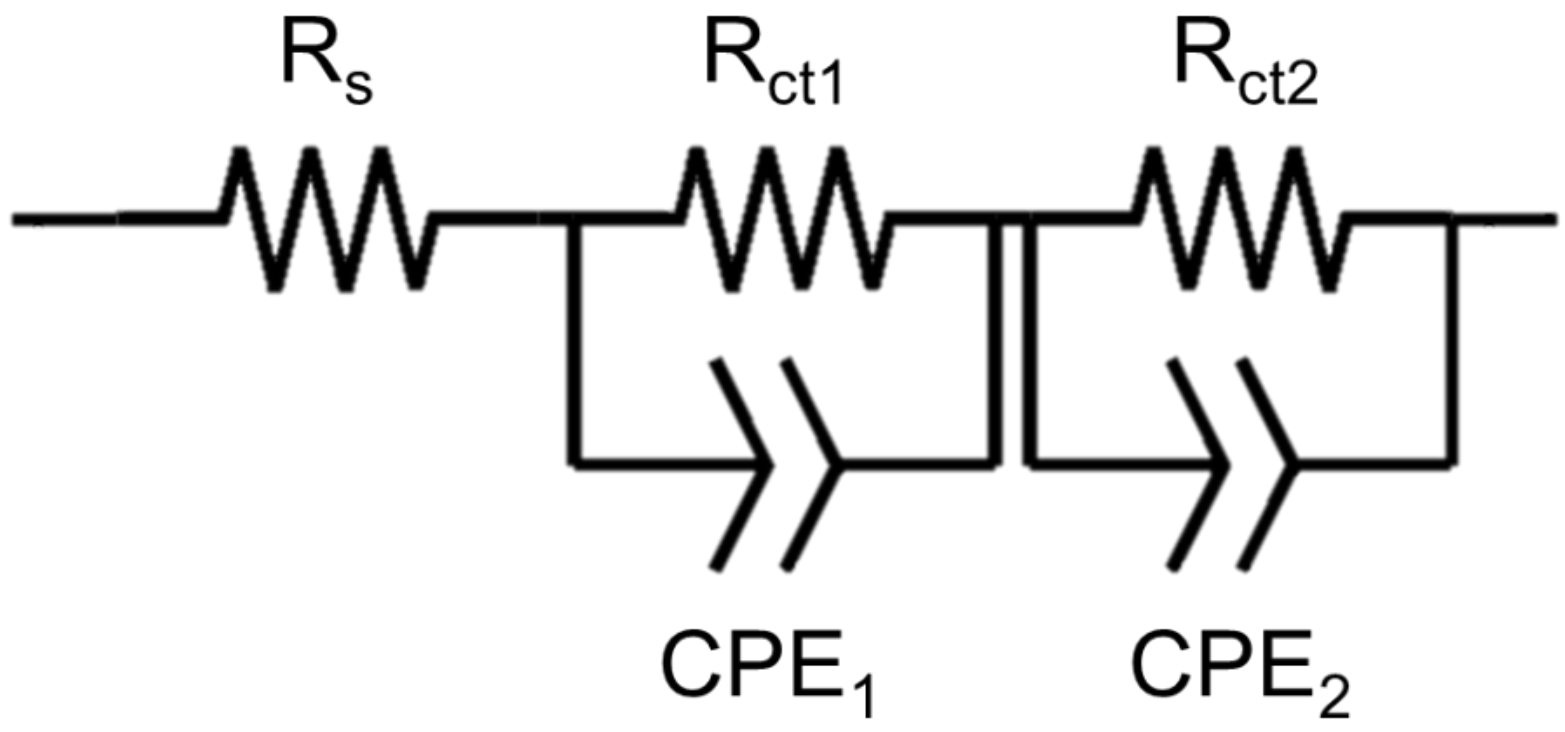

Figure S17. Electrical equivalent circuit model.

R_s : Electrolyte resistance, R_{ct1} : Electrooxidation and R_{ct2} : oxygen evolution reation

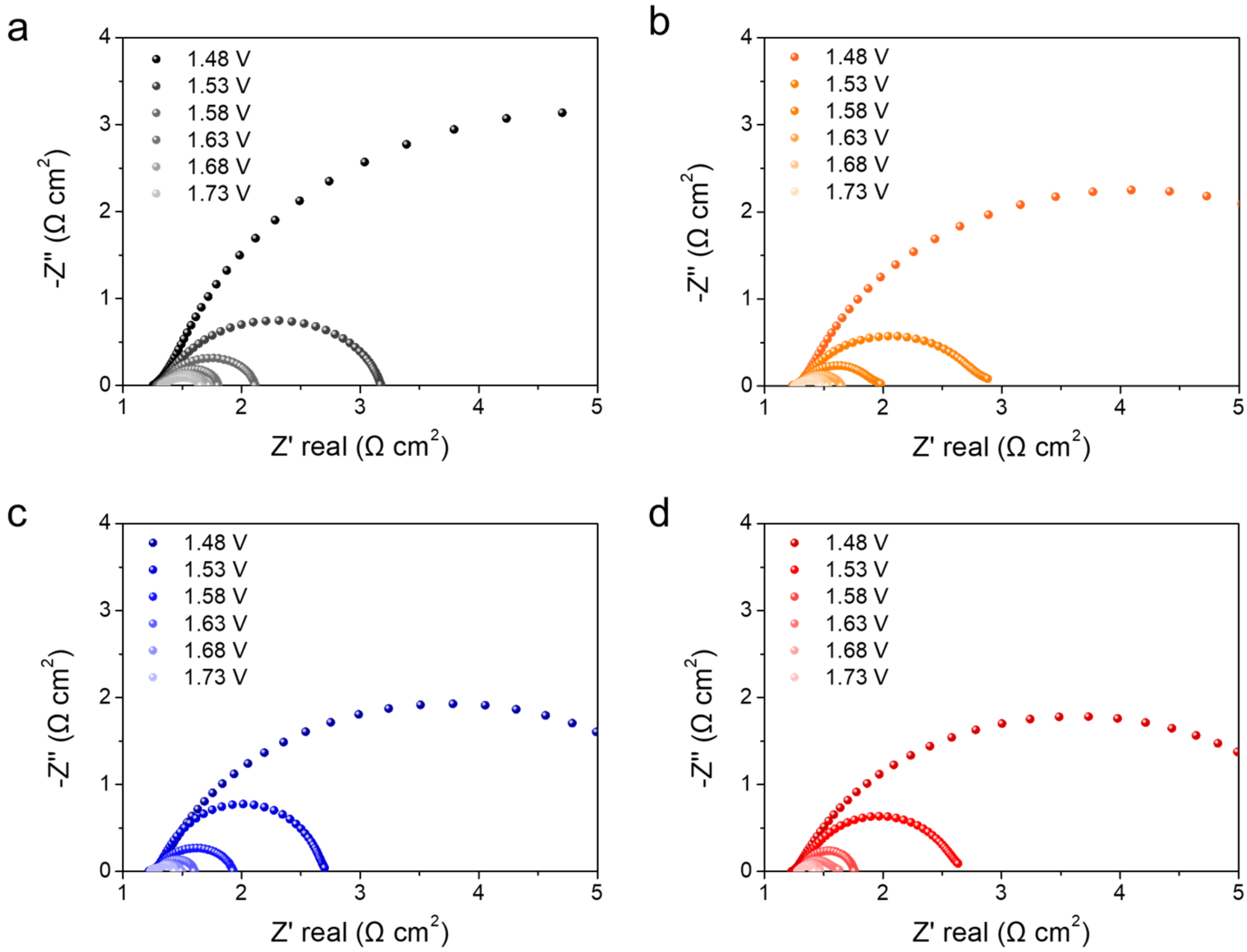

Figure S18. In situ EIS spectra of **a** ZIF-67, **b** V_{10}–$Co(OH)_2$, **c** V_{15}–$Co(OH)_2$, and **d** V_{20}–$Co(OH)_2$ at various potentials.

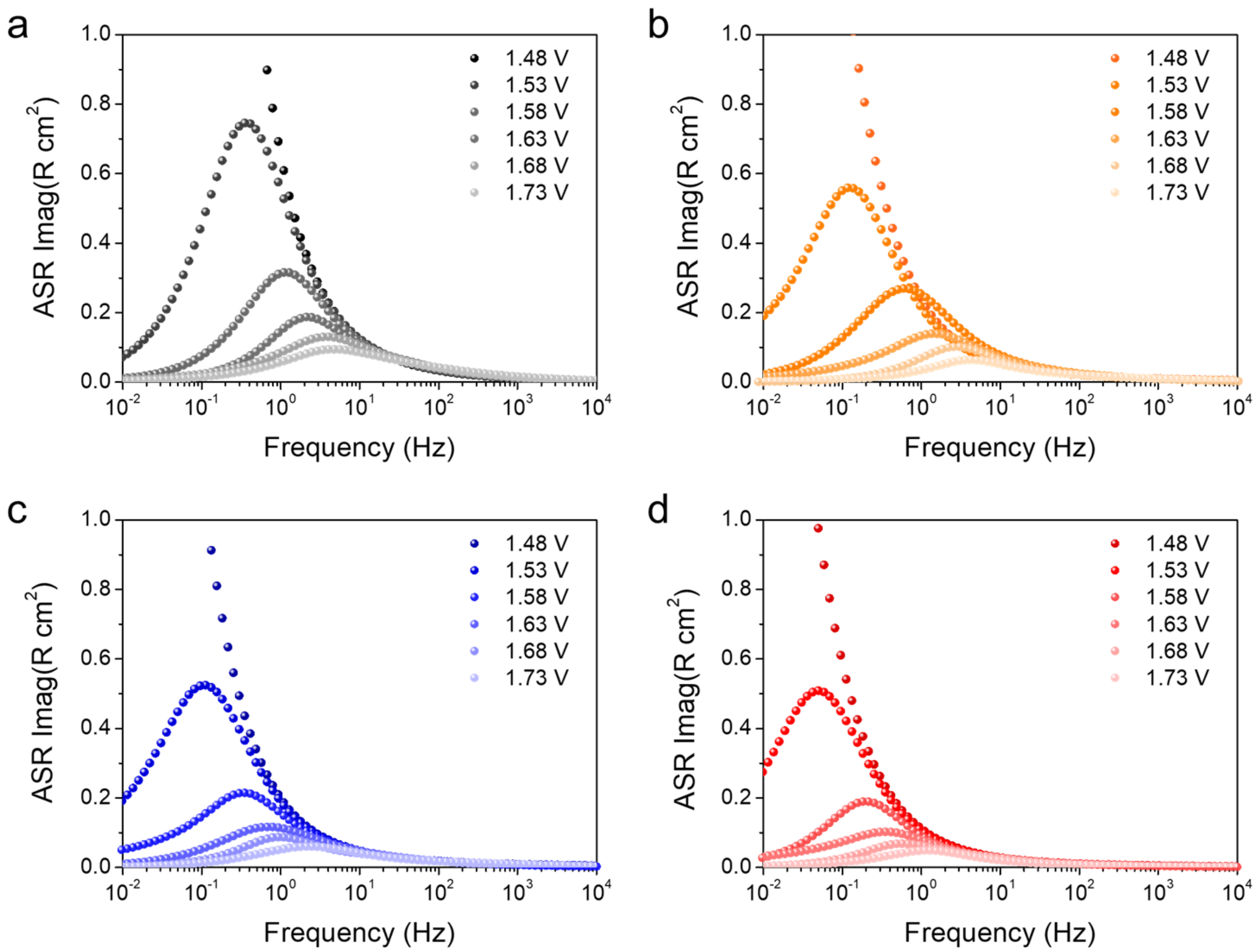

Figure S19. In situ bode plots of **a** ZIF-67, **b** V_{10}–$Co(OH)_2$, **c** V_{15}–$Co(OH)_2$, and **d** V_{20}–$Co(OH)_2$ at various potentials.

Table S6. Fitting results of in situ Nyquist plots recorded at various potentials in 1.0 M KOH electrolytes.

Electrode	Potential (V)	R_s (Ω cm^2)	R_{ct} (Ω cm^2)
ZIF-67	1.48	1.25	7.231
	1.53	1.28	1.891
	1.58	1.29	0.821
	1.63	1.30	0.501
	1.68	1.31	0.416
	1.73	1.32	0.333
V_{10}–$Co(OH)_2$	1.48	1.24	5.650
	1.53	1.25	1.635
	1.58	1.25	0.729
	1.63	1.25	0.400
	1.68	1.26	0.273
	1.73	1.25	0.223
V_{15}–$Co(OH)_2$	1.48	1.22	4.858
	1.53	1.26	1.445
	1.58	1.25	0.689
	1.63	1.25	0.360
	1.68	1.23	0.264
	1.73	1.23	0.206
V_{20}–$Co(OH)_2$	1.48	1.23	4.318
	1.53	1.27	1.368
	1.58	1.28	0.480
	1.63	1.28	0.213
	1.68	1.27	0.192
	1.73	1.28	0.146

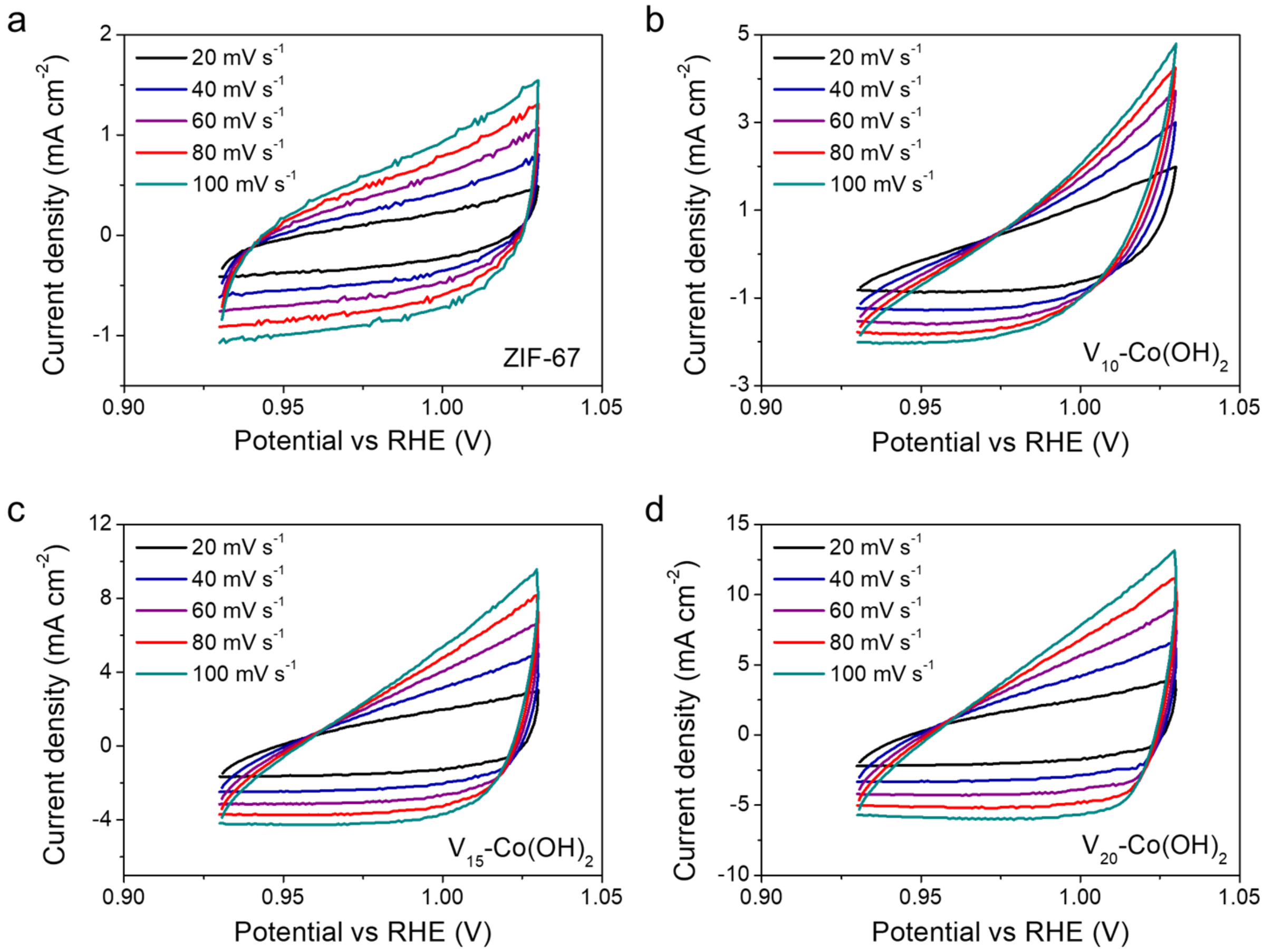

Figure S20. CV curves of **a** ZIF-67, **b** V_{10}–$Co(OH)_2$, **c** V_{15}–$Co(OH)_2$, and **d** V_{20}–$Co(OH)_2$ at different scan rates in 1.0 M KOH electrolytes.

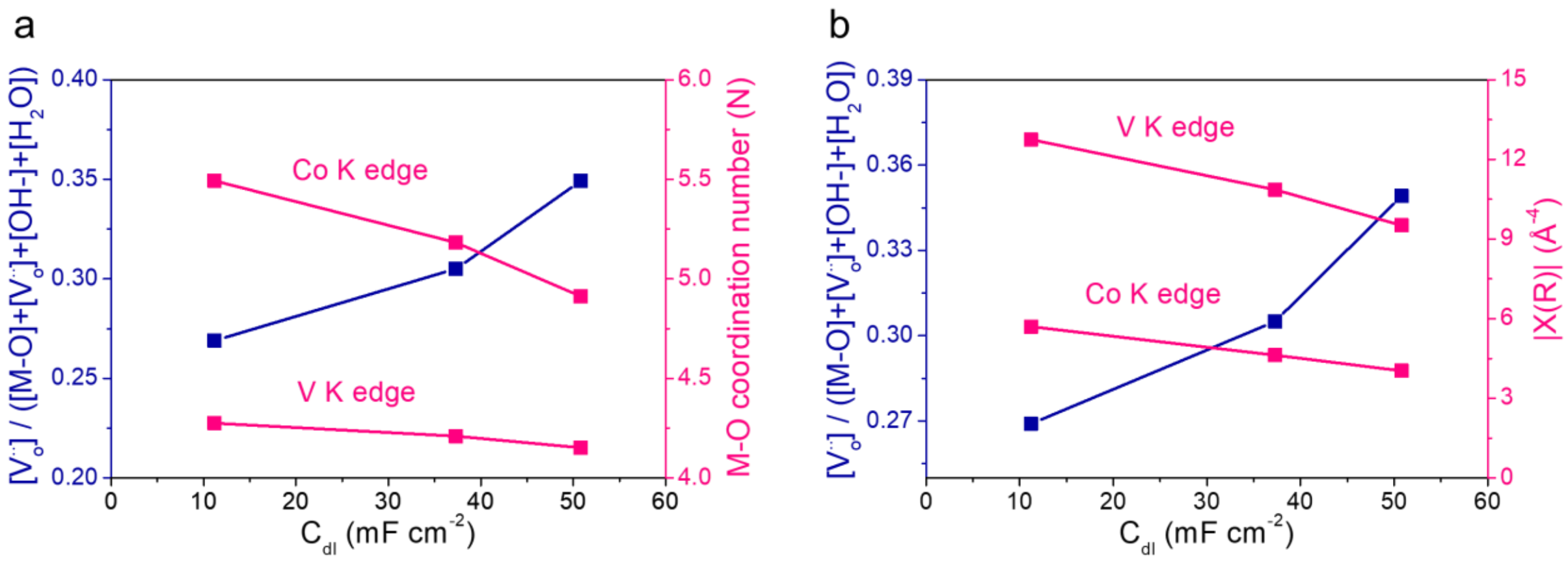

Figure S21. Relationship between **a** M-O coordination number (pink) and **b** M–O intensity of FT-EXAFS with oxygen vacancy based on O 1s XPS (blue) and C_{dl} value.

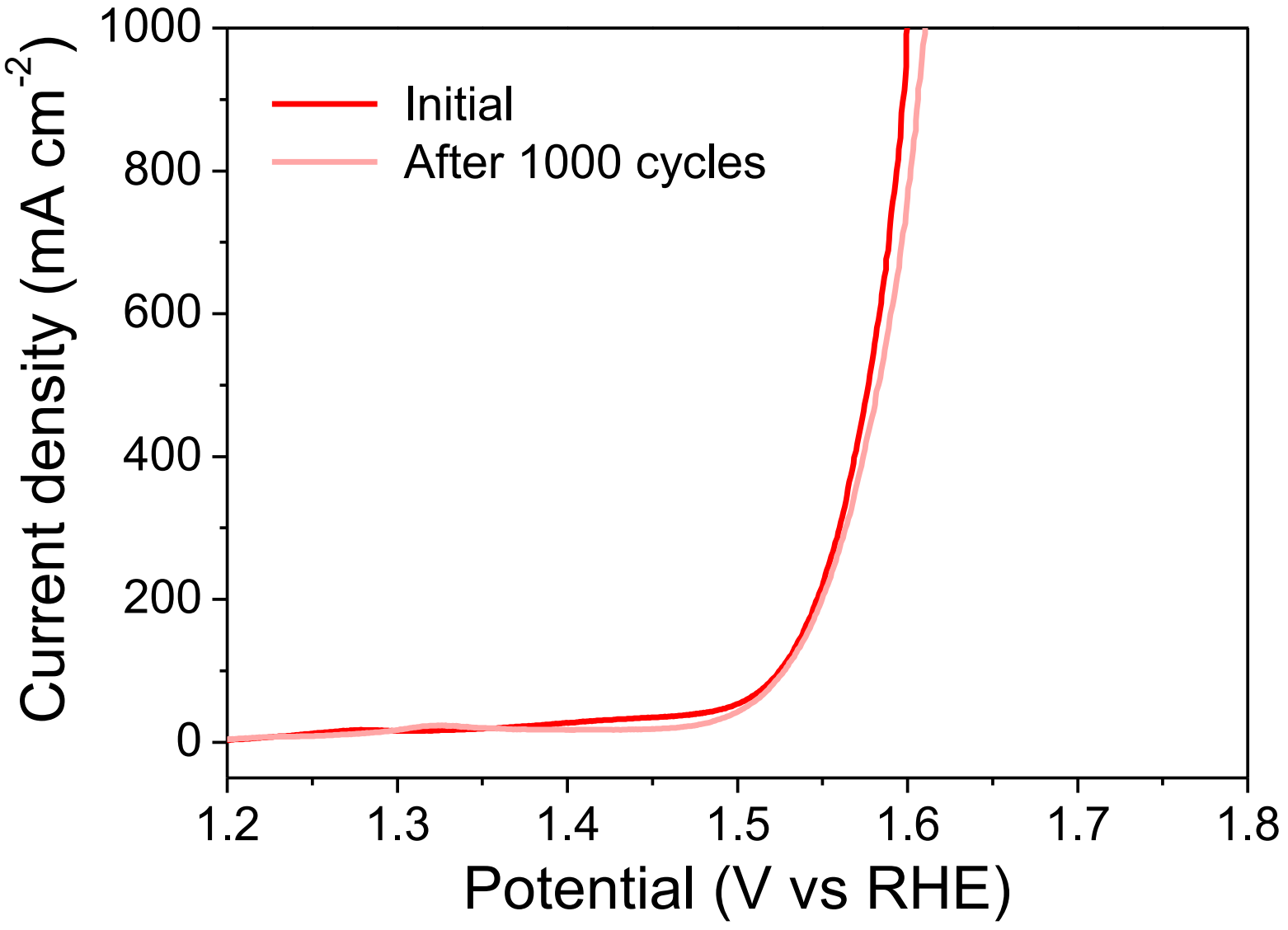

Figure S22. LSV curves of the initial V_{20}–$Co(OH)_2$ and after 1000 CV cycles with a potential window of 1.0–1.8 V.

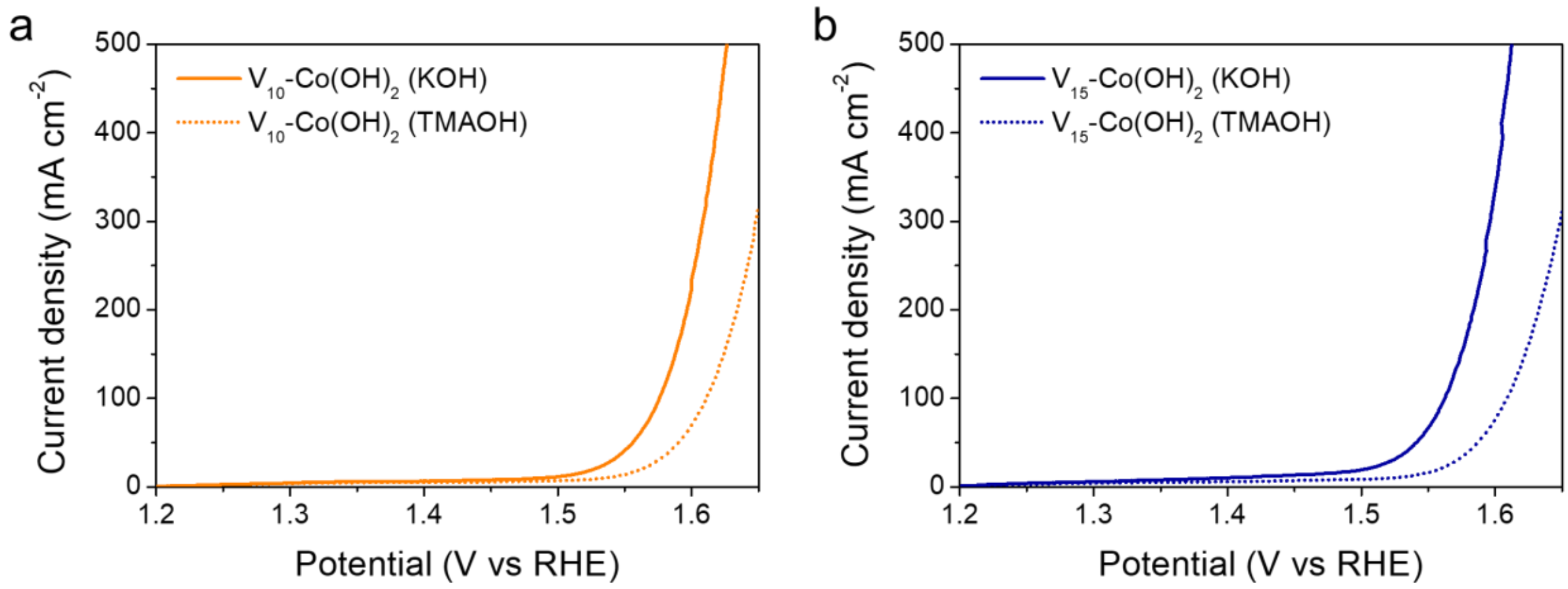

Figure S23. LSV profiles of **a** V_{10}–$Co(OH)_2$ and **b** V_{15}–$Co(OH)_2$ in 1.0 M KOH and 1.0 M TMAOH electrolytes.

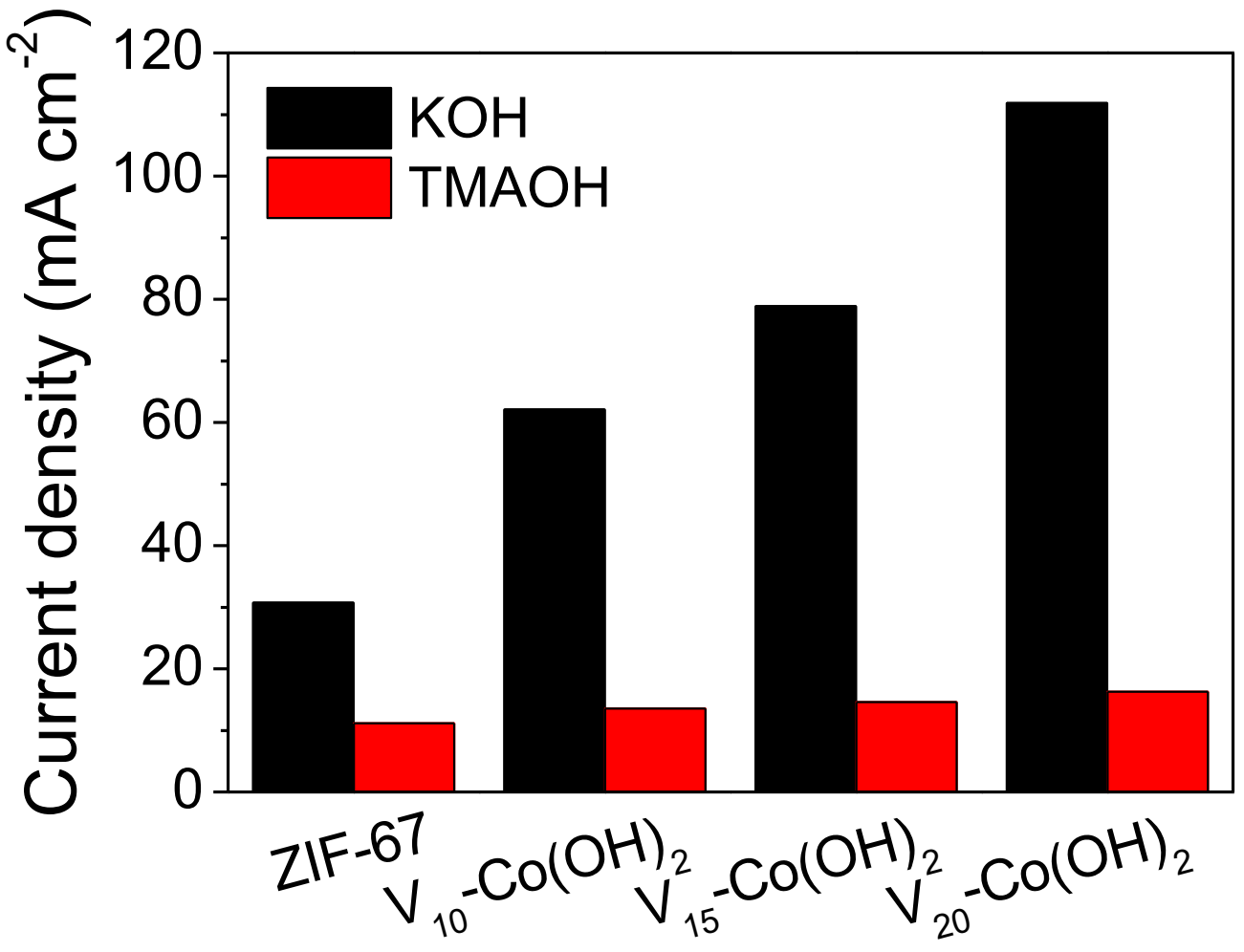

Figure S24. Corresponding overpotentials at 1.55 V vs RHE in 1.0 M KOH and 1.0 M TMAOH electrolytes.

Table S7. Comparison of current density at 1.55 V vs RHE in 1.0 M KOH and 1.0 M TMAOH electrolytes.

Electrode	Current density (mA cm^{-2})		Degradation rate (%)
	1 M KOH	1 M TMAOH	
ZIF-67	22.13	11.19	49.44
V_{10}–$Co(OH)_2$	40.55	13.58	66.51
V_{15}–$Co(OH)_2$	66.30	15.83	76.12
V_{20}–$Co(OH)_2$	111.84	16.27	85.46

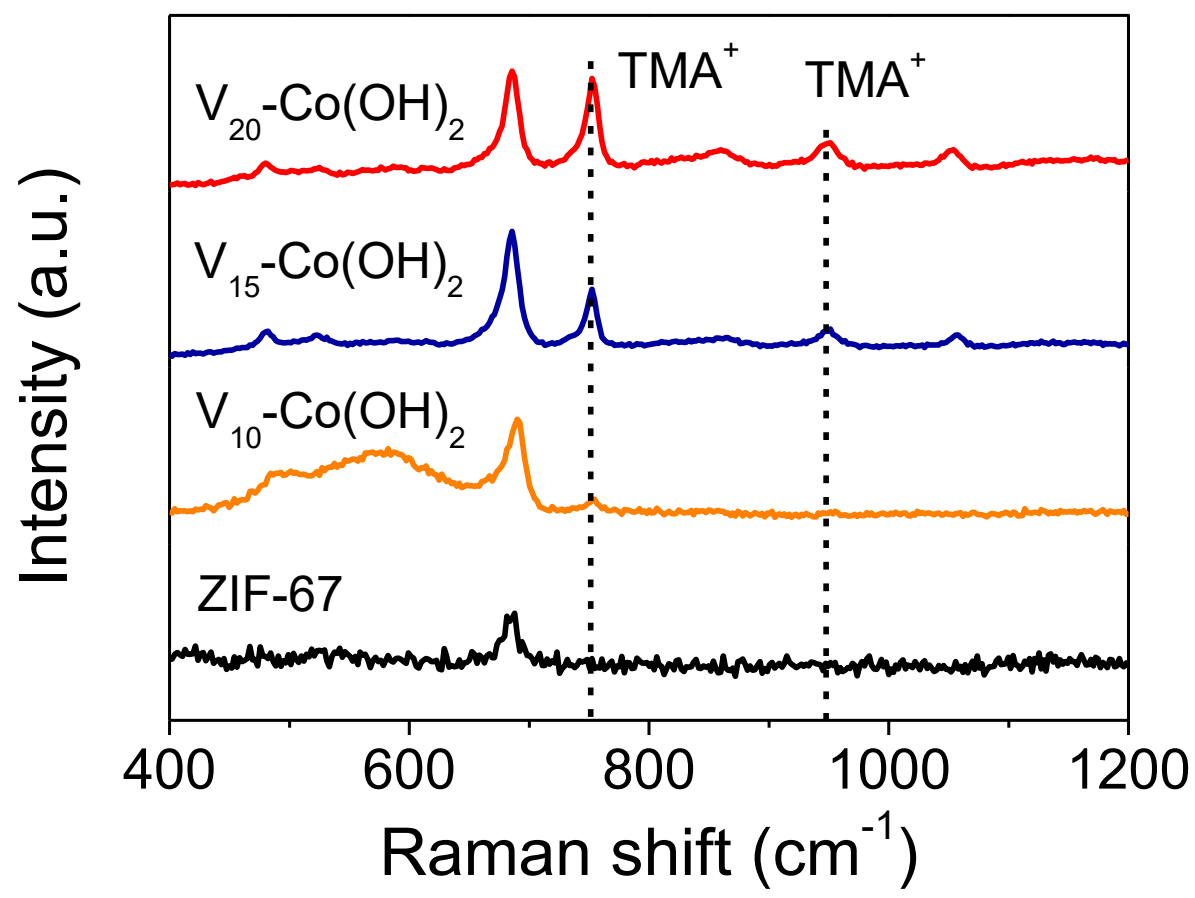

Figure S25. Raman spectra of the pristine ZIF-67 and vanadium-exchanged $Co(OH)_2$ electrodes in 1.0 M TMAOH electrolyte after chronoamperometry under 1.53 V vs RHE for 30 min.

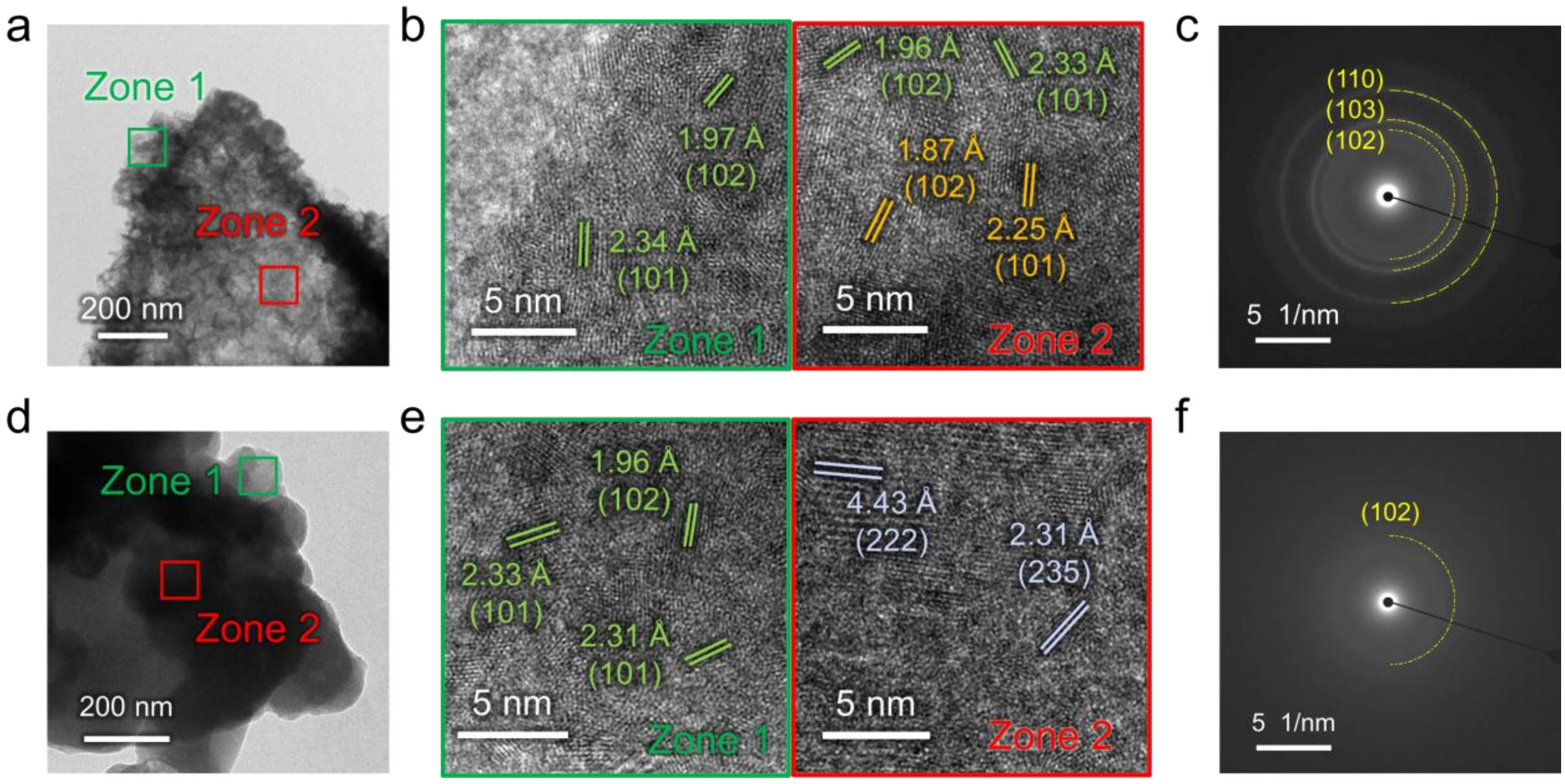

Figure S26. Structural characterization of pristine ZIF-67 and V_{20}-$Co(OH)_2$ after the stability test. **a** TEM image of V_{20}-$Co(OH)_2$. **b** HR-TEM images showing CoOOH-like oxyhydroxide in region 1 (green) and the coexistence of CoOOH-like oxyhydroxide (green) and $Co(OH)_2$ (orange) in region 2 (red). **c** SAED pattern of V_{20}-$Co(OH)_2$ showing $Co(OH)_2$ and CoOOH-like diffraction features. **d** TEM image of pristine ZIF-67. **e** HR-TEM images showing partial CoOOH-like oxyhydroxide (green). **f** SAED pattern of pristine ZIF-67 showing CoOOH-like diffraction features.

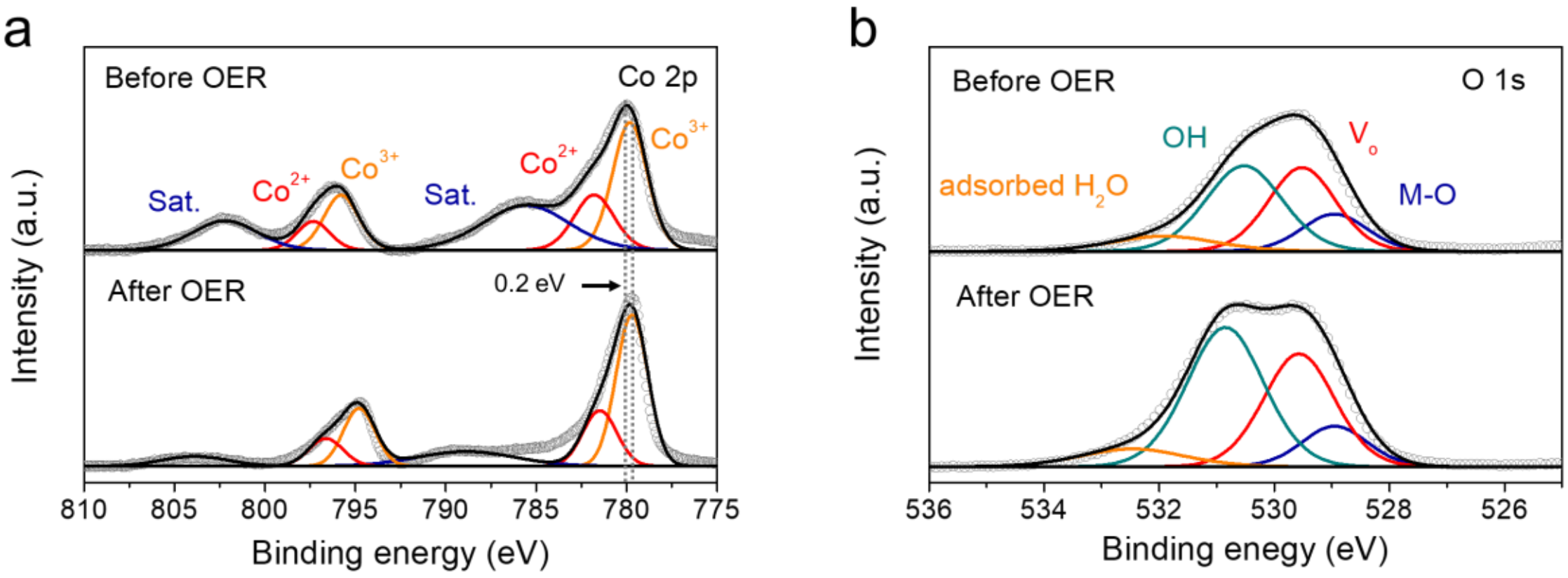

Figure S27. XPS spectra of V_{20}–$Co(OH)_2$ before and after the OER stability test. **a** Co 2p and **b** O 1s spectra. The Co 2p spectrum after the stability test shows a negative shift of 0.2 eV in binding energy.

Table S8. Relative fraction of Co^{3+} species and oxygen vacancy-related components before and after the OER stability test. The Co^{3+} fraction was calculated from the Co 2p spectra using $[Co^{3+}]/([Co^{2+}]+[Co^{3+}])$. The oxygen vacancy-related component was estimated from the O 1s spectra using $[V_O^{\cdot\cdot}]/([M\text{-}O]+[V_O^{\cdot\cdot}]+[OH^-]+[H_2O])$.

Relative ratio	Co^{3+}	$V_O^{\cdot\cdot}$
Before OER	0.67	0.35
After OER	0.71	0.36

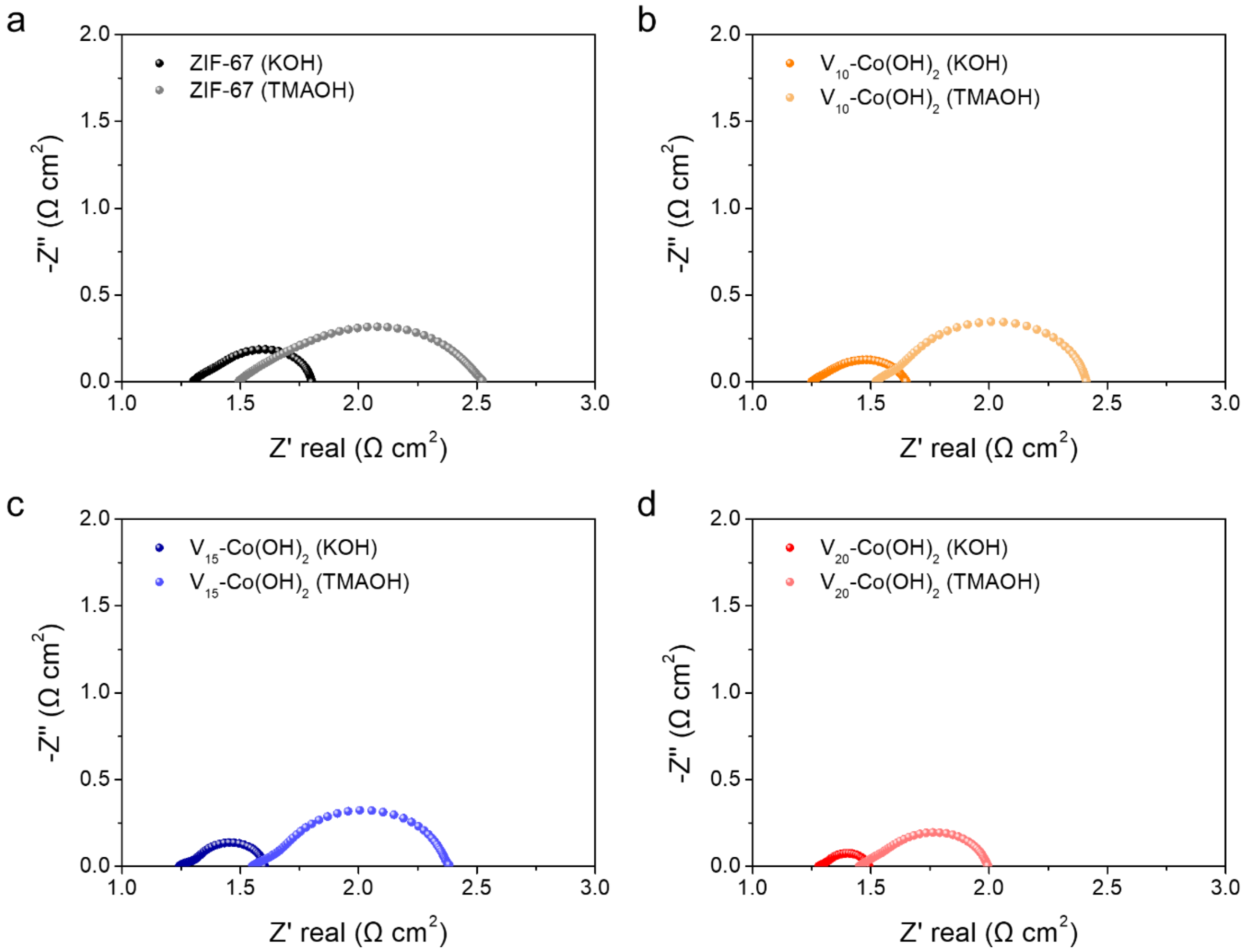

Figure S28. Nyquist plots of **a** pristine ZIF-67, **b** V_{10}–$Co(OH)_2$, **c** V_{15}–$Co(OH)_2$, and **d** V_{20}–$Co(OH)_2$ at 1.63 V vs RHE in 1 M KOH and 1 M TMAOH.

Table S9. Comparison of changes in charge transfer resistance according to differences in electrolytes.

Electrode	R_{ct}		Rate of increase (%)
	1 M KOH	1 M TMAOH	
ZIF-67	0.501	1.03	105.63
V_{10}–$Co(OH)_2$	0.400	0.889	122.20
V_{15}–$Co(OH)_2$	0.360	0.831	130.66
V_{20}–$Co(OH)_2$	0.213	0.543	154.72

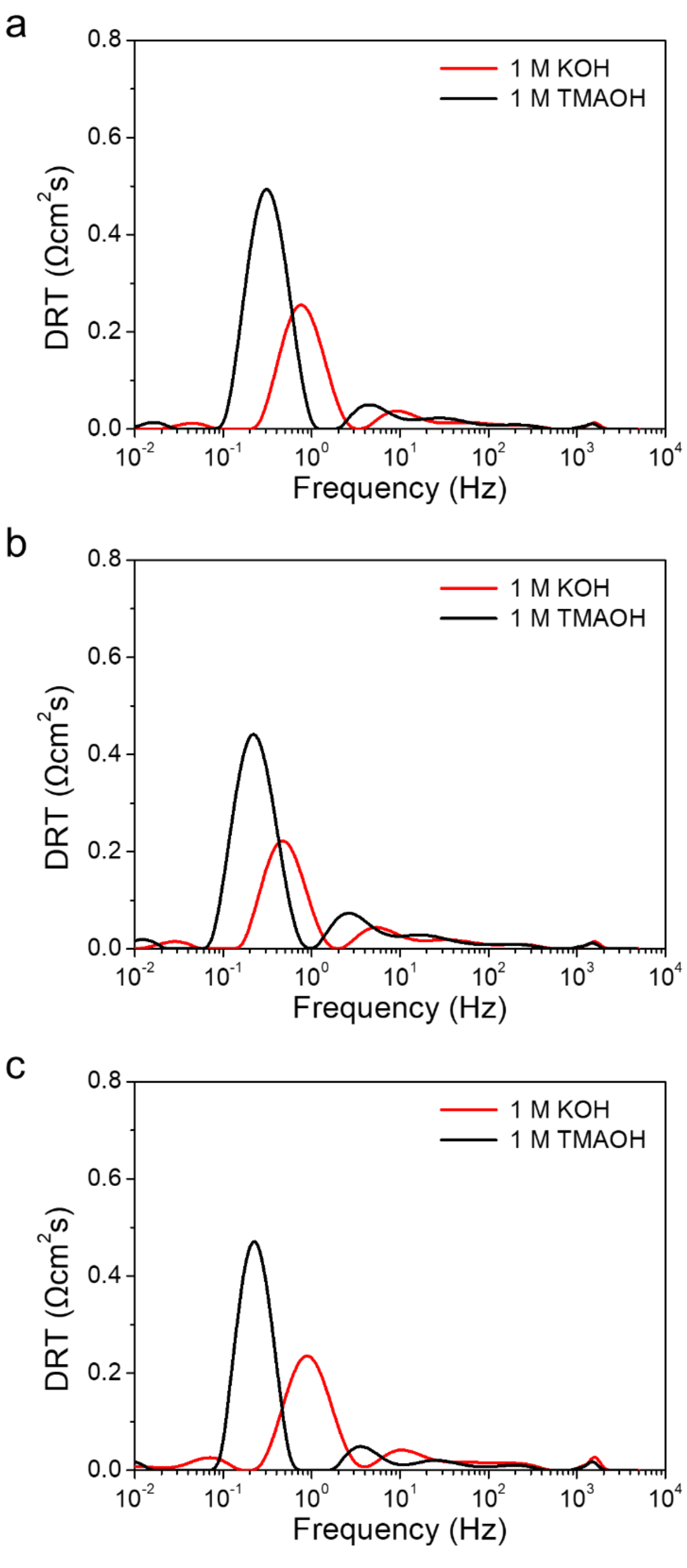

Figure S29. DRT analysis of **a** V_{10}–$Co(OH)_2$, **b** V_{15}–$Co(OH)_2$, and **c** V_{20}–$Co(OH)_2$ at 1.63 V vs RHE in 1 M KOH and 1 M TMAOH.

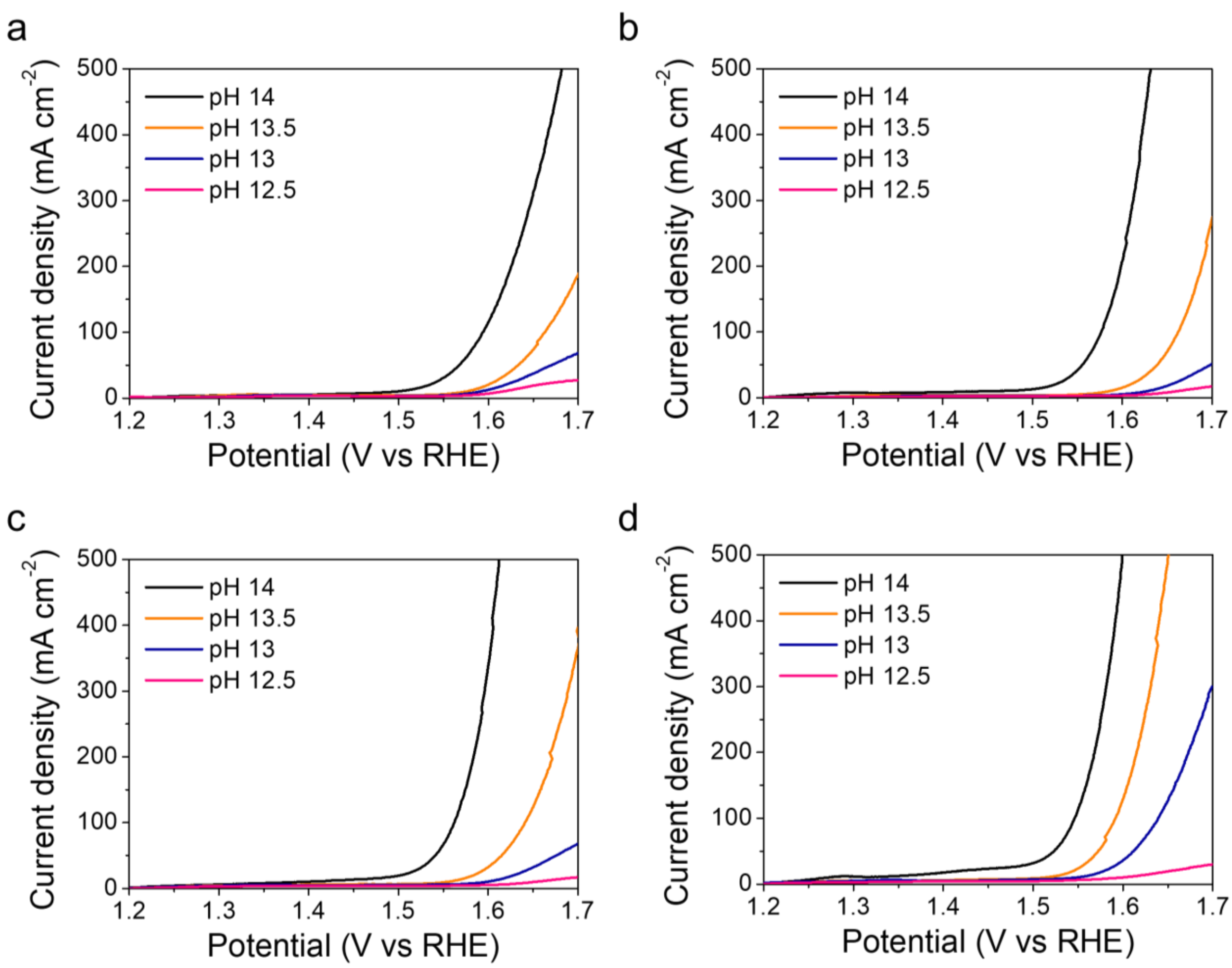

Figure S30. pH dependence on OER activity. The polarization curves of **a** pristine ZIF-67, **b** V_{10}–$Co(OH)_2$, **c** V_{15}–$Co(OH)_2$ and **d** V_{20}–$Co(OH)_2$ under various pH conditions.

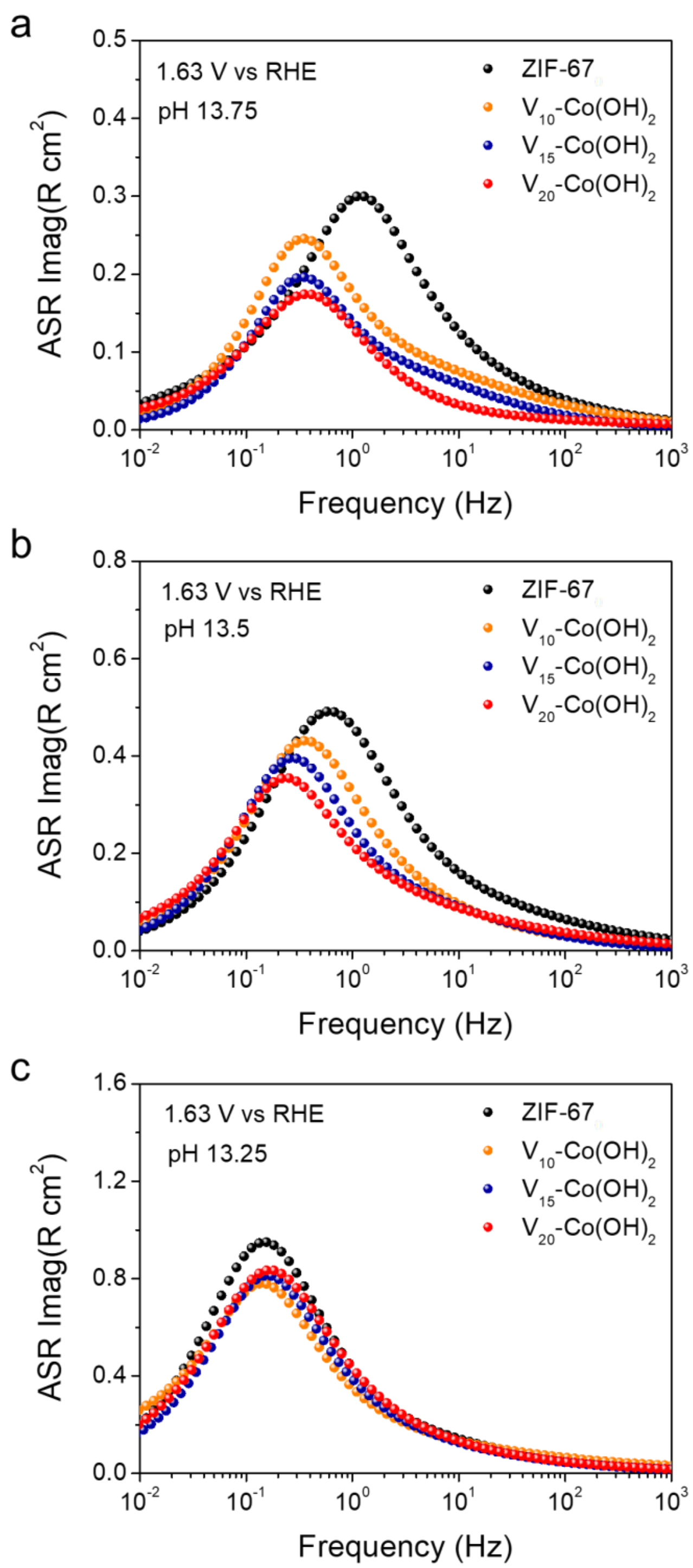

Figures S31. Bode plots of pristine ZIF-67, V_{10}–$Co(OH)_2$, V_{15}–$Co(OH)_2$, and V_{20}–$Co(OH)_2$ in **a** pH 13.75, **b** pH 13.5, and **c** pH 13.25 at 1.63 V vs RHE.

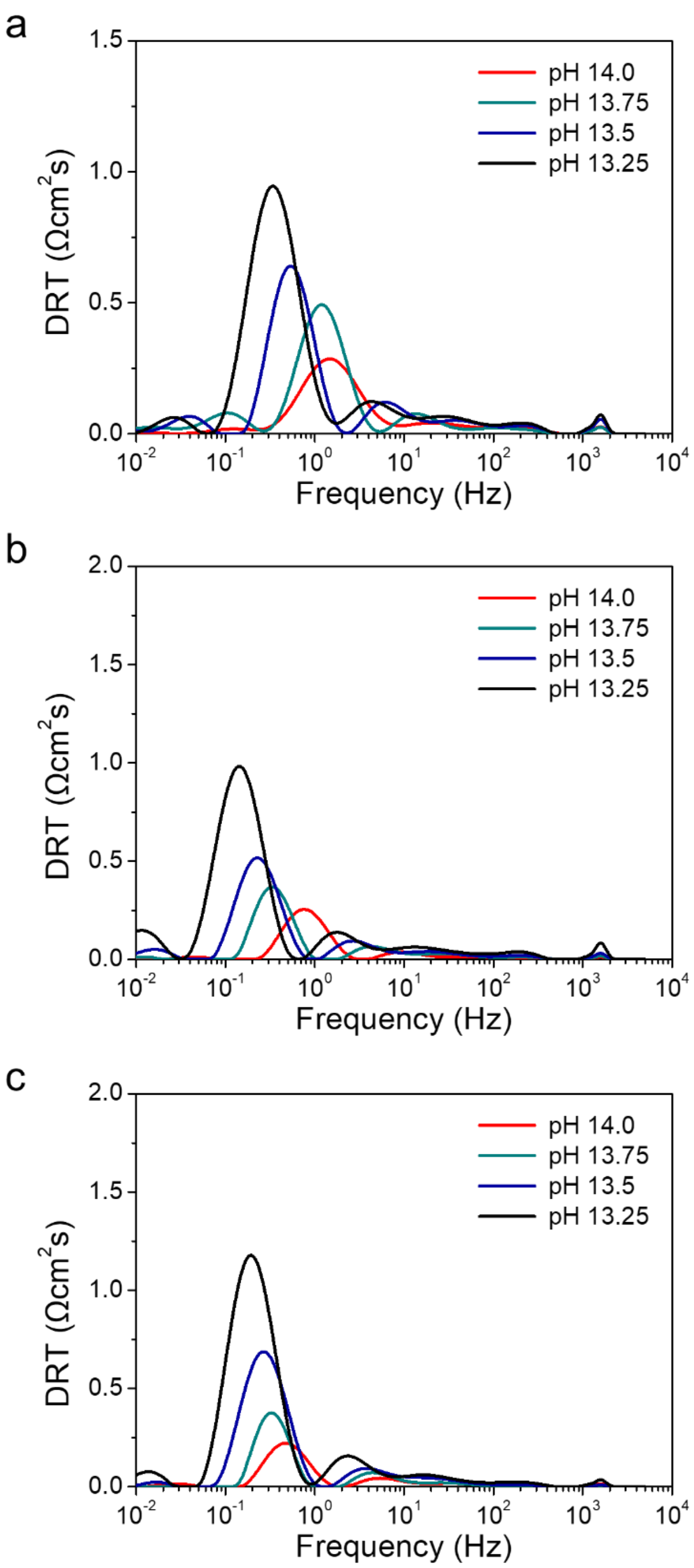

Figure S32. DRT analysis of **a** pristine ZIF-67, **b** V_{10}–$Co(OH)_2$, and **c** V_{15}–$Co(OH)_2$ at 1.63 V vs RHE in 1 M KOH electrolytes with different pH values (13.25–14.0).

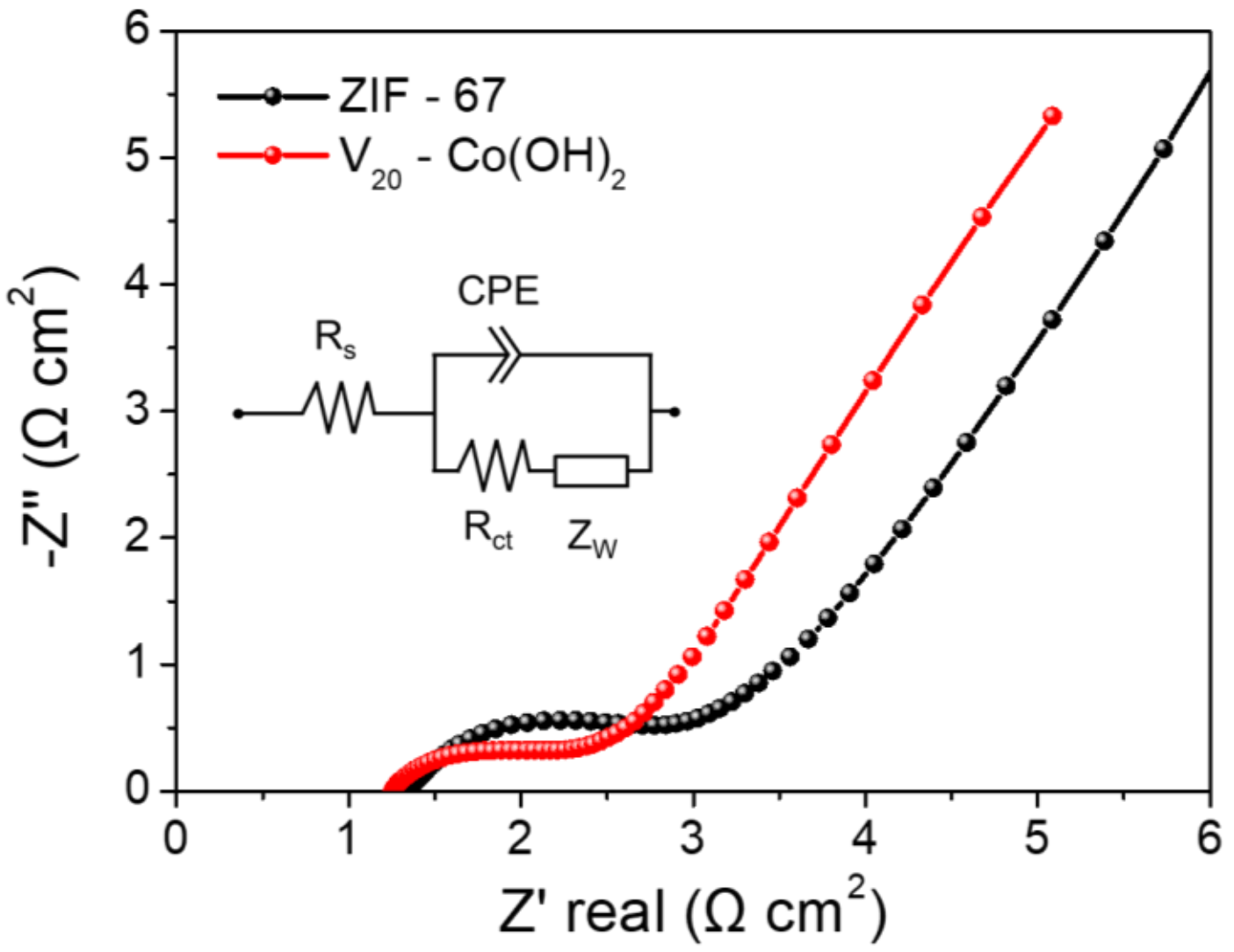

Figure S33. Nyquist plot of the supercapacitor devices assembled from pristine ZIF-67 and V_{20}–$Co(OH)_2$.

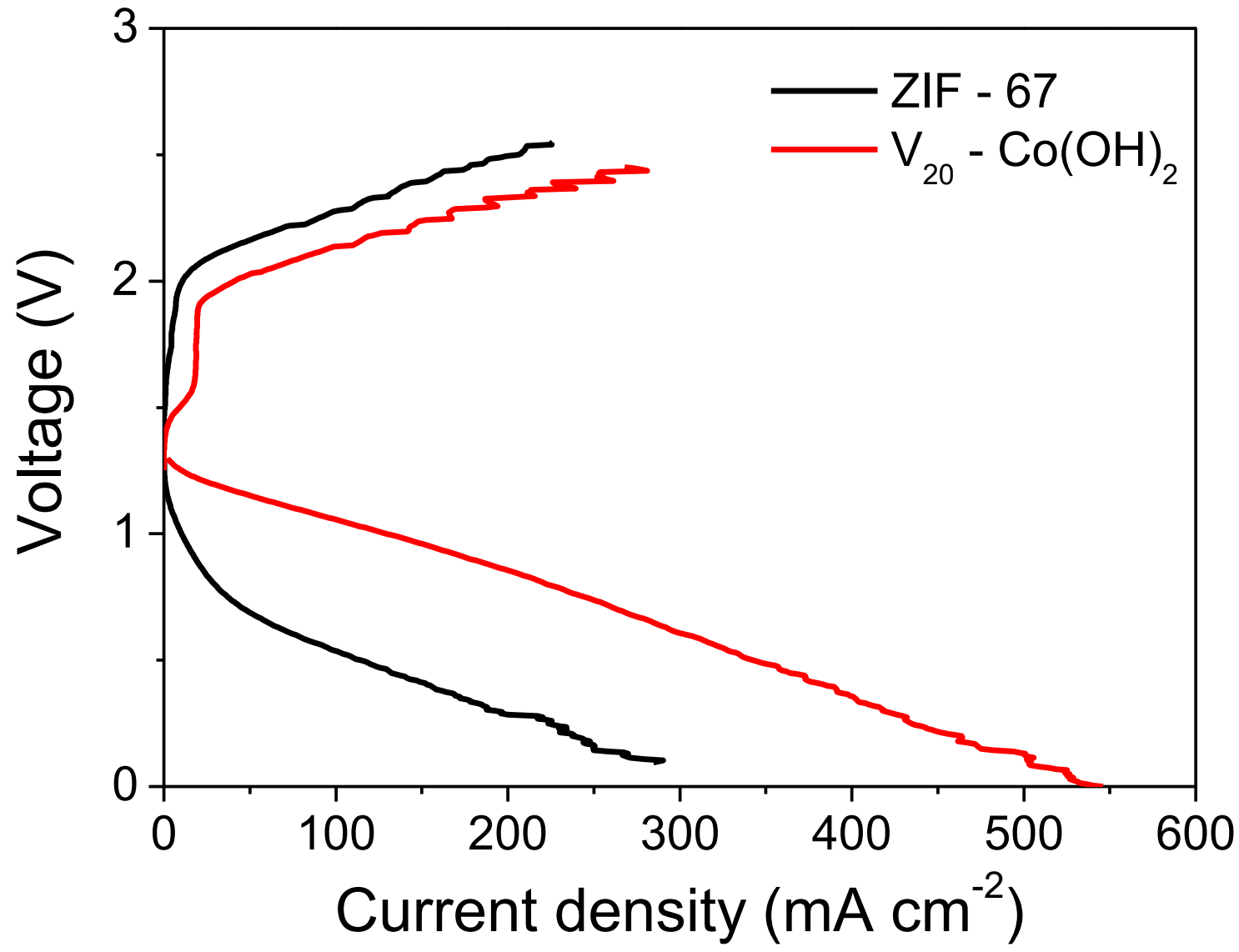

Figure S34. Charge–discharge polarization curves of pristine ZIF-67 and V_{20}–$Co(OH)_2$.

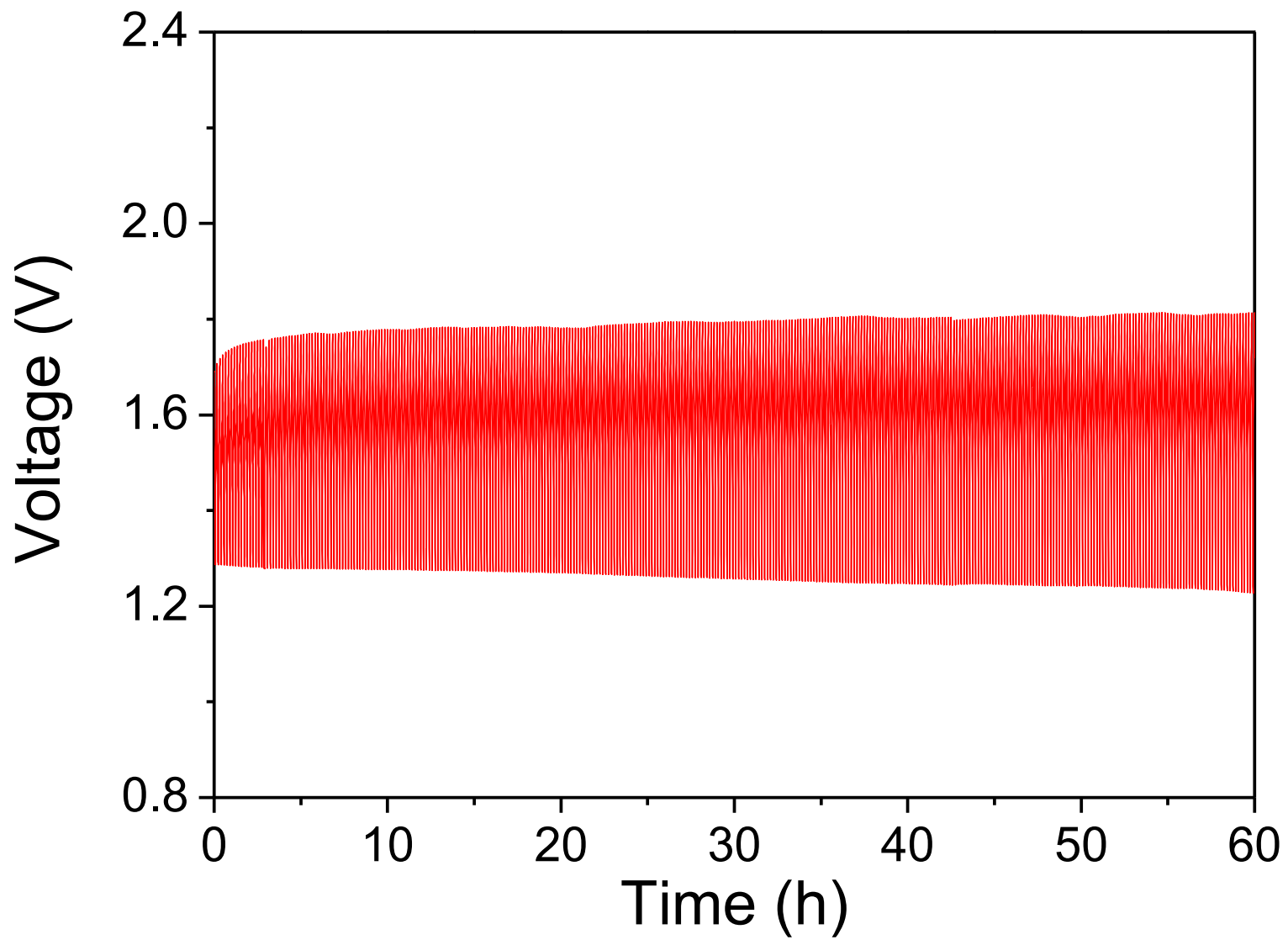

Figure S35. Charge–discharge cycling performance of V_{20}–$Co(OH)_2$-based zinc–air batteries at 5 mA cm^{-2}.

Table S10. Comparison of the supercapacitor performance in this study with those of other reported transition metal-based electrocatalysts. [31-36]

Catalysts	Electrode preparation method	Areal capacitance (mF cm^{-2})	Energy density (mWh cm^{-2})	Power density (mW cm^{-2})	References
V_{20}–$Co(OH)_2$	Direct growth on substrate	1227.31 (@ 2 mA cm^{-2})	0.015	0.301	This study
ZIF-67	Direct growth on substrate	275.62 (@ 2 mA cm^{-2})	0.0036	0.305	This study
S-NMN-2	Direct growth on substrate	1952 (@ 1 mA cm^{-2})	0.189	1.6	*ACS Appl. Energ. Mater.*, 2023, 6.7: 3789-3798.
CW-P-9.24	Drop casting (mass loading : 17.17 mg cm^{-2})	4700 (@ 1 mA cm^{-2})	0.94	0.6	*Chem. Eng. J.*, 2021, 414: 128767.
PMSC-4	Drop casting	1253.5 (@ 0.10 mA cm^{-2})	0.011	0.0403	*J. Colloid Interface Sci.*, 2023, 630: 586-594.
CC@CoS_2-TAA	Direct growth on substrate	1290 (@ 3 mA cm^{-2})	3.16	1.61×10^3	*Chem. Eng. J.*, 2020, 400: 125856.
CDs/NiFeP	Direct growth on substrate	2589 (@ 2 mA cm^{-2})	0.21	4.25	*J. Alloy. Compd.*, 2023, 958: 170525.
Fe_2O_3	Drop casting (mass loading : 5.0 mg cm^{-2})	1687 (@ 2 mA cm^{-2})	-	-	*J. Mater. Chem. A*, 2017, 5.43: 22648-22653.

Table S11. Comparison of the zinc-air battery performance of V_{20}-$Co(OH)_2$ with reported transition metal-based electrocatalysts. [37-48]

Catalysts	Electrode preparation method	Peak power density ($mW\ cm^{-2}$)	Specific capacity ($mAh\ g^{-1}$)	References
V_{20}–$Co(OH)_2$	Direct growth on substrate	205.7	908.7	This study
ZIF–67	Direct growth on substrate	89.7	762.9	This study
Co/N@CNTs @CNMF-800	Drop casting (mass loading : 1.06 mg cm^{-2})	133	777	*Adv. Funct. Mater.*, 2020, 30.36: 2003407.
CoS_x/Co-NC-800	Drop casting (mass loading : 1.0 mg cm^{-2})	103	744.7	*Adv. Funct. Mater.*, 2019, 29.38: 1904481.
PyN-GDY	Drop casting (mass loading : 1.0 mg cm^{-2})	130	647	*Appl. Catal. B-Environ.*, 2020, 261: 118234.
Fe/Ni-N_x/OC	Drop casting (mass loading : 1.0 mg cm^{-2})	148	712	*Adv. Mater.*, 2020, 32.42: 2004670.
Co_p@CoNC	Drop casting (mass loading : 1.0 mg cm^{-2})	188.8	794.1	*Energy Storage Mater.*, 2022, 46: 553-562.
HPFe-N-C	Drop casting (mass loading : 2.0 mg cm^{-2})	160	672	*Appl. Catal. B-Environ.*, 2022, 305: 121040.
Fe-SA/Meso-C	Drop casting (mass loading : 1.0 mg cm^{-2})	166.2	776.1	*Carbon*, 2022, 191: 393-402.
$Pd_1Mo_1Ta_2O_\alpha$	Drop casting (mass loading : 0.5 mg cm^{-2})	114.8	1050	*Adv. Funct. Mater.*, 2025, 35.13: 2418211.
FeNi@NCNT	Drop casting (mass loading : 1.0 mg cm^{-2})	200	788	*Small*, 2021, 17.4: 2006183.
FeCoNC/D	Drop casting (mass loading : 1.0 mg cm^{-2})	157	725	*Appl. Catal. B-Environ.*, 2022, 315: 121501.

CoZn-NC	Drop casting (mass loading : 1.2 mg cm^{-2})	152	578	*Adv. Funct. Mater.*, 2017, 27.37: 1700795.
FeCo/NUCSs	Drop casting (mass loading : 1.0 mg cm^{-2})	152.38	791.86	*Appl. Catal. B-Environ.*, 2022, 316: 121687.

REFERENCES

1. L. Li, P. C. M. Laan, X. Yan, X. Cao, M. J. Mekkering, K. Zhao, L. Ke, X. Jiang, X. Wu, L. Li, L. Xue, Z. Wang, G. Rothenberg and N. Yan. "High-Rate Alkaline Water Electrolysis at Industrially Relevant Conditions Enabled by Superaerophobic Electrode Assembly" *Adv. Sci.* (2023). **10**, e2206180 10.1002/advs.202206180.

2. H. Ma, Z. Chen, Z. Wang, C. V. Singh and Q. Jiang. "Interface Engineering of Co/CoMoN/NF Heterostructures for High-Performance Electrochemical Overall Water Splitting" *Adv. Sci.* (2022). **9**, e2105313 10.1002/advs.202105313.

3. Y. Niu, W. Li, X. Wu, B. Feng, Y. Yu, W. Hu and C. M. Li. "Amorphous nickel sulfide nanosheets with embedded vanadium oxide nanocrystals on nickel foam for efficient electrochemical water oxidation" *J. Mater. Chem. A* (2019). **7**, 10534–10542 10.1039/c8ta12483c.

4. Y. Wang, Y. Jiao, H. Yan, G. Yang, C. Tian, A. Wu, Y. Liu and H. Fu. "Vanadium-Incorporated CoP(2) with Lattice Expansion for Highly Efficient Acidic Overall Water Splitting" *Angew. Chem.-Int. Edit.* (2022). **61**, e202116233 10.1002/anie.202116233.

5. K. Dastafkan, Q. Meyer, X. Chen and C. Zhao. "Efficient Oxygen Evolution and Gas Bubble Release Achieved by a Low Gas Bubble Adhesive Iron-Nickel Vanadate Electrocatalyst" *Small* (2020). **16**, 2002412 10.1002/smll.202002412.

6. Z. Liang, D. Shen, Y. Wei, F. Sun, Y. Xie, L. Wang and H. Fu. "Modulating the Electronic Structure of Cobalt-Vanadium Bimetal Catalysts for High-Stable Anion Exchange Membrane Water Electrolyzer" *Adv. Mater.* (2024). **36**, 2408634 10.1002/adma.202408634.

7. K. Zeng, M. Chao, M. Tian, J. Yan, M. H. Rummeli, P. Strasser and R. Yang. "Atomically Dispersed Cerium Sites Immobilized on Vanadium Vacancies of Monolayer Nickel-Vanadium Layered Double Hydroxide: Accelerating Water Splitting Kinetics" *Adv. Funct. Mater.* (2023). **34**, 2308533 10.1002/adfm.202308533.

8. M. Kuang, J. Zhang, D. Liu, H. Tan, K. N. Dinh, L. Yang, H. Ren, W. Huang, W. Fang, J. Yao, X. Hao, J. Xu, C. Liu, L. Song, B. Liu and Q. Yan. "Amorphous/Crystalline Heterostructured Cobalt-Vanadium-Iron (Oxy)hydroxides for Highly Efficient Oxygen Evolution Reaction" *Adv. Energy Mater.* (2020). **10**, 2002215 10.1002/aenm.202002215.

9. J. Tang, Q. Ruan, H. Yu and C. Huang. "Activating $Co(OH)_2$ Active Sites by Coupled with V_2O_5 to Boost Highly Efficient Oxygen Evolution Reaction" *Adv. Sustain. Syst.* (2023). **7**, 2200473 10.1002/adsu.202200473.

10. C. Feng, Y. Zhou, Z. Xie, Z. Yang, L. Zou, P. Wang, W. Lian, P. Xiaokaiti, Y. Kansha, A. Abudula and G. Guan. "Vanadium boosted high-entropy amorphous FeCoNiMoV oxide for ampere-level seawater oxidation" *Chem. Eng. J.* (2024). **495**, 153408 10.1016/j.cej.2024.153408.

11. H. Chen, J. Chen, P. Ning, X. Chen, J. Liang, X. Yao, D. Chen, L. Qin, Y. Huang and Z. Wen. "2D Heterostructure of Amorphous CoFeB Coating Black Phosphorus Nanosheets with Optimal Oxygen

Intermediate Absorption for Improved Electrocatalytic Water Oxidation" *ACS Nano* (2021). **15**, 12418–12428 10.1021/acsnano.1c04715.
12. X. Liu, F. Xia, R. Guo, M. Huang, J. Meng, J. Wu and L. Mai. "Ligand and Anion Co-Leaching Induced Complete Reconstruction of Polyoxomolybdate-Organic Complex Oxygen-Evolving Pre-Catalysts" *Adv. Funct. Mater.* (2021). **31**, 2200473 10.1002/adfm.202101792.
13. L.-G. He, P.-Y. Cheng, C.-C. Cheng, C.-L. Huang, C.-T. Hsieh and S.-Y. Lu. "($Ni_xFe_yCo_{6-x-y}$)Mo_6C cuboids as outstanding bifunctional electrocatalysts for overall water splitting" *Appl. Catal. B-Environ.* (2021). **290**, 120049 10.1016/j.apcatb.2021.120049.
14. H. Ding, L. Xu, C. Wen, J.-J. Zhou, K. Li, P. Zhang, L. Wang, W. Wang, W. Wang, X. Xu, W. Ji, Y. Yang and L. Chen. "Surface and interface engineering of MoNi alloy nanograins bound to Mo-doped NiO nanosheets on 3D graphene foam for high-efficiency water splitting catalysis" *Chem. Eng. J.* (2022). **440**, 135847 10.1016/j.cej.2022.135847.
15. Y. Dou, C. T. He, L. Zhang, H. Yin, M. Al-Mamun, J. Ma and H. Zhao. "Approaching the activity limit of $CoSe_2$ for oxygen evolution via Fe doping and Co vacancy" *Nat. Commun.* (2020). **11**, 1664 10.1038/s41467-020-15498-0.
16. X. Chen, Q. Wang, Y. Cheng, H. Xing, J. Li, X. Zhu, L. Ma, Y. Li and D. Liu. "S-Doping Triggers Redox Reactivities of Both Iron and Lattice Oxygen in FeOOH for Low-Cost and High-Performance Water Oxidation" *Adv. Funct. Mater.* (2022). **32**, 2112674 10.1002/adfm.202112674.
17. L. Yu, L. Wu, B. McElhenny, S. Song, D. Luo, F. Zhang, Y. Yu, S. Chen and Z. Ren. "Ultrafast room-temperature synthesis of porous S-doped Ni/Fe (oxy)hydroxide electrodes for oxygen evolution catalysis in seawater splitting" *Energy Environ. Sci.* (2020). **13**, 3439–3446 10.1039/d0ee00921k.
18. X. Bai, M. Zhang, Y. Shen, X. Liang, W. Jiao, R. He, Y. Zou, H. Chen and X. Zou. "Room-Temperature, Meter-Scale Synthesis of Heazlewoodite-Based Nanoarray Electrodes for Alkaline Water Electrolysis" *Adv. Funct. Mater.* (2024). **34** 10.1002/adfm.202400979.
19. Z. Chen, X. Wang, S. Keßler, Q. Fan, M. Huang and H. Cölfen. "Synthesis of hierarchical transition metal oxyhydroxides in aqueous solution at ambient temperature and their application as OER electrocatalysts" *J. Energy Chem.* (2022). **71**, 89–97 10.1016/j.jechem.2022.02.042.
20. Y. Zhuo, D. Liu, L. Qiao, S. Chen, J. Lu, W. F. Ip, H. Pan and Z. Wang. "Ultrafast Room-Temperature Synthesis of Large-Scale, Low-Cost, and Highly Active Ni─Fe Based Electrodes toward Industrialized Seawater Oxidation" *Adv. Energy Mater.* (2023). **13** 10.1002/aenm.202301921.
21. Y. Zhao, Y. Gao, Z. Chen, Z. Li, T. Ma, Z. Wu and L. Wang. "Trifle Pt coupled with NiFe hydroxide synthesized via corrosion engineering to boost the cleavage of water molecule for alkaline water-splitting" *Appl. Catal. B-Environ.* (2021). **297** 10.1016/j.apcatb.2021.120395.
22. L. Ye, Z. Chen, X. Xu, F. Ma, K. Fan, L. Zong, L. Wang, G. Chen, X. Li and T. Zhan. "Ultrafast Room-Temperature Synthesis of Phosphate-Intercalated NiFe Layered Double Hydroxides for High-Performance Alkaline Seawater Oxidation" *Inorg. Chem.* (2024). **63**, 20859–20869 10.1021/acs.inorgchem.4c03660.

23. M. B. Dalla Benetta, E. Dempsey and C. B. Breslin. "Corrosion-Engineered Room-Temperature Deposition of NiMoO4/NiS/Ni(OH)2 at Nickel Foam: An Efficient Strategy and Electrocatalyst for the Oxygen Evolution Reaction" *ACS Appl. Energ. Mater.* (2024). **7**, 11703–11713 10.1021/acsaem.4c01627.
24. H. Zhang, Q. Jiang, J. H. L. Hadden, F. Xie and D. J. Riley. "Pd Ion-Exchange and Ammonia Etching of a Prussian Blue Analogue to Produce a High-Performance Water-Splitting Catalyst" *Adv. Funct. Mater.* (2020). **31** 10.1002/adfm.202008989.
25. Y. Chen, L. Hao, H. Ma, T. Zhang, H. Yan and Y. Zhang. "Design of cerium dioxide anchored in cobalt-iron layered double hydroxide hollow polyhedra via an ion exchange strategy for the oxygen evolution reaction" *J. Colloid Interface Sci.* (2025). **683**, 411–420 10.1016/j.jcis.2024.12.196.
26. Y. Ma, D. Leng, X. Zhang, J. Fu, C. Pi, Y. Zheng, B. Gao, X. Li, N. Li, P. K. Chu, Y. Luo and K. Huo. "Enhanced Activities in Alkaline Hydrogen and Oxygen Evolution Reactions on MoS(2) Electrocatalysts by In-Plane Sulfur Defects Coupled with Transition Metal Doping" *Small* (2022). **18**, e2203173 10.1002/smll.202203173.
27. C. Chen, Y. Tuo, Q. Lu, H. Lu, S. Zhang, Y. Zhou, J. Zhang, Z. Liu, Z. Kang, X. Feng and D. Chen. "Hierarchical trimetallic Co-Ni-Fe oxides derived from core-shell structured metal-organic frameworks for highly efficient oxygen evolution reaction" *Appl. Catal. B-Environ.* (2021). **287** 10.1016/j.apcatb.2021.119953.
28. F. Wu, X. Guo, G. Hao, Y. Hu and W. Jiang. "Synthesis of Iron–Nickel Sulfide Porous Nanosheets via a Chemical Etching/Anion Exchange Method for Efficient Oxygen Evolution Reaction in Alkaline Media" *Adv. Mater. Interfaces* (2019). **6** 10.1002/admi.201900788.
29. Y. Zhao, X. F. Lu, Z. P. Wu, Z. Pei, D. Luan and X. W. D. Lou. "Supporting Trimetallic Metal-Organic Frameworks on S/N-Doped Carbon Macroporous Fibers for Highly Efficient Electrocatalytic Oxygen Evolution" *Adv. Mater.* (2023). **35**, e2207888 10.1002/adma.202207888.
30. X. Yu, Z.-Y. Yu, X.-L. Zhang, P. Li, B. Sun, X. Gao, K. Yan, H. Liu, Y. Duan, M.-R. Gao, G. Wang and S.-H. Yu. "Highly disordered cobalt oxide nanostructure induced by sulfur incorporation for efficient overall water splitting" *Nano Energy* (2020). **71** 10.1016/j.nanoen.2020.104652.
31. H. Yang, Y. Duan, H. Gu, L. Liu, H. Guan, C. Dong and G. Chen. "In-Situ Synthesis Strategy of S-Doped Hierarchical Ni-MOF Nanosheet Supercapacitor Electrodes via Nickle Foam Etching" *ACS Appl. Energ. Mater.* (2023). **6**, 3789–3798 10.1021/acsaem.2c04024.
32. F. Wang, J. Y. Cheong, Q. He, G. Duan, S. He, L. Zhang, Y. Zhao, I.-D. Kim and S. Jiang. "Phosphorus-doped thick carbon electrode for high-energy density and long-life supercapacitors" *Chem. Eng. J.* (2021). **414**, 128767 10.1016/j.cej.2021.128767.
33. Y. Rao, M. Yuan, B. Gao, H. Li, J. Yu and X. Chen. "Laser-scribed phosphorus-doped graphene derived from Kevlar textile for enhanced wearable micro-supercapacitor" *J. Colloid Interface Sci.* (2023). **630**, 586–594 10.1016/j.jcis.2022.10.024.
34. W. Chen, T. Wei, L.-E. Mo, S. Wu, Z. Li, S. Chen, X. Zhang and L. Hu. "CoS_2 nanosheets on

carbon cloth for flexible all-solid-state supercapacitors" *Chem. Eng. J.* (2020). **400**, 125856 10.1016/j.cej.2020.125856.
35. P. Jia, H. Yao, Q. Liu, J. Wang, Z. Li and L. Zhang. "Construction of 3D carbon dots/Prussian blue analogue-derived phosphide nanocomposites on Ni foam for high-performance supercapacitors" *J. Alloy. Compd.* (2023). **958**, 170525 10.1016/j.jallcom.2023.170525.
36. R. Wang, S. Cai, Y. Yan, W. M. Yourey, W. Tong and H. Tang. "A novel high-performance electrode architecture for supercapacitors: Fe_2O_3 nanocube and carbon nanotube functionalized carbon" *J. Mater. Chem. A* (2017). **5**, 22648–22653 10.1039/c7ta07043h.
37. T. Liu, J. Mou, Z. Wu, C. Lv, J. Huang and M. Liu. "A Facile and Scalable Strategy for Fabrication of Superior Bifunctional Freestanding Air Electrodes for Flexible Zinc–Air Batteries" *Adv. Funct. Mater.* (2020). **30**, 2003407 10.1002/adfm.202003407.
38. Q. Lu, J. Yu, X. Zou, K. Liao, P. Tan, W. Zhou, M. Ni and Z. Shao. "Self-Catalyzed Growth of Co, N-Codoped CNTs on Carbon-Encased CoSx Surface: A Noble-Metal-Free Bifunctional Oxygen Electrocatalyst for Flexible Solid Zn–Air Batteries" *Adv. Funct. Mater.* (2019). **29**, 1904481 10.1002/adfm.201904481.
39. Q. Lv, N. Wang, W. Si, Z. Hou, X. Li, X. Wang, F. Zhao, Z. Yang, Y. Zhang and C. Huang. "Pyridinic nitrogen exclusively doped carbon materials as efficient oxygen reduction electrocatalysts for Zn-air batteries" *Appl. Catal. B-Environ.* (2020). **261**, 118234 10.1016/j.apcatb.2019.118234.
40. Z. Zhu, H. Yin, Y. Wang, C. H. Chuang, L. Xing, M. Dong, Y. R. Lu, G. Casillas-Garcia, Y. Zheng, S. Chen, Y. Dou, P. Liu, Q. Cheng and H. Zhao. "Coexisting Single-Atomic Fe and Ni Sites on Hierarchically Ordered Porous Carbon as a Highly Efficient ORR Electrocatalyst" *Adv. Mater.* (2020). **32**, 2004670 10.1002/adma.202004670.
41. H. Yang, S. Gao, D. Rao and X. Yan. "Designing superior bifunctional electrocatalyst with high-purity pyrrole-type CoN_4 and adjacent metallic cobalt sites for rechargeable Zn-air batteries" *Energy Storage Mater.* (2022). **46**, 553–562 10.1016/j.ensm.2022.01.040.
42. H. Xu, D. Wang, P. Yang, L. Du, X. Lu, R. Li, L. Liu, J. Zhang and M. An. "A hierarchically porous Fe-N-C synthesized by dual melt-salt-mediated template as advanced electrocatalyst for efficient oxygen reduction in zinc-air battery" *Appl. Catal. B-Environ.* (2022). **305**, 121040 10.1016/j.apcatb.2021.121040.
43. X. Wang, H. Zhu, C. Yang, J. Lu, L. Zheng and H.-P. Liang. "Mesoporous carbon promoting the efficiency and stability of single atomic electrocatalysts for oxygen reduction reaction" *Carbon* (2022). **191**, 393–402 10.1016/j.carbon.2022.01.057.
44. J. Lu, K. Huang, H. Lee, S. Huang, H. Fu, H. Wang, S. Liu, D. Min, C. Lian and H. S. Park. "Reverse Oriented Dual-Interface Built-in Electric Fields of Robust $Pd_1Mo_1Ta_2O_\alpha$ Bifunctional Electrocatalysis for Zinc-Air Batteries" *Adv. Funct. Mater.* (2024). **35**, 2418211 10.1002/adfm.202418211.
45. X. Zheng, X. Cao, K. Zeng, J. Yan, Z. Sun, M. H. Rummeli and R. Yang. "A Self-Jet Vapor-Phase

Growth of 3D FeNi@NCNT Clusters as Efficient Oxygen Electrocatalysts for Zinc-Air Batteries" *Small* (2021). **17**, 2006183 10.1002/smll.202006183.
46. K. Kim, K. Min, Y. Go, Y. Lee, S. E. Shim, D. Lim and S.-H. Baeck. "FeCo alloy nanoparticles embedded in N-doped carbon supported on highly defective ketjenblack as effective bifunctional electrocatalysts for rechargeable Zn–air batteries" *Appl. Catal. B-Environ.* (2022). **315**, 121501 10.1016/j.apcatb.2022.121501.
47. B. Chen, X. He, F. Yin, H. Wang, D. J. Liu, R. Shi, J. Chen and H. Yin. "MO-Co@N-Doped Carbon (M = Zn or Co): Vital Roles of Inactive Zn and Highly Efficient Activity toward Oxygen Reduction/Evolution Reactions for Rechargeable Zn–Air Battery" *Adv. Funct. Mater.* (2017). **27**, 1700795 10.1002/adfm.201700795.
48. X. Xu, J. Xie, B. Liu, R. Wang, M. Liu, J. Zhang, J. Liu, Z. Cai and J. Zou. "PBA-derived FeCo alloy with core-shell structure embedded in 2D N-doped ultrathin carbon sheets as a bifunctional catalyst for rechargeable Zn-air batteries" *Appl. Catal. B-Environ.* (2022). **316**, 121687 10.1016/j.apcatb.2022.121687.
